# Explaining Spatial Variation in the Recording Effort of Citizen Science Data across Multiple Taxa

**DOI:** 10.1371/journal.pone.0147796

**Published:** 2016-01-28

**Authors:** Louise Mair, Alejandro Ruete

**Affiliations:** 1 Species Information Centre, Swedish University of Agricultural Sciences (SLU), P.O. 7007, SE-750 07 Uppsala, Sweden; 2 Department of Ecology, Swedish University of Agricultural Sciences (SLU), P.O. 7004, SE-750 07 Uppsala, Sweden; University of Waikato (National Institute of Water and Atmospheric Research), NEW ZEALAND

## Abstract

The collation of citizen science data in open-access biodiversity databases makes temporally and spatially extensive species’ observation data available to a wide range of users. Such data are an invaluable resource but contain inherent limitations, such as sampling bias in favour of recorder distribution, lack of survey effort assessment, and lack of coverage of the distribution of all organisms. Any technical assessment, monitoring program or scientific research applying citizen science data should therefore include an evaluation of the uncertainty of its results. We use ‘ignorance’ scores, i.e. spatially explicit indices of sampling bias across a study region, to further understand spatial patterns of observation behaviour for 13 reference taxonomic groups. The data is based on voluntary observations made in Sweden between 2000 and 2014. We compared the effect of six geographical variables (elevation, steepness, population density, log population density, road density and footpath density) on the ignorance scores of each group. We found substantial variation among taxonomic groups in the relative importance of different geographic variables for explaining ignorance scores. In general, road access and logged population density were consistently important variables explaining bias in sampling effort, indicating that access at a landscape-scale facilitates voluntary reporting by citizen scientists. Also, small increases in population density can produce a substantial reduction in ignorance score. However the between-taxa variation in the importance of geographic variables for explaining ignorance scores demonstrated that different taxa suffer from different spatial biases. We suggest that conservationists and researchers should use ignorance scores to acknowledge uncertainty in their analyses and conclusions, because they may simultaneously include many correlated variables that are difficult to disentangle.

## Introduction

Species’ observation data collected through citizen science projects provide an increasingly valuable resource in conservation and research due to the extensive spatial and temporal coverage that can be achieved through volunteer participation [1, 2]. The simultaneous increase in the number of volunteer recording schemes and the use of mobile technology by recorders has made the collation of species observation data possible at regional and national scales. As a result, extensive observation data for a taxonomically diverse range of species across large spatial and temporal extents are now available on online databases (e.g. GBIF, eBird).

Such citizen science observation data have a wide range of applications. They have been used to create distribution maps for species atlases [3], to test the reliability of species’ range maps created before the availability of such widespread data [4], and to assess changes in species’ occupancy [5], abundance [6] and distributions in response to environmental change [7, 8]. Data can be used to quantify patterns of species’ richness [9], assess the efficacy of protected areas [10] and identify further priority areas for conservation [11]. Data can also be used to determine under- or un-sampled areas where a lack of species observations means that there is not enough information for conservation decision-making and therefore these areas can be targeted for species surveys [12]. A particularly widespread application is in species’ distribution modelling [13, 14], as relatively little expense is required to obtain extensive citizen science data whereas, in contrast, the rigorous collection of presence-absence data in planned scientific surveys is costly and time consuming [15].

In all these applications, however, the data users must be aware of and tackle the problems of bias which are inherent in citizen science data [16]. While there exists many recording schemes which apply particular (such as regular transect walks), a large volume of observation data is collected in an *ad hoc* manner. Data collected without a controlled effort, which we focus on in this study, contain many sources of recording bias. Volunteer recorders do not select survey sites randomly; they may be influenced by accessibility [17], proximity to their home [18] and the known species richness of a site, which makes it more or less attractive to survey [12]. Recorders tend to focus on particular taxonomic groups, but they may not record every species they see, either through a lack of identification skills [15], or a lack of interest in very common species [19–21]. This behaviour results in substantial spatial bias in citizen science datasets, with some areas receiving little or no survey effort while others are inundated, and so recording fails to cover the full distribution of organisms. Moreover, most uncontrolled-effort citizen science data are presence-only, and therefore it is unknown whether an absence of records in the dataset reflects an absence of the species, an absence of recorders or a lack of reporting. Indeed, without absence data the distributions of species cannot be readily teased apart from the distribution of recorders [22]. A lack of absence data combined with an unknown survey effort (or a description of survey effort that is difficult to quantify) makes such biases particularly difficult to account for in statistical modelling.

As a consequence, a method for quantifying how much recording effort a given location has received for a particular organism based upon presence-only observation records is required. The incorporation into analyses of information on how recording effort varies spatially can result in improved species distribution models [23] and allows more accurate assessment of species richness, as well as the identification of under-sampled areas where the absence of species observations are less likely to be true species absences. We therefore use ‘ignorance’ scores, which are spatially explicit indices of the bias and lack of sampling effort across a study region [24]. In this way, the number of observations per area unit (e.g. grid cell) is transformed to ignorance scores on a scale of zero to one (one being absolute ignorance and zero being absolute certainty or credibility in the data). Ignorance scores are calculated from the raw citizen science observation data of a reference taxonomic group and allow identification of the areas where there is least (or conversely most) confidence that absences of observations in the dataset are true species absences. An ignorance score map could therefore inform users of under-sampled areas to be targeted for surveys, or be applied in species distribution modelling as a confidence or bias layer [25].

Ignorance scores have been developed as a tool to be available on species observation databases in order to allow users to assess the quality of the data before carrying out analyses [26]. Our aim was to compare the effect of six geographical variables (elevation, steepness, population density, log-population density, road density and footpath density) on the ignorance score among thirteen reference taxonomic groups. Previous studies that have examined patterns of observations have found strong roadside biases within woody plant records [27] and have shown that collection patterns differ between butterflies and mammals [28]. We expand upon current knowledge by assessing patterns of recorder behaviour across multiple taxa for the full extent of Sweden. We demonstrate how ignorance scores can be used to assess the behaviour of recorders, and then discuss the wider implications for the application of ignorance scores in species distribution modelling and survey planning.

## Methods

### Species data and calculation of ignorance scores

Species’ observation data were obtained from Swedish LifeWatch, which is a national collaboration between universities, natural history museums and public authorities. The aim of Swedish Lifewatch is to collate species observation data from many different resources and make the data available from a single web-based portal (www.svenskalifewatch.se). Thus temporal and spatial data on species’ observations can be downloaded for a wide range of taxonomic groups, and it is possible to filter data based on a range of criteria (for example date, spatial resolution or recording scheme).

**Fig 1:**
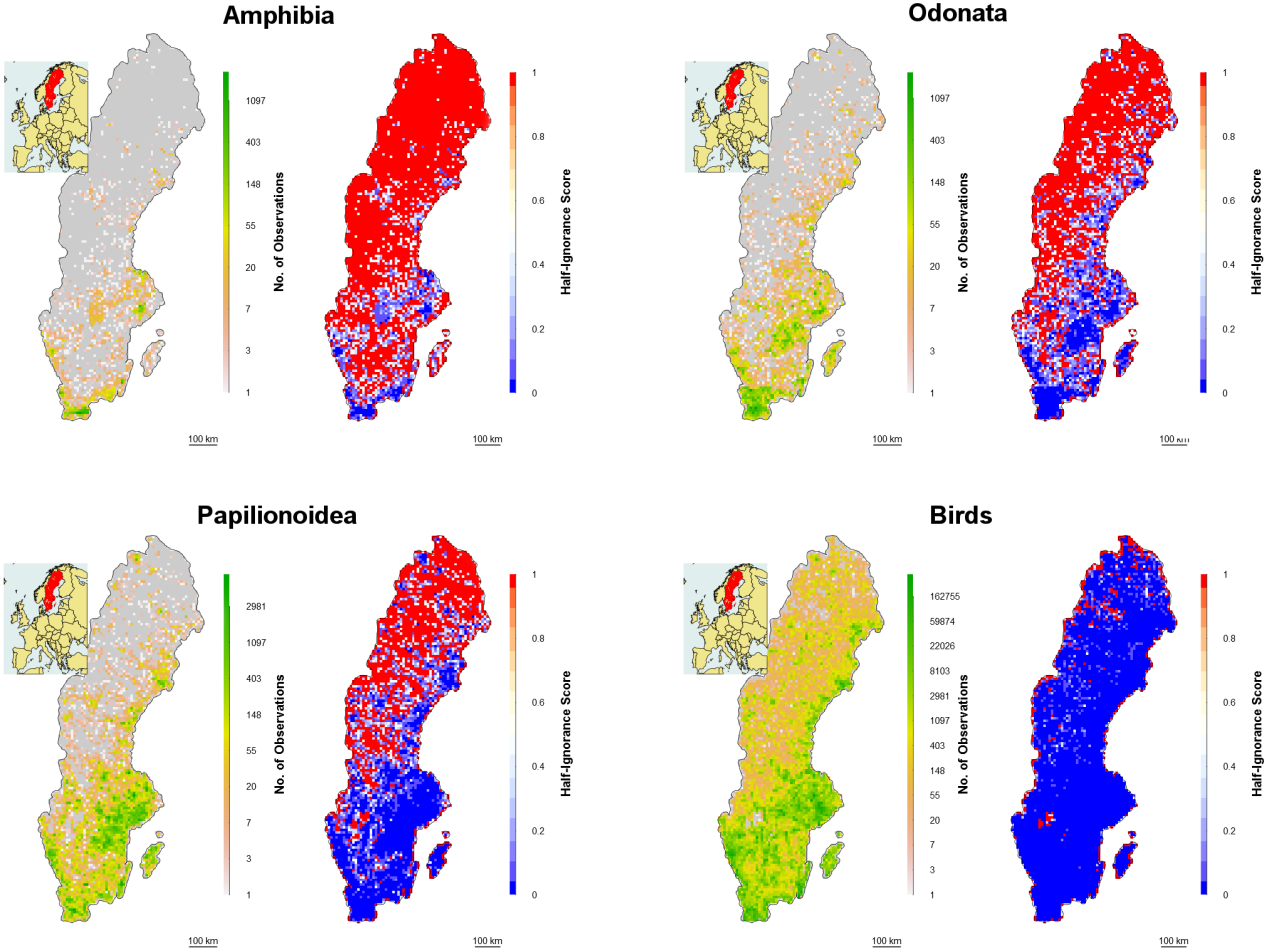
Logged number of observations over Sweden and ignorance maps for the period 2000–2014. Logged number of observations over Sweden for the period 2000–2014 (where grey = no observations) and ignorance maps produced with the half-ignorance algorithm (O_0.5_ = 1) for three taxonomic groups with widely varying numbers of records; Amphibia (N = 26,794), Odonata (N = 100,367), Papilionoidea (N = 505,881) and birds (N = 22,331,191). Grid resolution is 10 x 10 km. The black contour shows a 10 km buffer around Sweden’s land surface. The inset shows the location of Sweden in Europe.

We generated ignorance scores for 13 taxonomic groups (Aracnidae, Coleoptera, Opilionidae, Odonata, Papilionoidea, Bryophyta, fungi, lichen, Amphibia, Aves, land mammals (excluding bats), vascular plants/Tracheophyta, and Poaceae) based on summarized data on the number of observation records over a 10x10 km square grid (4829 grid cells) in Sweden for the period 2000–14. The total number of observations varied greatly among taxonomic groups, with Aves (birds) being the most heavily recorded group and Opilionidae (harvestmen) being the least recorded (Table 1). Ignorance scores for each species were calculated for each 10 km grid square according to the so-called half-ignorance algorithm: *O*_*0*.*5*_/(*N*_i_+ *O*_*0*.*5*_), where *N* is the number of records in a grid square *i* and *O*_*0*.*5*_ is the number of observations considered to be enough to reduce the ignorance score by half (Lepidoptera are shown as an example in Fig 1, for maps of all 13 taxonomic groups see Fig A in S1 File)[26]. Here we vary *O*_*0*.*5*_ through a wide range of values (*O*_*0*.*5*_ = 1, 2, 5, 10, 20 and 50). When *O*_*0*.*5*_ = 1, this means that one observation is enough to consider that the absence of reports of a target species from any grid cell is 50% due to true absence from the site and 50% due to failure to detect the species given that one observer has been present. Thus by varying *O*_*0*.*5*_ we test the sensitivity of our results to changes in this threshold value.

**Table 1:** The total number of observations across Sweden for each of the 13 taxonomic groups studied for the period 2000–14, the number of species in each taxonomic group, and the number of observations contributed by the 1^st^ recorder and the 20^th^ recorder (ranked by number of observations contributed).

Taxa	Common name	Total number of observations	Number of species	Number of observations 1^st^ recorder	Number of observations 20^th^ recorder
Araneae	Spiders	40,028	691	92802	243
Coleoptera	Beetles	888,094	4482	36027	8316
Opilionidae	Harvestmen	907	20	685	22
Odonata	Dragonflies & damselflies	100,367	64	4557	983
Papilionoidea	Butterflies	505,881	121	7912	3407
Amphibia	Amphibians	26,794	14	7150	290
Aves	Birds	22,331,191	527	NA	NA
Land Mammals	Land mammals	21,578	55	1339	369
Bryophyta	Mosses	340,782	774	39590	4202
Fungi	Fungi	935,615	5976	223084	13182
Lichens	Lichens	294,432	1659	39395	3789
Tracheophyta	Vascular plants	3,906,801	4366	611435	32816
Poaceae	Flowering plants	356,400	221	61829	3392

### Geographic variables

**Fig 2:**
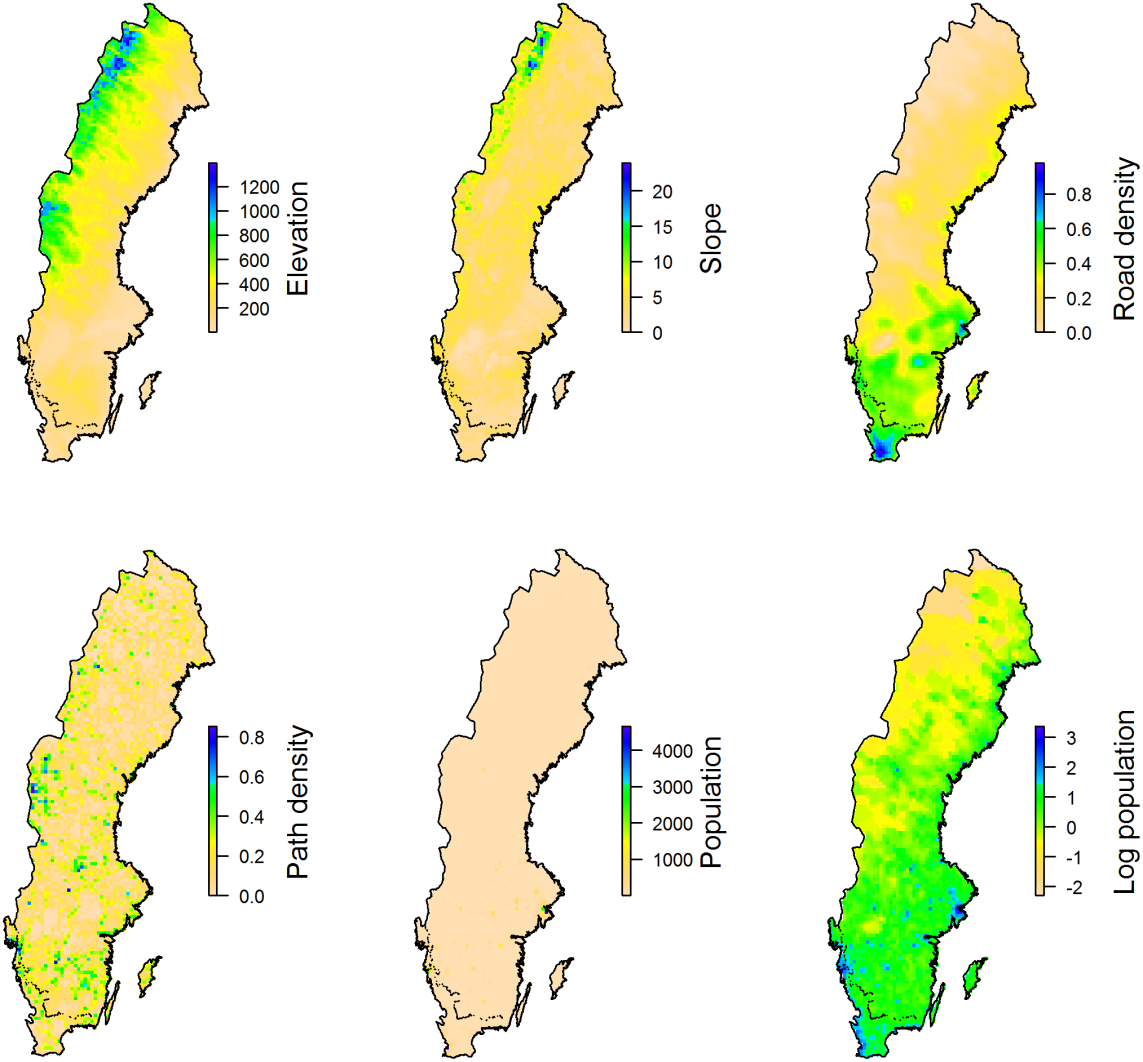
Maps of the geographic variables used to explain ignorance. The geographic variables used to explain ignorace (elevation, slope, road density, path density, population density and log population density) in Sweden at the 10 km grid cell resolution.

We identified six geographical variables that had the potential to explain variation in ignorance scores; these were elevation, steepness, human population density, log human population density, road density and footpath density (Fig 2). We selected potential explanatory geographical variables based on existing knowledge of observer behaviour bias. Accessibility has been shown to be important in determining where volunteers record [18, 28], and so we selected variables that reflect multiple aspects of accessibility; road density reflects accessibility to a site, and path density and steepness reflect accessibility within a site. Elevation was selected as mountainous areas tend to be less accessible in all of these respects. We also considered human population density as a likely indication of the number of recorders available in the area, while the log of population density allowed us to consider specifically the effect of changes in population at very low densities. All variables were utilized as raster layers covering the extent of Sweden at a 10 km resolution and were generated using ArcMap 10.2.2.

Elevation was aggregated from a 50 m resolution digital elevation map to give the mean elevation in meters at a 10 km resolution (elevation data from the Swedish land survey service, www.lantmateriet.se). Steepness was calculated by applying the *slope* tool in ArcMap to a 50 m resolution digital elevation map, which was then aggregated to 10 km resolution to give the mean steepness in percent per 10 km grid cell.

Population data were available for the year 2010 at the resolution of ‘Small Areas for Market Statistics’, these are areas consisting of one or more postcodes and are therefore finer than the county level (population data from Statistika centralbyrån, www.scb.se). Polygons of population per area were used to calculate the population density per 1 km grid square (as the number of people per 1km square) and these data were then aggregated to a 10 km grid cell resolution to give the mean number of people/km^2^. Given the large range of population densities across Sweden (varying from >40,000 people/km^2^ in parts of Stockholm to <1 person/km^2^ in parts of Norrbotten, the northernmost county) and that most of the country’s land area has low population densities, we also calculated the log of population density in order to explore the effects of changes in population at the lowest densities.

Road density was calculated from a road map feature from 1999 (Swedish land survey service; 1999 was selected to avoid inclusion of roads that were only built towards the end of our study period) using the *line density* tool in ArcMap at a 10 km resolution. In Sweden, a dense network of forestry roads has been built to provide efficient transportation for the industry, and inclusion of these roads in our analyses would mask the importance of the major road network used by the general public, therefore we excluded forestry access roads.

Path density was calculated from a path map feature, also from 1999 (Swedish land survey service), using the *line density* tool in ArcMap at a 10 km resolution. We included footpaths, hiking trails and hiking trails along footpaths in order to capture the path network that best reflected access to the countryside, rather than pedestrian access within urban areas.

### Statistical analyses

To model effects of the proposed geographical explanatory variables on the ignorance score (*I*_*i*_), we applied the Bayesian generalized linear modelling approach assuming *I*_*i*_ followed a Beta probability distribution as

$$I_{i}\sim Beta\left(a_{i},b_{i}\right)$$

$$a_{i}=\mu_{i}\cdot \varphi$$

$$b_{i}=(1-\mu_{i})\cdot \varphi$$

$$\text{logit}(\mu_{i})=\alpha +X_{i}\beta ,$$

where α is the intercept parameter, X_i_ is a matrix of explanatory variables, β is a vector of associated effect size parameters. We tested for both linear and quadratic effects of the covariates in X_i_. We set uninformative priors (i.e. φ ~ Gamma(0.1, 0.1); α and β ~ Normal(0, 1000), and sampled the models 5000 iterations after convergence. Models were fitted in JAGS 3.0 [29]. We used the difference in the model deviance (Δ Deviance) compared to nested models as a measure of model fit [30]. For each variable we first compared linear effects against the null model, and then the inclusion of quadratic effects against the linear models. Because the differences in deviance follow a Chi^2^ probability distribution a minimum difference of 3.84 is required for a variable to improve the model fit when one parameter is added. We also calculated for each model the percentage of explained variation (R^2^) from the model deviance.

## Results

**Fig 3:**
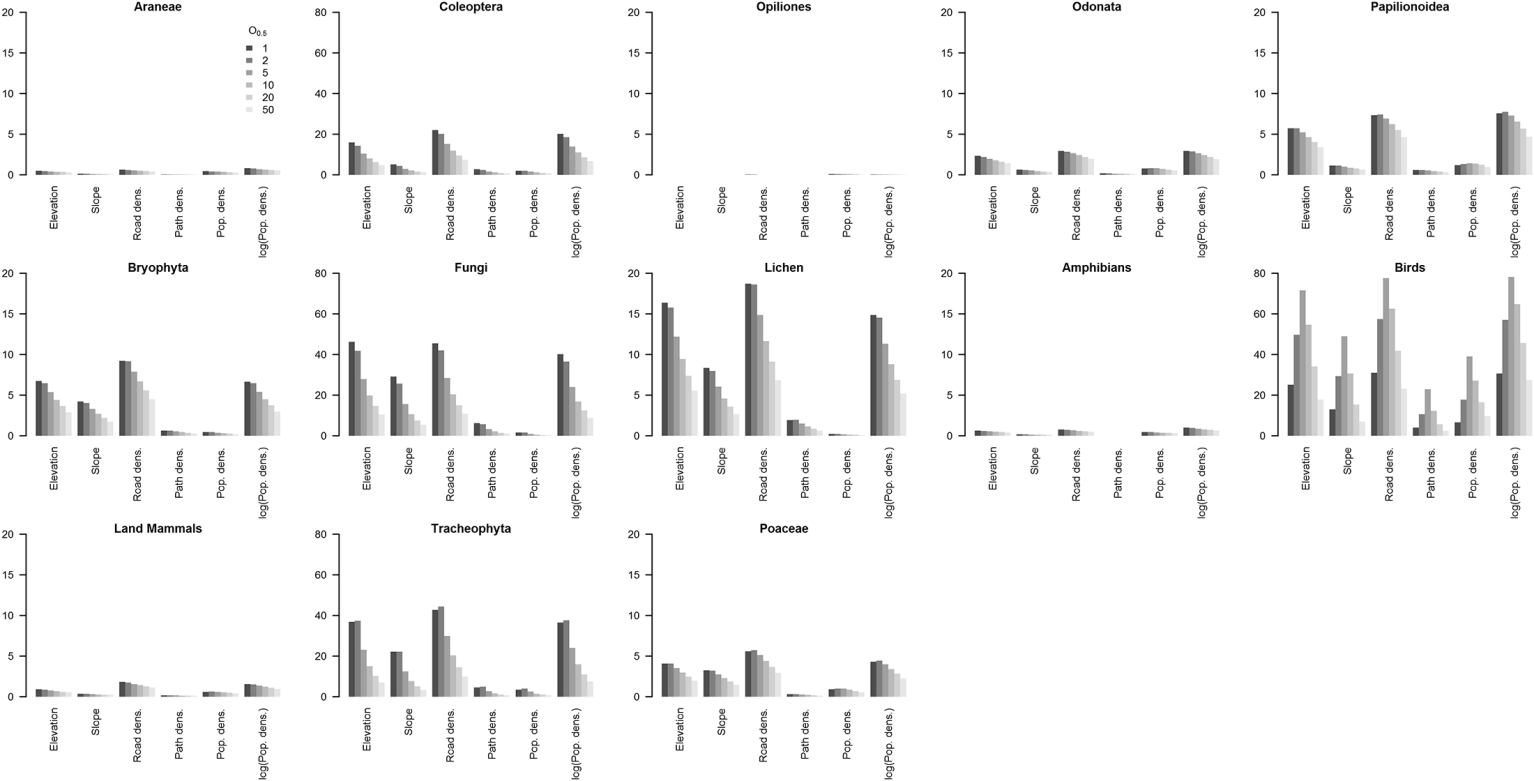
The percent deviance explained (R^2^) when each geographic variable was modelled independently against ignorance score. The percent deviance explained (R^2^) when each geographic variable was modelled independently against ignorance score at varying levels of O_0.5_ (shading indicates the gradient from O_0.5_ = 1 at the darkest, through 2, 5, 10, 20 to 50 at the lightest). R^2^ was calculated for the best fit model in each case (either linear or quadratic depending on Δ deviance; Tables A and B in S1 File). Note that the range of y-axes varies (a maximum of either 20 or 80) to accommodate the large variation amongst taxa in R^2^ values obtained.

The amount of variation in ignorance explained (R^2^) by the six geographic variables studied (elevation, steepness, population density, log population density, road density and footpath density) varied substantially among taxonomic groups (Fig 3; see Table A in S1 File for R^2^ values and Table B in S1 File for full model details). No single geographic variable consistently explained the most variation across taxonomic groups, however road density, the log of population density and elevation were consistently the top three variables explaining the most variation in ignorance across all 13 taxonomic groups. The relative importance of each explanatory variable remained relatively consistent within each taxonomic group as O_0.5_ increased, however for most taxa the amount of variation explained decreased with increasing O_0.5_ (Fig 3). One notable exception was for birds, where explained deviance peaked at O_0.5_ = 5.

The percent variation explained varied among taxonomic groups (Fig 3), and was lower for species with low sampling effort (Table 1). This was partly due to the limited spatial coverage of records for the taxonomic groups with low sampling effort (see maps in Fig 1 and Fig A in S1 File), resulting in less geographic variability being covered by the samples. Despite this, the models were able to detect significant effects of geographic variables, even for the poorly sampled taxa (Tables A and B in S1 File).

**Fig 4:**
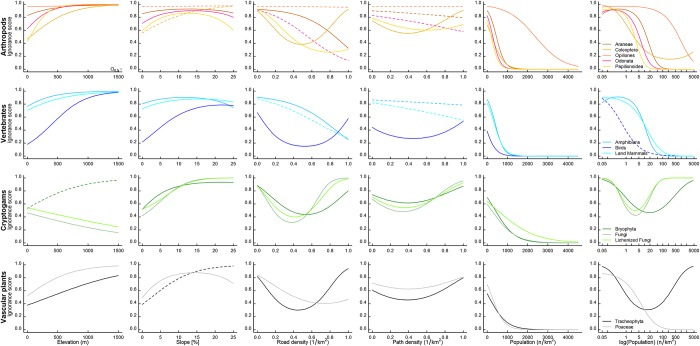
The relationship between ignorance score and environmental variables. The relationship between ignorance score and six environmental variables: elevation, slope, road density, path density, population density and log population density, for each taxonomic group studied when O_0.5_ = 1. Solid line indicates quadratic relationship, while dashed line indicates that only a linear relationship improved the model fit compared to the null model (only best models are drawn).

In order to determine the direction of the effect of each geographic variable on ignorance, we plotted the fitted curves of ignorance against each geographic variable for our taxonomic groups. Fig 4 shows the direction of the effect of the geographic variables on ignorance for a threshold value of O_0.5_ = 1 (for all other values of O_0.5_ see Fig B in S1 File) and indicates among taxa variation. For example, it can be seen that for Opiliones, ignorance remained high regardless of the change in road density, thus road density did little to explain variation in ignorance for this taxonomic groups. On the other hand, ignorance decreased substantially for Odonata and Araneae as road density increased, demonstrating the importance of this variable in explaining ignorance for these groups. Road density also explained a large amount of variation for Coleoptera, but for this taxon the lowest ignorance was observed at intermediate road densities.

There are some general trends that emerge from Fig 4. Increasing population density consistently reduced the ignorance score, indicating, as would be expected, that a higher population density resulted in greater recording effort. However, logged population density generally explained more variation (Fig 3) but did not show such consistent patterns across taxa, with the lowest ignorance scores being achieves at intermediate population densities for some taxa. Increasing steepness generally resulted in greater ignorance, revealing that steeper slopes were less well sampled. Increasing elevation generally resulted in greater ignorance, except for fungi and lichenized fungi which, on the contrary, showed reduced ignorance at higher elevations.

The effects of road density and path density showed slightly more complex trends amongst taxonomic groups. For spiders and butterflies, ignorance decreased as road density increased, and the same trend was found in amphibians and land mammals. On the other hand, beetles, plants, fungi, mosses and birds showed the lowest ignorance scores at intermediate road densities, suggesting that these groups were less well recorded in both the most urbanized and the most remote areas. Path density generally explained little variation in ignorance scores, particularly for Coleoptera and Opiliones, but there was a slight trend for decreasing ignorance as path density increased for Araneae, Odonata, amphibians and land mammals. The remaining taxonomic groups (birds, plants, Papilionidea, bryophytes, fungi and lichenized fungi) showed the lowest ignorance scores at intermediate path densities, suggesting that recording was lower at either extreme of path density.

From Fig 4 we can also elucidate ‘threshold’ values for obtaining an ignorance score of 0.5, which, for O_0.5_ = 1, is the score at which at least one observer is present recording those species in the taxonomic group, and at ignorance scores lower than 0.5 there is more than one recorder present. Determining when this threshold is reached for different geographic variables among the taxonomic groups gives an indication of how recording effort varies amongst taxonomic groups. For example, for both amphibians and Papilionidea, the log of population density explained a large part of the variation in ignorance scores (Fig 3) and for both taxa, ignorance scores decreased as log population density increased (Fig 4), however Fig 4 also shows that the 0.5 threshold was reached at a density of around 3 people/km^2^ for Papilionidea, while a considerably higher density of around 14 people/km^2^ was required for amphibians to reach the threshold. Thus Papilionidea were likely to be better recorded at much lower population densities than amphibians were. For birds and land mammals, the model including road density fitted best, and we can see that only at the very extremes of lowest and highest road densities was the threshold not reached for birds, while for land mammals, the threshold was not reached below a road density of around 0.7/km^2^.

## Discussion

We used an ‘ignorance score’ to quantify the spatial recording effort of species’ observations across 13 taxonomic groups in Sweden, and related these scores to six geographic variables in order to explain spatial gradients in recording effort. We found substantial variation among taxonomic groups in the amount of variation in ignorance score explained by the different geographic variables, and the shape and directions of the relationship between each geographic variable and the ignorance score. The models were able to detect significant effects even for poorly studied taxa (for example Opiliones), providing that relationships existed between the geographic variable and ignorance score. The percent deviance explained was larger for species with greater sampling effort as the relationship between geographic variables and ignorance score was stronger at higher recording effort, yet low sampling effort did not prevent relationships from being detected. This result serves to highlight the advantage of using ignorance scores, or similar transformation of raw data, that allow differentiation between cells with information on the species and cells without.

Despite the variation between species, we were able to determine some general patterns. Road density, elevation and log population density were consistently ranked as the top three variables with the highest R^2^ values across all taxonomic groups. The direction of the relationship between road density and ignorance score indicated that ignorance was highest at low road densities. This suggests that accessibility at the landscape scale is more important to recorders than access at the local scale, particularly given that the density of paths (an indicator of local-scale accessibility) explained less variation in ignorance compared to the density of roads. In other words, the ability of recorders to access a site by car or public transport appears to be more important than the network of paths available once a site is reached. Ignorance score was also highest at very low population densities. The generally greater importance of log population density relative to absolute population density suggests that a substantial reduction in ignorance was achieved with a small increase in population density in the most sparsely populated areas. These results confirm that accessibility of sites was an important determinant of their ignorance score [17], and also that more remote areas had higher ignorance scores.

Importantly, these general patterns were consistent as O_0.5_ (the number of observations considered to be enough to reduce the ignorance score by half) was varied through a wide range of thresholds. The selection of the most appropriate value for O_0.5_ is both taxon and study dependant, and would be expected to vary depending on the spatial resolution of the study, as well as the methods used for collecting observations. For example, methods based on direct observations are biased towards rare and population species [20, 21]. Indeed, our results indicated that species’ observations of different taxonomic groups were collected in different ways. We found that not only did the amount of variation in ignorance explained by the geographic variables vary among taxonomic groups, but the direction of the relationship between ignorance and the geographic variables also varied among taxonomic groups. Our results support and expand upon a previous study comparing butterfly and mammal collections, which found spatial variation in the highest densities of collections between the two groups [28]. Observations of different taxonomic groups are collected in very different ways (for example trapping versus distance sampling) and our results indicate that this behavioural difference is borne out in different spatial patterns of recording effort among taxonomic groups. Bias layers could be refined even further by considering the methods used to record particular species-groups, for example nocturnal could be separated from diurnal birds, as nocturnal moths and day-flying butterflies are differentiated.

Our findings are of particular interest in terms of species distribution modelling, as they suggest that within the same study region, spatial biases in recording effort can be expected to vary among taxonomic groups, and so geographic variables that are incorporated into SDMs in order to account for recording bias must be chosen carefully and are likely to be taxon-specific. It may be that an index of recording effort created from records of the study species’ respective taxonomic group, such as the ignorance scores studied here, produces a more suitable bias layer than geographic variables, and indeed our results suggest that a preliminary analysis to determine which geographic variables are important determinants of taxonomic recording effort would be necessary. It is expected that geographical variables are highly correlated to each other (e.g. population density and altitude). Therefore, a single bias layer with ignorance scores (i.e. ignorance map) combines all potentially entangled effects of many geographical variables.

The use of an ignorance score based on taxonomic group sampling has some superficial similarities with the ‘target group background’ (TGB) approach to species distribution modelling that has been proposed as a solution to biased presence-only data [31], however we highlight an important methodological difference. The TGB approach involves using observations of related taxa as pseudo-absence points in modelling in order to achieve the same recording biases in both presence and background/pseudo-absence points [32]. Despite indications that this method performs well [32, 33], it has received criticism for simply replacing observer bias with species richness bias [34] and it may not work well in all applications [35]. In contrast, the use of an ignorance score focusses on recording effort and differentiates between areas with similar species richness but differing recording effort, which the TGB approach does not. We suggest that it would be more informative, and more flexible, to use observations of the wider taxonomic group to develop a spatial bias layer such as an ignorance score, and incorporate this layer into the model as an explanatory variable. In this way the model is explicitly accounting for uncertainty, which can improve model predictions [23]. Moreover, maps produced from such SDMs can indicate which areas of the study region are most affected by under-sampling and therefore have the greatest predictive uncertainty.

We emphasize that a high ignorance score can mean one of two things: given that there were few or no recorders on a site to observe related species, the species may either have been present but not detected, or have been truly absent. It is for this reason that the use of related taxa observations as pseudo-absence points in species modelling is problematic; the unsampled area of the landscape is simply disregarded and the uncertainty associated with a lack of observations is not recognised [23]. For example, plants, mosses, fungi and birds had high ignorance scores at very high road densities, indicating that these groups had few observation records in the most urbanized areas. This could either be due to a lack of recording in urban areas, or due to the species being absent in these environments. In terms of species distribution modelling, this can be acknowledged and addressed by explicitly modelling and then mapping uncertainty, for which we suggest ignorance scores are an appropriate tool. Furthermore, for conservationists and other practitioners, it is often necessary to carry out further sampling in order to reduce, rather than simply acknowledge, this uncertainty. By combining ignorance scores with other geographic variables, conservationists can make informed decisions about the areas which require further sampling. They could make the ecologically informed decision that only the most ecologically natural under-recorded areas are worth further sampling, as these will be the areas most likely to be targets for conservation efforts.

We suggest that the application of metrics such as ignorance scores has the potential to effectively tackle recording effort biases in observation data, however we acknowledge that such metrics cannot capture every facet of recording bias. It is not possible to ascertain the skills and survey efforts of individual recorders, and such detail could produce a more informative metric. Nevertheless we consider the use of ignorance scores to be a good solution when the only data available are the raw species’ observation records. Furthermore, ignorance scores are deliberately generic, allowing data from a wide range of sources to be considered simultaneously, without concern for the particular methods used to obtain the observation records [26]. Ignorance scores focus on spatial bias in observation data, however temporal variation in recording effort is a common feature in citizen science data and is an important consideration in, for example, studies of phenology [36]. Methods for dealing with temporal variation will depend upon the research question being asked, and ignorance scores have the potential to aid in mapping the spatial changes in recording effort over time.

Dealing with uncertainty in presence-only citizen science data is necessary for a wide range of applications, and the development of an ignorance score as implemented here provides an appropriate scale to compare different taxa, and a straight forward and easily interpretable method of doing so. We have shown among taxa variation in the importance of geographic variables for explaining ignorance scores, demonstrating that different taxa suffer from different spatial biases. We suggest that given such information, conservationists and researchers could use ignorance scores to incorporate uncertainty into their analyses and produce results and conclusions that acknowledge the strengths and weaknesses in their data.

## Supporting Information

S1 File Additional maps, figures and tables of results. This file contains Table A (Percent deviance explained (R^2^) for all 13 reference taxonomic groups), Table B (Detailed model results for all 13 reference taxonomic groups), Fig A (Logged number of observation maps and ignorance score maps for all 13 reference taxonomic groups) and Fig B (The relationship between ignorance score and environmental variables).
(DOCX)

## References

[1] Schmeller DS, Henry PY, Julliard R, Gruber B, Clobert J, Dziock F, et al Advantages of Volunteer-Based Biodiversity Monitoring in Europe. Conservation Biology. 2009;23(2):307–16. 10.1111/j.1523-1739.2008.01125.x 19183201

[2] Silvertown J. A new dawn for citizen science. Trends in Ecology & Evolution. 2009;24(9):467–71. 19586682 10.1016/j.tree.2009.03.017

[3] Asher J, Warren M, Fox R, Harding P, Jeffcoate G, Jeffcoate J. The Millennium Atlas of Butterflies in Britain and Ireland. Oxford: Oxford University Press; 2001.

[4] Ficetola GF, Rondinini C, Bonardi A, Katariya V, Padoa-Schioppa E, Angulo A. An evaluation of the robustness of global amphibian range maps. Journal of Biogeography. 2014;41(2):211–21.

[5] Ward EJ, Marshall KN, Ross T, Sedgley A, Hass T, Pearson SF, et al Using citizen-science data to identify local hotspots of seabird occurrence. PeerJ. 2015;3:e704 10.7717/peerj.704 25653898 PMC4304867

[6] Isaac NJB, Girardello M, Brereton TM, Roy DB. Butterfly abundance in a warming climate: patterns in space and time are not congruent. Journal of Insect Conservation. 2011;15(1–2):233–40.

[7] Breed GA, Stichter S, Crone EE. Climate-driven changes in northeastern US butterfly communities. Nature Climate Change. 2013;3(2):142–5.

[8] Mair L, Thomas CD, Anderson BJ, Fox R, Botham M, Hill JK. Temporal variation in responses of species to four decades of climate warming. Global Change Biology. 2012;18(8):2439–47.

[9] Lemoine N, Schaefer H-C, Böhning-Gaese K. Species richness of migratory birds is influenced by global climate change. Global Ecology and Biogeography. 2007;16(1):55–64.

[10] Devictor V, Godet L, Julliard R, Couvet D, Jiguet F. Can common species benefit from protected areas? Biological Conservation. 2007;139(1–2):29–36.

[11] Hurlbert AH, Jetz W. Species richness, hotspots, and the scale dependence of range maps in ecology and conservation. Proceedings of the National Academy of Sciences. 2007;104(33):13384–9. 10.1073/pnas.0704469104 PMC1948922 17686977

[12] Tulloch AIT, Mustin K, Possingham HP, Szabo JK, Wilson KA. To boldly go where no volunteer has gone before: predicting volunteer activity to prioritize surveys at the landscape scale. Diversity and Distributions. 2013;19(4):465–80.

[13] Barbosa FG, Schneck F. Characteristics of the top-cited papers in species distribution predictive models. Ecological Modelling. 2015;313:77–83.

[14] Keil P, Wilson AM, Jetz W. Uncertainty, priors, autocorrelation and disparate data in downscaling of species distributions. Diversity and Distributions. 2014;20(7):797–812.

[15] Dickinson JL, Zuckerberg B, Bonter DN. Citizen Science as an Ecological Research Tool: Challenges and Benefits. Annual Review of Ecology, Evolution, and Systematics. 2010;41:149–72.

[16] Rocchini D, Hortal J, Lengyel S, Lobo JM, Jiménez-Valverde A, Ricotta C, et al Accounting for uncertainty when mapping species distributions: The need for maps of ignorance. Progress in Physical Geography. 2011;35(2):211–26.

[17] Romo H, Garcia-Barros E, Lobo JM. Identifying recorder-induced geographic bias in an Iberian butterfly database. Ecography. 2006;29(6):873–85.

[18] Dennis RLH, Thomas CD. Bias in butterfly distribution maps: the influence of hot spots and recorder's home range. Journal of Insect Conservation. 2000;4:73–7.

[19] Tulloch AIT, Szabo JK. A behavioural ecology approach to understand volunteer surveying for citizen science datasets. Emu. 2012;112(4):313–25.

[20] Snäll T, Kindvall O, Nilsson J, Pärt T. Evaluating citizen-based presence data for bird monitoring. Biological Conservation. 2011;144(2):804–10.

[21] van Strien AJ, van Swaay CAM, Termaat T. Opportunistic citizen science data of animal species produce reliable estimates of distribution trends if analysed with occupancy models. Journal of Applied Ecology. 2013;50(6):1450–8.

[22] Isaac NJB, van Strien AJ, August TA, de Zeeuw MP, Roy DB. Statistics for citizen science: extracting signals of change from noisy ecological data. Methods in Ecology and Evolution. 2014;5(10):1052–60.

[23] Stolar J, Nielsen SE. Accounting for spatially biased sampling effort in presence-only species distribution modelling. Diversity and Distributions. 2015;21(5):595–608.

[24] Ruete A. Displaying bias in sampling effort of data accessed from biodiversity databases using ignorance maps. Biodiversity Data Journal. 2015(3):e5361 10.3897/BDJ.3.e5361 26312050 PMC4549634

[25] Syfert MM, Smith MJ, Coomes DA. The effects of sampling bias and model complexity on the predictive performance of MaxEnt species distribution models. Plos One. 2013;8(2):10. 10.1371/journal.pone.0055158 PMC3573023 23457462

[26] Ruete A. Simple algorithms to display ignorance maps of raw distributional data accessed from biodiversity databases. under review.

[27] Kadmon R, Farber O, Danin A. Effect of roadside bias on the accuracy of predictive maps produced by bioclimatic models. Ecological Applications. 2004;14(2):401–13.

[28] Fernández D, Nakamura M. Estimation of spatial sampling effort based on presence-only data and accessibility. Ecological Modelling. 2015;299(0):147–55.

[29] Plummer M. JAGS: A program for analysis of Bayesian graphical models using Gibbs sampling. 2012,http://mcmc-jags.sourceforge.net.

[30] Spiegelhalter DJ, Best NG, Carlin BR, van der Linde A. Bayesian measures of model complexity and fit. Journal of the Royal Statistical Society Series B-Statistical Methodology. 2002;64:583–616.

[31] Phillips SJ, Dudik M. Modeling of species distributions with Maxent: new extensions and a comprehensive evaluation. Ecography. 2008;31(2):161–75.

[32] Phillips SJ, Dudik M, Elith J, Graham CH, Lehmann A, Leathwick J, et al Sample selection bias and presence-only distribution models: implications for background and pseudo-absence data. Ecological Applications. 2009;19(1):181–97. 19323182 10.1890/07-2153.1

[33] Fithian W, Elith J, Hastie T, Keith DA. Bias correction in species distribution models: pooling survey and collection data for multiple species. Methods in Ecology and Evolution. 2015;6(4):424–38. 27840673 10.1111/2041-210X.12242 PMC5102514

[34] Warton DI, Renner IW, Ramp D. Model-Based Control of Observer Bias for the Analysis of Presence-Only Data in Ecology. Plos One. 2013;8(11). 10.1371/journal.pone.0079168 PMC3832482 24260167

[35] Kramer-Schadt S, Niedballa J, Pilgrim JD, Schroder B, Lindenborn J, Reinfelder V, et al The importance of correcting for sampling bias in MaxEnt species distribution models. Diversity and Distributions. 2013;19(11):1366–79.

[36] McInerny GJ, Chen M, Freeman R, Gavaghan D, Meyer M, Rowland F, et al Information visualisation for science and policy: engaging users and avoiding bias. Trends in Ecology & Evolution. 2014;29(3):148–57. 24565371 10.1016/j.tree.2014.01.003

