# Supplementary material for: Explaining Spatial Variation in the Recording Effort of Citizen Science Data across Multiple Taxa
Source: PLoS One. 2016 Jan 28;11(1):e0147796. doi: 10.1371/journal.pone.0147796 (PMC4731209; doi:10.1371/journal.pone.0147796)

**S1 File**

**Table A. Percent deviance explained (R^2^) for all 13 reference taxonomic groups**. R^2^ each geographic variable was modelled independently against ignorance score for all 13 taxonomic groups at all levels of O_0.5_.

**Table B. Detailed model results for all 13 reference taxonomic groups**. Deviance and mode of parameter estimates for each model for all levels of O_0.5_.

**Figure A**. **Logged number of observation maps and ignorance score maps for all 13 reference taxonomic groups.** Logged number of observations of each taxonomic group over Sweden for the period 2000-14 and ignorance maps produced with the half-ignorance algorithm. Grid resolution is 10 x 10 km. The black contour shows a 10 km buffer around Sweden’s land surface. The inset shows the location of Sweden in Europe.

**Figure B. The relationship between ignorance score and environmental variables.** The relationship between ignorance score and six environmental variables: elevation, slope, road density, path density, population density and log population density, for each taxonomic group studied at each level of O_0.5_ studied. Solid line indicates quadratic relationship, while dashed line indicates that only a linear relationship improved the model fit compared to the null model (only best models are drawn).

**S1 File Table A**

**Table A. Percent deviance explained (R^2^) for all 13 reference taxonomic groups**. R^2^ when each geographic variable was modelled independently against ignorance score for each taxonomic group at each level of O_0.5_.

(a) O_0.5_ = 1.

|  | **R^2^** | | | | | | | | | | | | |  |
| --- | --- | --- | --- | --- | --- | --- | --- | --- | --- | --- | --- | --- | --- | --- |
|  | Araneae | Coleaoptera | Opilindae | Odonata | Papilionoidea | Amphibian | Birds | Land mammals | Bryophyta | Fungi | Lichen | Tracheophyta | Poacea | |
| Elevation | 0.34 | 15.86 | 0.01 | 1.88 | 4.27 | 6.73 | 41.86 | 14.22 | 0.46 | 24.45 | 0.81 | 36.44 | 3.94 | |
| Elevation^2 | 0.49 | 15.93 | 0.02 | 2.32 | 5.75 | 6.74 | 46.22 | 16.36 | 0.66 | 25.15 | 0.9 | 36.91 | 4.09 | |
| Slope | 0.11 | 5.07 | 0 | 0.55 | 0.92 | 4.11 | 28.98 | 8.09 | 0.14 | 12.75 | 0.34 | 22.02 | 2.86 | |
| Slope^2 | 0.14 | 5.08 | 0.01 | 0.63 | 1.16 | 4.22 | 29.25 | 8.34 | 0.19 | 13.04 | 0.37 | 22.11 | 3.26 | |
| Road density | 0.63 | 18.09 | 0.03 | 2.93 | 7 | 7.97 | 31.13 | 13.35 | 0.78 | 26.43 | 1.81 | 34.5 | 5.31 | |
| Road density^2 | 0.66 | 22 | 0.03 | 2.93 | 7.32 | 9.2 | 45.45 | 18.7 | 0.79 | 31.04 | 1.82 | 42.7 | 5.58 | |
| Path density | 0.06 | 2.06 | 0 | 0.17 | 0.57 | 0.48 | 3.68 | 1.31 | 0.02 | 3.24 | 0.19 | 3.34 | 0.27 | |
| Path density^2 | 0.06 | 2.75 | 0 | 0.18 | 0.6 | 0.66 | 6.25 | 1.94 | 0.02 | 3.97 | 0.2 | 4.61 | 0.33 | |
| Population | 0.27 | 1.07 | 0.09 | 0.43 | 0.65 | 0.21 | 0.73 | 0.13 | 0.27 | 3.99 | 0.34 | 1.7 | 0.46 | |
| Population^2 | 0.44 | 2.1 | 0.09 | 0.77 | 1.2 | 0.44 | 1.58 | 0.23 | 0.45 | 6.52 | 0.59 | 3.46 | 0.91 | |
| Log population | 0.6 | 20.01 | 0.03 | 2.65 | 7.08 | 6.4 | 34.62 | 12.02 | 0.78 | 30.64 | 1.51 | 35.2 | 4.17 | |
| Log population^2 | 0.81 | 20.18 | 0.05 | 2.95 | 7.57 | 6.65 | 40.19 | 14.86 | 1.01 | 30.63 | 1.58 | 36.49 | 4.31 | |

(b) O_0.5_ = 2.

|  | **R^2^** | | | | | | | | | | | | |  |
| --- | --- | --- | --- | --- | --- | --- | --- | --- | --- | --- | --- | --- | --- | --- |
|  | Araneae | Coleaoptera | Opilindae | Odonata | Papilionoidea | Amphibian | Birds | Land mammals | Bryophyta | Fungi | Lichen | Tracheophyta | Poacea | |
| Elevation | 0.31 | 14.17 | 0.01 | 1.78 | 4.23 | 6.46 | 37.57 | 13.64 | 0.42 | 48.32 | 0.76 | 37.04 | 3.9 | |
| Elevation^2 | 0.45 | 14.26 | 0.02 | 2.21 | 5.72 | 6.47 | 41.83 | 15.76 | 0.61 | 49.79 | 0.85 | 37.36 | 4.08 | |
| Slope | 0.1 | 4.34 | 0 | 0.52 | 0.9 | 3.93 | 25.28 | 7.67 | 0.13 | 28.53 | 0.32 | 22.18 | 2.83 | |
| Slope^2 | 0.13 | 4.35 | 0.01 | 0.6 | 1.13 | 4.02 | 25.62 | 7.98 | 0.18 | 29.36 | 0.35 | 22.29 | 3.22 | |
| Road density | 0.59 | 16.92 | 0.02 | 2.86 | 7.19 | 8.1 | 29.05 | 13.7 | 0.72 | 52.54 | 1.74 | 37.12 | 5.53 | |
| Road density^2 | 0.61 | 20.24 | 0.03 | 2.85 | 7.44 | 9.18 | 42.06 | 18.62 | 0.74 | 57.42 | 1.75 | 44.47 | 5.74 | |
| Path density | 0.05 | 1.87 | 0 | 0.16 | 0.57 | 0.47 | 3.4 | 1.35 | 0.02 | 8.97 | 0.19 | 3.65 | 0.27 | |
| Path density^2 | 0.06 | 2.43 | 0 | 0.17 | 0.59 | 0.64 | 5.66 | 1.95 | 0.02 | 10.7 | 0.19 | 4.93 | 0.34 | |
| Population | 0.26 | 1.12 | 0.08 | 0.47 | 0.74 | 0.23 | 0.73 | 0.14 | 0.28 | 11.73 | 0.37 | 2.07 | 0.53 | |
| Population^2 | 0.43 | 2.1 | 0.08 | 0.82 | 1.34 | 0.45 | 1.56 | 0.24 | 0.44 | 17.86 | 0.62 | 4.08 | 1.02 | |
| Log population | 0.56 | 18.39 | 0.02 | 2.55 | 7.17 | 6.24 | 31.38 | 11.72 | 0.72 | 57.11 | 1.45 | 36.74 | 4.26 | |
| Log population^2 | 0.76 | 18.51 | 0.04 | 2.87 | 7.75 | 6.46 | 36.48 | 14.53 | 0.95 | 57.15 | 1.52 | 37.63 | 4.45 | |

(c) O_0.5_ = 5.

|  | **R^2^** | | | | | | | | | | | | |  |
| --- | --- | --- | --- | --- | --- | --- | --- | --- | --- | --- | --- | --- | --- | --- |
|  | Araneae | Coleaoptera | Opilindae | Odonata | Papilionoidea | Amphibian | Birds | Land mammals | Bryophyta | Fungi | Lichen | Tracheophyta | Poacea | |
| Elevation | 0.28 | 10.33 | 0.01 | 1.61 | 3.85 | 5.34 | 24.15 | 10.34 | 0.38 | 69.43 | 0.68 | 22.93 | 3.35 | |
| Elevation^2 | 0.4 | 10.39 | 0.02 | 1.98 | 5.21 | 5.35 | 27.88 | 12.2 | 0.55 | 71.48 | 0.76 | 23.13 | 3.52 | |
| Slope | 0.09 | 2.99 | 0 | 0.46 | 0.8 | 3.23 | 15.15 | 5.69 | 0.12 | 47.5 | 0.29 | 12.49 | 2.43 | |
| Slope^2 | 0.11 | 3.01 | 0 | 0.53 | 1.01 | 3.28 | 15.58 | 6.04 | 0.16 | 49.02 | 0.31 | 12.53 | 2.75 | |
| Road density | 0.53 | 12.72 | 0.02 | 2.64 | 6.76 | 7 | 18.45 | 10.92 | 0.65 | 74.93 | 1.58 | 24.36 | 4.97 | |
| Road density^2 | 0.55 | 15.31 | 0.02 | 2.64 | 6.94 | 7.91 | 28.51 | 14.84 | 0.67 | 77.54 | 1.58 | 29.83 | 5.13 | |
| Path density | 0.05 | 1.34 | 0 | 0.15 | 0.51 | 0.4 | 1.99 | 1.07 | 0.02 | 20.09 | 0.17 | 2.02 | 0.24 | |
| Path density^2 | 0.05 | 1.71 | 0 | 0.15 | 0.53 | 0.54 | 3.27 | 1.5 | 0.02 | 22.86 | 0.17 | 2.68 | 0.29 | |
| Population | 0.23 | 0.89 | 0.07 | 0.49 | 0.82 | 0.2 | 0.44 | 0.12 | 0.28 | 28.76 | 0.38 | 1.28 | 0.53 | |
| Population^2 | 0.39 | 1.63 | 0.08 | 0.81 | 1.42 | 0.38 | 0.97 | 0.2 | 0.41 | 39.08 | 0.6 | 2.48 | 0.99 | |
| Log population | 0.5 | 13.81 | 0.02 | 2.33 | 6.68 | 5.19 | 19.8 | 8.89 | 0.65 | 77.85 | 1.3 | 23.47 | 3.77 | |
| Log population^2 | 0.69 | 13.95 | 0.04 | 2.64 | 7.28 | 5.41 | 23.96 | 11.35 | 0.86 | 78.15 | 1.37 | 24.03 | 3.98 | |

(d) O_0.5_ = 10.

|  | **R^2^** | | | | | | | | | | | | |  |
| --- | --- | --- | --- | --- | --- | --- | --- | --- | --- | --- | --- | --- | --- | --- |
|  | Araneae | Coleaoptera | Opilindae | Odonata | Papilionoidea | Amphibian | Birds | Land mammals | Bryophyta | Fungi | Lichen | Tracheophyta | Poacea | |
| Elevation | 0.26 | 7.95 | 0.01 | 1.46 | 3.44 | 4.41 | 16.74 | 7.87 | 0.35 | 51.58 | 0.61 | 14.93 | 2.81 | |
| Elevation^2 | 0.37 | 7.99 | 0.02 | 1.79 | 4.63 | 4.42 | 19.85 | 9.46 | 0.5 | 54.75 | 0.68 | 15.08 | 2.96 | |
| Slope | 0.08 | 2.23 | 0 | 0.42 | 0.71 | 2.66 | 10.14 | 4.28 | 0.11 | 29.05 | 0.26 | 7.75 | 2.04 | |
| Slope^2 | 0.11 | 2.25 | 0 | 0.48 | 0.89 | 2.69 | 10.57 | 4.61 | 0.15 | 30.58 | 0.28 | 7.75 | 2.29 | |
| Road density | 0.49 | 9.82 | 0.02 | 2.43 | 6.09 | 5.83 | 12.48 | 8.38 | 0.6 | 60.14 | 1.43 | 16.18 | 4.22 | |
| Road density^2 | 0.51 | 12.01 | 0.02 | 2.43 | 6.24 | 6.68 | 20.4 | 11.64 | 0.61 | 62.63 | 1.43 | 20.41 | 4.39 | |
| Path density | 0.05 | 1.01 | 0 | 0.13 | 0.45 | 0.33 | 1.31 | 0.82 | 0.02 | 10.96 | 0.15 | 1.24 | 0.2 | |
| Path density^2 | 0.05 | 1.28 | 0 | 0.14 | 0.47 | 0.44 | 2.15 | 1.15 | 0.02 | 12.38 | 0.15 | 1.63 | 0.24 | |
| Population | 0.18 | 0.61 | 0.06 | 0.46 | 0.82 | 0.16 | 0.27 | 0.09 | 0.27 | 19.25 | 0.36 | 0.77 | 0.47 | |
| Population^2 | 0.35 | 1.23 | 0.07 | 0.75 | 1.38 | 0.32 | 0.64 | 0.15 | 0.38 | 27.09 | 0.56 | 1.55 | 0.86 | |
| Log population | 0.47 | 10.76 | 0.02 | 2.14 | 6.04 | 4.28 | 13.5 | 6.72 | 0.6 | 63.87 | 1.18 | 15.54 | 3.22 | |
| Log population^2 | 0.64 | 10.92 | 0.04 | 2.41 | 6.54 | 4.51 | 16.9 | 8.83 | 0.78 | 64.79 | 1.24 | 15.99 | 3.39 | |

(e) O_0.5_ = 20.

|  | **R^2^** | | | | | | | | | | | | |  |
| --- | --- | --- | --- | --- | --- | --- | --- | --- | --- | --- | --- | --- | --- | --- |
|  | Araneae | Coleaoptera | Opilindae | Odonata | Papilionoidea | Amphibian | Birds | Land mammals | Bryophyta | Fungi | Lichen | Tracheophyta | Poacea | |
| Elevation | 0.24 | 6.26 | 0.01 | 1.32 | 3.03 | 3.64 | 12.12 | 6.04 | 0.32 | 30.86 | 0.55 | 10.21 | 2.33 | |
| Elevation^2 | 0.35 | 6.29 | 0.01 | 1.62 | 4.05 | 3.66 | 14.65 | 7.38 | 0.46 | 34.13 | 0.61 | 10.33 | 2.46 | |
| Slope | 0.08 | 1.72 | 0 | 0.38 | 0.62 | 2.19 | 7.19 | 3.25 | 0.1 | 14.4 | 0.23 | 5.16 | 1.69 | |
| Slope^2 | 0.1 | 1.74 | 0 | 0.43 | 0.78 | 2.2 | 7.58 | 3.56 | 0.13 | 15.4 | 0.25 | 5.16 | 1.89 | |
| Road density | 0.46 | 7.71 | 0.02 | 2.21 | 5.34 | 4.81 | 8.83 | 6.4 | 0.55 | 40.04 | 1.29 | 11.07 | 3.5 | |
| Road density^2 | 0.47 | 9.58 | 0.02 | 2.22 | 5.49 | 5.59 | 15.07 | 9.12 | 0.56 | 41.87 | 1.29 | 14.41 | 3.68 | |
| Path density | 0.04 | 0.78 | 0 | 0.12 | 0.39 | 0.27 | 0.92 | 0.63 | 0.01 | 5.08 | 0.13 | 0.82 | 0.16 | |
| Path density^2 | 0.04 | 0.99 | 0 | 0.12 | 0.41 | 0.36 | 1.51 | 0.88 | 0.02 | 5.69 | 0.14 | 1.07 | 0.2 | |
| Population | 0.13 | 0.36 | 0.06 | 0.39 | 0.75 | 0.12 | 0.18 | 0.07 | 0.24 | 11.46 | 0.3 | 0.44 | 0.35 | |
| Population^2 | 0.32 | 0.95 | 0.07 | 0.66 | 1.23 | 0.26 | 0.45 | 0.12 | 0.35 | 16.49 | 0.49 | 1.02 | 0.7 | |
| Log population | 0.44 | 8.53 | 0.02 | 1.94 | 5.33 | 3.51 | 9.65 | 5.11 | 0.55 | 44.12 | 1.06 | 10.72 | 2.69 | |
| Log population^2 | 0.59 | 8.7 | 0.03 | 2.18 | 5.69 | 3.74 | 12.4 | 6.89 | 0.72 | 45.72 | 1.12 | 11.1 | 2.83 | |

(f) O_0.5_ = 50.

|  | **R^2^** | | | | | | | | | | | | |  |
| --- | --- | --- | --- | --- | --- | --- | --- | --- | --- | --- | --- | --- | --- | --- |
|  | Araneae | Coleaoptera | Opilindae | Odonata | Papilionoidea | Amphibian | Birds | Land mammals | Bryophyta | Fungi | Lichen | Tracheophyta | Poacea | |
| Elevation | 0.22 | 4.79 | 0.01 | 1.17 | 2.56 | 2.89 | 8.58 | 4.47 | 0.29 | 15.48 | 0.48 | 6.81 | 1.85 | |
| Elevation^2 | 0.32 | 4.8 | 0.01 | 1.42 | 3.39 | 2.91 | 10.53 | 5.53 | 0.41 | 17.74 | 0.54 | 6.91 | 1.95 | |
| Slope | 0.07 | 1.3 | 0 | 0.33 | 0.52 | 1.73 | 5 | 2.39 | 0.09 | 6.43 | 0.2 | 3.38 | 1.34 | |
| Slope^2 | 0.09 | 1.32 | 0 | 0.38 | 0.65 | 1.74 | 5.32 | 2.65 | 0.12 | 6.91 | 0.22 | 3.38 | 1.49 | |
| Road density | 0.42 | 5.86 | 0.02 | 1.96 | 4.47 | 3.8 | 6.12 | 4.69 | 0.49 | 22.15 | 1.13 | 7.33 | 2.76 | |
| Road density^2 | 0.43 | 7.4 | 0.02 | 1.97 | 4.62 | 4.49 | 10.83 | 6.85 | 0.5 | 23.11 | 1.13 | 9.84 | 2.94 | |
| Path density | 0.04 | 0.59 | 0 | 0.1 | 0.33 | 0.21 | 0.63 | 0.46 | 0.01 | 2.22 | 0.12 | 0.53 | 0.13 | |
| Path density^2 | 0.04 | 0.75 | 0 | 0.11 | 0.34 | 0.29 | 1.04 | 0.65 | 0.01 | 2.46 | 0.12 | 0.7 | 0.16 | |
| Population | 0.11 | 0.25 | 0.05 | 0.23 | 0.53 | 0.09 | 0.12 | 0.05 | 0.2 | 6.83 | 0.2 | 0.26 | 0.21 | |
| Population^2 | 0.29 | 0.71 | 0.06 | 0.56 | 0.97 | 0.2 | 0.31 | 0.08 | 0.31 | 9.75 | 0.42 | 0.66 | 0.54 | |
| Log population | 0.4 | 6.55 | 0.02 | 1.72 | 4.5 | 2.78 | 6.75 | 3.75 | 0.49 | 25.61 | 0.93 | 7.18 | 2.15 | |
| Log population^2 | 0.54 | 6.71 | 0.03 | 1.91 | 4.7 | 2.98 | 8.87 | 5.17 | 0.64 | 27.37 | 0.98 | 7.49 | 2.24 | |

**S1 File Table B**

**Table B**. **Detailed model results for all 12 reference taxonomic groups**. Deviance and mode of parameter estimates for each model for all levels of O_0.5_.

**O_0.5_ = 1**

1. Araneae

|  | Dev | DeltaDev | phi | Intercept | X | X^2^ |
| --- | --- | --- | --- | --- | --- | --- |
| Null | -67807 | 0,00 | 0,83 | 2,081 | - | - |
| elev | -68036 | 229,14 | 0,86 | 1,787 | 0,001 | - |
| slope | -67880 | 72,94 | 0,84 | 1,889 | 0,057 | - |
| road | -68237 | 430,50 | 0,90 | 2,565 | -2,252 | - |
| path | -67847 | 40,72 | 0,84 | 2,187 | -0,849 | - |
| popn | -67989 | 181,88 | 0,88 | 2,169 | -0,003 | - |
| logpopn | -68215 | 407,81 | 0,90 | 2,172 | -0,404 | - |
| elevSq | -68137 | 101,58 | 0,87 | 1,578 | 0,003 | 0,000 |
| slopeSq | -67901 | 20,96 | 0,84 | 1,777 | 0,119 | -0,005 |
| roadSq | -68255 | 17,46 | 0,91 | 2,467 | -0,969 | -2,232 |
| pathSq | -67848 | 0,62 | 0,84 | 2,229 | -1,374 | 1,112 |
| popnSq | -68110 | 120,93 | 0,91 | 2,213 | -0,005 | 0,000 |
| logpopnSq | -68362 | 147,39 | 0,94 | 2,345 | -0,390 | -0,202 |

1. Coleaoptera

|  | Dev | DeltaDev | phi | Intercept | X | X^2^ |
| --- | --- | --- | --- | --- | --- | --- |
| Null | -91372 | 0,00 | 2,12 | 3,201 | - | - |
| elev | -91391 | 18,97 | 2,11 | 3,114 | 0,000 | - |
| slope | -91379 | 7,66 | 2,14 | 3,147 | 0,017 | - |
| road | -91391 | 19,21 | 2,14 | 3,285 | -0,415 | - |
| path | -91375 | 3,40 | 2,11 | 3,233 | -0,272 | - |
| popn | -91390 | 18,22 | 2,10 | 3,211 | 0,000 | - |
| logpopn | -91402 | 30,85 | 2,11 | 3,219 | -0,099 | - |
| elevSq | -91402 | 11,12 | 2,12 | 3,047 | 0,001 | 0,000 |
| slopeSq | -91383 | 3,34 | 2,11 | 3,095 | 0,048 | -0,002 |
| roadSq | -91390 | -0,74 | 2,13 | 3,276 | -0,476 | 0,176 |
| pathSq | -91374 | -0,56 | 2,10 | 3,232 | -0,386 | 0,317 |
| popnSq | -91395 | 5,26 | 2,14 | 3,228 | -0,001 | 0,000 |
| logpopnSq | -91414 | 11,89 | 2,12 | 3,252 | -0,091 | -0,052 |

1. Opilionidae

|  | Dev | DeltaDev | phi | Intercept | X | X^2^ |
| --- | --- | --- | --- | --- | --- | --- |
| Null | -92934 | 0,00 | 2,58 | 3,395 | - | - |
| elev | -92946 | 11,86 | 2,58 | 3,330 | 0,000 | - |
| slope | -92938 | 4,13 | 2,56 | 3,339 | 0,014 | - |
| road | -92958 | 23,80 | 2,58 | 3,490 | -0,449 | - |
| path | -92934 | 0,36 | 2,58 | 3,414 | -0,092 | - |
| popn | -93013 | 79,51 | 2,63 | 3,449 | -0,001 | - |
| logpopn | -92959 | 25,62 | 2,58 | 3,421 | -0,087 | - |
| elevSq | -92953 | 6,92 | 2,58 | 3,270 | 0,001 | 0,000 |
| slopeSq | -92940 | 1,94 | 2,57 | 3,320 | 0,027 | -0,001 |
| roadSq | -92960 | 2,02 | 2,56 | 3,455 | -0,009 | -0,760 |
| pathSq | -92933 | -0,89 | 2,57 | 3,426 | -0,208 | 0,126 |
| popnSq | -93018 | 5,17 | 2,67 | 3,455 | -0,001 | 0,000 |
| logpopnSq | -92979 | 19,95 | 2,58 | 3,475 | -0,079 | -0,067 |

1. Odonata

|  | Dev | DeltaDev | phi | Intercept | X | X^2^ |
| --- | --- | --- | --- | --- | --- | --- |
| Null | -42303 | 0,00 | 0,60 | 1,401 | - | - |
| elev | -43113 | 809,68 | 0,69 | 0,797 | 0,002 | - |
| slope | -42536 | 232,38 | 0,63 | 1,038 | 0,105 | - |
| road | -43579 | 1275,54 | 0,79 | 2,336 | -4,204 | - |
| path | -42376 | 72,32 | 0,61 | 1,563 | -1,247 | - |
| popn | -42487 | 183,30 | 0,63 | 1,485 | -0,003 | - |
| logpopn | -43454 | 1150,22 | 0,75 | 1,580 | -0,735 | - |
| elevSq | -43310 | 197,03 | 0,72 | 0,499 | 0,004 | 0,000 |
| slopeSq | -42573 | 37,46 | 0,63 | 0,876 | 0,190 | -0,007 |
| roadSq | -43581 | 2,49 | 0,78 | 2,378 | -4,694 | 0,875 |
| pathSq | -42379 | 3,12 | 0,61 | 1,595 | -1,860 | 1,439 |
| popnSq | -42632 | 145,35 | 0,65 | 1,541 | -0,006 | 0,000 |
| logpopnSq | -43587 | 133,46 | 0,78 | 1,761 | -0,710 | -0,212 |

1. Papilionoidea

|  | Dev | DeltaDev | phi | Intercept | X | X^2^ |
| --- | --- | --- | --- | --- | --- | --- |
| Null | -27099 | 0,00 | 0,55 | 0,982 | - | - |
| elev | -28309 | 1210,00 | 0,71 | 0,201 | 0,003 | - |
| slope | -27350 | 251,53 | 0,58 | 0,593 | 0,117 | - |
| road | -29138 | 2039,76 | 0,88 | 2,286 | -5,806 | - |
| path | -27255 | 155,99 | 0,57 | 1,246 | -1,977 | - |
| popn | -27275 | 176,55 | 0,59 | 1,071 | -0,003 | - |
| logpopn | -29164 | 2065,91 | 0,87 | 1,263 | -1,084 | - |
| elevSq | -28753 | 444,33 | 0,78 | -0,288 | 0,007 | 0,000 |
| slopeSq | -27415 | 65,15 | 0,59 | 0,357 | 0,235 | -0,009 |
| roadSq | -29239 | 100,23 | 0,89 | 2,531 | -8,916 | 5,591 |
| pathSq | -27262 | 7,26 | 0,58 | 1,294 | -2,913 | 2,412 |
| popnSq | -27426 | 151,34 | 0,61 | 1,117 | -0,006 | 0,000 |
| logpopnSq | -29319 | 154,34 | 0,91 | 1,452 | -1,060 | -0,254 |

1. Bryophyta

|  | Dev | DeltaDev | phi | Intercept | X | X^2^ |
| --- | --- | --- | --- | --- | --- | --- |
| Null | -14195 | 0,00 | 0,70 | 0,790 | - | - |
| elev | -15219 | 1024,14 | 0,84 | 0,130 | 0,002 | - |
| slope | -14803 | 608,09 | 0,78 | 0,199 | 0,173 | - |
| road | -15424 | 1228,80 | 0,90 | 1,689 | -4,181 | - |
| path | -14263 | 68,33 | 0,72 | 0,956 | -1,277 | - |
| popn | -14225 | 30,57 | 0,71 | 0,811 | -0,001 | - |
| logpopn | -15166 | 971,32 | 0,84 | 0,907 | -0,647 | - |
| elevSq | -15220 | 1,26 | 0,84 | 0,165 | 0,002 | 0,000 |
| slopeSq | -14821 | 17,95 | 0,79 | 0,066 | 0,242 | -0,006 |
| roadSq | -15633 | 209,10 | 0,93 | 2,007 | -8,376 | 7,760 |
| pathSq | -14289 | 25,81 | 0,72 | 1,059 | -3,036 | 3,907 |
| popnSq | -14257 | 31,58 | 0,71 | 0,833 | -0,002 | 0,000 |
| logpopnSq | -15206 | 39,35 | 0,84 | 0,823 | -0,651 | 0,111 |

1. Fungi

|  | Dev | DeltaDev | phi | Intercept | X | X^2^ |
| --- | --- | --- | --- | --- | --- | --- |
| Null | -3037 | 0,00 | 1,04 | 0,346 | - | - |
| elev | -5224 | 2186,54 | 1,56 | -0,632 | 0,003 | - |
| slope | -4277 | 1239,57 | 1,30 | -0,479 | 0,228 | - |
| road | -4410 | 1373,17 | 1,38 | 1,219 | -4,312 | - |
| path | -3153 | 115,92 | 1,06 | 0,566 | -1,638 | - |
| popn | -3060 | 22,42 | 1,04 | 0,371 | -0,001 | - |
| logpopn | -4645 | 1608,21 | 1,42 | 0,447 | -0,789 | - |
| elevSq | -5647 | 423,26 | 1,69 | -0,147 | -0,001 | 0,000 |
| slopeSq | -4293 | 16,09 | 1,30 | -0,327 | 0,147 | 0,007 |
| roadSq | -5568 | 1157,59 | 1,71 | 1,956 | -14,382 | 18,948 |
| pathSq | -3240 | 86,73 | 1,08 | 0,731 | -4,785 | 7,127 |
| popnSq | -3086 | 26,36 | 1,05 | 0,388 | -0,002 | 0,000 |
| logpopnSq | -5078 | 432,67 | 1,53 | 0,179 | -0,814 | 0,345 |

1. Lichen

|  | Dev | DeltaDev | phi | Intercept | X | X^2^ |
| --- | --- | --- | --- | --- | --- | --- |
| Null | -8008 | 0,00 | 0,78 | 0,535 | - | - |
| elev | -9336 | 1327,74 | 0,98 | -0,232 | 0,002 | - |
| slope | -8713 | 705,01 | 0,88 | -0,081 | 0,173 | - |
| road | -9242 | 1233,47 | 1,00 | 1,391 | -4,202 | - |
| path | -8115 | 106,67 | 0,79 | 0,733 | -1,583 | - |
| popn | -8019 | 10,31 | 0,79 | 0,539 | 0,000 | - |
| logpopn | -9102 | 1093,84 | 0,95 | 0,626 | -0,669 | - |
| elevSq | -9575 | 238,78 | 1,02 | 0,165 | -0,001 | 0,000 |
| slopeSq | -8737 | 23,20 | 0,88 | 0,075 | 0,086 | 0,008 |
| roadSq | -9851 | 608,83 | 1,11 | 1,933 | -11,358 | 13,771 |
| pathSq | -8167 | 51,62 | 0,80 | 0,882 | -3,987 | 5,579 |
| popnSq | -8027 | 8,02 | 0,78 | 0,554 | -0,001 | 0,000 |
| logpopnSq | -9407 | 304,27 | 1,00 | 0,396 | -0,700 | 0,306 |

1. Amphibia

|  | Dev | DeltaDev | phi | Intercept | X | X^2^ |
| --- | --- | --- | --- | --- | --- | --- |
| Null | -66062 | 0,00 | 0,62 | 1,768 | - | - |
| elev | -66366 | 304,66 | 0,65 | 1,419 | 0,001 | - |
| slope | -66156 | 94,54 | 0,63 | 1,560 | 0,066 | - |
| road | -66581 | 518,70 | 0,69 | 2,336 | -2,566 | - |
| path | -66076 | 14,17 | 0,62 | 1,850 | -0,559 | - |
| popn | -66244 | 181,70 | 0,66 | 1,868 | -0,003 | - |
| logpopn | -66578 | 516,40 | 0,68 | 1,890 | -0,472 | - |
| elevSq | -66501 | 134,59 | 0,68 | 1,176 | 0,003 | 0,000 |
| slopeSq | -66190 | 33,18 | 0,64 | 1,397 | 0,143 | -0,006 |
| roadSq | -66590 | 9,55 | 0,70 | 2,257 | -1,598 | -1,739 |
| pathSq | -66078 | 2,24 | 0,62 | 1,876 | -1,042 | 1,088 |
| popnSq | -66358 | 114,46 | 0,67 | 1,911 | -0,005 | 0,000 |
| logpopnSq | -66739 | 160,58 | 0,72 | 2,070 | -0,458 | -0,215 |

1. Aves

|  | Dev | DeltaDev | phi | Intercept | X | X^2^ |
| --- | --- | --- | --- | --- | --- | --- |
| Null | -3649 | 0,00 | 1,14 | -0,557 | - | - |
| elev | -4830 | 1181,05 | 1,48 | -1,351 | 0,002 | - |
| slope | -4182 | 533,20 | 1,29 | -1,169 | 0,175 | - |
| road | -4960 | 1311,12 | 1,52 | 0,278 | -4,178 | - |
| path | -3771 | 122,16 | 1,17 | -0,350 | -1,664 | - |
| popn | -3801 | 151,76 | 1,18 | -0,510 | -0,002 | - |
| logpopn | -5260 | 1611,63 | 1,62 | -0,441 | -0,839 | - |
| elevSq | -4875 | 44,87 | 1,50 | -1,502 | 0,004 | 0,000 |
| slopeSq | -4196 | 13,94 | 1,30 | -1,280 | 0,237 | -0,005 |
| roadSq | -5291 | 330,97 | 1,65 | 0,701 | -9,300 | 8,915 |
| pathSq | -3800 | 28,66 | 1,19 | -0,235 | -3,364 | 3,750 |
| popnSq | -3903 | 102,86 | 1,20 | -0,482 | -0,004 | 0,000 |
| logpopnSq | -5260 | -0,58 | 1,63 | -0,448 | -0,847 | 0,012 |

1. Land mammals

|  | Dev | DeltaDev | phi | Intercept | X | X^2^ |
| --- | --- | --- | --- | --- | --- | --- |
| Null | -37771 | 0,00 | 0,63 | 1,358 | - | - |
| elev | -38077 | 306,58 | 0,66 | 1,002 | 0,001 | - |
| slope | -37900 | 129,30 | 0,64 | 1,084 | 0,077 | - |
| road | -38466 | 695,17 | 0,72 | 2,003 | -2,986 | - |
| path | -37844 | 73,77 | 0,64 | 1,518 | -1,306 | - |
| popn | -37899 | 128,60 | 0,65 | 1,416 | -0,002 | - |
| logpopn | -38351 | 580,47 | 0,69 | 1,463 | -0,490 | - |
| elevSq | -38114 | 36,37 | 0,66 | 0,870 | 0,002 | 0,000 |
| slopeSq | -37912 | 12,00 | 0,64 | 0,982 | 0,129 | -0,004 |
| roadSq | -38471 | 4,99 | 0,71 | 2,062 | -3,637 | 1,314 |
| pathSq | -37847 | 2,22 | 0,64 | 1,552 | -1,719 | 1,284 |
| popnSq | -37993 | 94,20 | 0,67 | 1,457 | -0,004 | 0,000 |
| logpopnSq | -38376 | 25,19 | 0,70 | 1,531 | -0,485 | -0,089 |

1. Vascular plants

|  | Dev | DeltaDev | phi | Intercept | X | X^2^ |
| --- | --- | --- | --- | --- | --- | --- |
| Null | -2216 | 0,00 | 1,05 | 0,174 | - | - |
| elev | -3486 | 1270,39 | 1,36 | -0,608 | 0,002 | - |
| slope | -2841 | 625,52 | 1,19 | -0,446 | 0,177 | - |
| road | -3383 | 1167,23 | 1,35 | 0,992 | -4,007 | - |
| path | -2292 | 76,53 | 1,06 | 0,345 | -1,326 | - |
| popn | -2254 | 38,35 | 1,06 | 0,198 | -0,001 | - |
| logpopn | -3419 | 1203,30 | 1,35 | 0,288 | -0,722 | - |
| elevSq | -3512 | 25,98 | 1,37 | -0,495 | 0,001 | 0,000 |
| slopeSq | -2845 | 3,59 | 1,19 | -0,513 | 0,206 | -0,003 |
| roadSq | -3866 | 483,57 | 1,49 | 1,520 | -10,514 | 11,758 |
| pathSq | -2323 | 30,61 | 1,07 | 0,451 | -3,197 | 4,135 |
| popnSq | -2295 | 41,04 | 1,06 | 0,216 | -0,002 | 0,000 |
| logpopnSq | -3489 | 69,61 | 1,35 | 0,176 | -0,735 | 0,137 |

1. Poacea

|  | Dev | DeltaDev | phi | Intercept | X | X^2^ |
| --- | --- | --- | --- | --- | --- | --- |
| Null | -15322 | 0,00 | 0,65 | 0,752 | - | - |
| elev | -15951 | 629,06 | 0,73 | 0,211 | 0,002 | - |
| slope | -15774 | 451,67 | 0,70 | 0,208 | 0,158 | - |
| road | -16181 | 859,00 | 0,78 | 1,516 | -3,542 | - |
| path | -15363 | 41,48 | 0,65 | 0,875 | -1,021 | - |
| popn | -15393 | 71,17 | 0,66 | 0,785 | -0,001 | - |
| logpopn | -15990 | 667,55 | 0,74 | 0,863 | -0,561 | - |
| elevSq | -15975 | 24,37 | 0,73 | 0,085 | 0,003 | 0,000 |
| slopeSq | -15838 | 64,34 | 0,71 | -0,023 | 0,278 | -0,010 |
| roadSq | -16227 | 45,94 | 0,78 | 1,677 | -5,559 | 3,753 |
| pathSq | -15373 | 10,02 | 0,65 | 0,939 | -2,096 | 2,628 |
| popnSq | -15462 | 68,79 | 0,67 | 0,823 | -0,003 | 0,000 |
| logpopnSq | -16012 | 22,72 | 0,75 | 0,932 | -0,557 | -0,086 |

**O_0.5_ = 2**

1. Araneae

|  | Dev | DeltaDev | phi | Intercept | X | X^2^ |
| --- | --- | --- | --- | --- | --- | --- |
| Null | -73020 | 0.00 | 1.13 | 2.429 | - | - |
| elev | -73249 | 228.71 | 1.19 | 2.146 | 0.001 | - |
| slope | -73093 | 72.68 | 1.15 | 2.241 | 0.055 | - |
| road | -73451 | 430.20 | 1.23 | 2.912 | -2.199 | - |
| path | -73060 | 40.16 | 1.15 | 2.544 | -0.829 | - |
| popn | -73213 | 192.84 | 1.22 | 2.527 | -0.003 | - |
| logpopn | -73429 | 408.87 | 1.23 | 2.530 | -0.400 | - |
| elevSq | -73349 | 100.09 | 1.20 | 1.954 | 0.003 | 0.000 |
| slopeSq | -73114 | 20.60 | 1.15 | 2.131 | 0.113 | -0.005 |
| roadSq | -73469 | 18.42 | 1.24 | 2.818 | -0.957 | -2.204 |
| pathSq | -73062 | 1.18 | 1.14 | 2.562 | -1.223 | 0.963 |
| popnSq | -73335 | 121.71 | 1.24 | 2.573 | -0.005 | 0.000 |
| logpopnSq | -73580 | 150.48 | 1.28 | 2.693 | -0.375 | -0.192 |

1. Coleaoptera

|  | Dev | DeltaDev | phi | Intercept | X | X^2^ |
| --- | --- | --- | --- | --- | --- | --- |
| Null | -9187 | 0.00 | 1.22 | 1.060 | - | - |
| elev | -10705 | 1517.10 | 1.63 | 0.322 | 0.002 | - |
| slope | -9604 | 416.85 | 1.31 | 0.635 | 0.126 | - |
| road | -11059 | 1871.74 | 1.80 | 2.088 | -4.731 | - |
| path | -9362 | 174.80 | 1.27 | 1.308 | -1.864 | - |
| popn | -9291 | 103.65 | 1.26 | 1.111 | -0.002 | - |
| logpopn | -11258 | 2070.67 | 1.84 | 1.274 | -0.899 | - |
| elevSq | -10716 | 11.00 | 1.63 | 0.254 | 0.003 | 0.000 |
| slopeSq | -9606 | 1.21 | 1.32 | 0.666 | 0.105 | 0.002 |
| roadSq | -11519 | 460.21 | 1.94 | 2.575 | -10.607 | 10.867 |
| pathSq | -9417 | 54.26 | 1.28 | 1.438 | -4.211 | 5.416 |
| popnSq | -9385 | 93.84 | 1.28 | 1.145 | -0.004 | 0.000 |
| logpopnSq | -11275 | 16.77 | 1.85 | 1.220 | -0.920 | 0.074 |

1. Opilionidae

|  | Dev | DeltaDev | phi | Intercept | X | X^2^ |
| --- | --- | --- | --- | --- | --- | --- |
| Null | -99245 | 0.00 | 3.88 | 3.836 | - | - |
| elev | -99257 | 11.34 | 3.85 | 3.764 | 0.000 | - |
| slope | -99249 | 3.85 | 3.89 | 3.783 | 0.013 | - |
| road | -99268 | 23.01 | 3.93 | 3.934 | -0.450 | - |
| path | -99246 | 0.34 | 3.86 | 3.844 | -0.118 | - |
| popn | -99322 | 76.32 | 3.97 | 3.868 | -0.001 | - |
| logpopn | -99270 | 24.63 | 3.94 | 3.854 | -0.088 | - |
| elevSq | -99263 | 6.73 | 3.87 | 3.709 | 0.001 | 0.000 |
| slopeSq | -99251 | 1.65 | 3.93 | 3.741 | 0.030 | -0.001 |
| roadSq | -99270 | 1.81 | 3.90 | 3.877 | -0.009 | -0.794 |
| pathSq | -99245 | -0.99 | 3.87 | 3.849 | -0.176 | -0.012 |
| popnSq | -99328 | 6.21 | 4.01 | 3.884 | -0.002 | 0.000 |
| logpopnSq | -99289 | 19.08 | 3.89 | 3.905 | -0.080 | -0.071 |

1. Odonata

|  | Dev | DeltaDev | phi | Intercept | X | X^2^ |
| --- | --- | --- | --- | --- | --- | --- |
| Null | -45829 | 0.00 | 0.76 | 1.692 | - | - |
| elev | -46662 | 832.53 | 0.89 | 1.127 | 0.002 | - |
| slope | -46067 | 237.55 | 0.80 | 1.352 | 0.104 | - |
| road | -47176 | 1346.97 | 1.02 | 2.647 | -4.192 | - |
| path | -45904 | 74.43 | 0.77 | 1.858 | -1.234 | - |
| popn | -46045 | 215.84 | 0.83 | 1.796 | -0.003 | - |
| logpopn | -47030 | 1200.32 | 0.97 | 1.891 | -0.733 | - |
| elevSq | -46864 | 202.02 | 0.92 | 0.810 | 0.005 | 0.000 |
| slopeSq | -46105 | 38.16 | 0.79 | 1.191 | 0.184 | -0.007 |
| roadSq | -47176 | -0.49 | 1.01 | 2.648 | -4.295 | 0.288 |
| pathSq | -45907 | 3.20 | 0.78 | 1.889 | -1.753 | 1.170 |
| popnSq | -46206 | 160.96 | 0.85 | 1.849 | -0.006 | 0.000 |
| logpopnSq | -47183 | 153.79 | 1.01 | 2.073 | -0.704 | -0.217 |

1. Papilionoidea

|  | Dev | DeltaDev | phi | Intercept | X | X^2^ |
| --- | --- | --- | --- | --- | --- | --- |
| Null | -29269 | 0.00 | 0.68 | 1.254 | - | - |
| elev | -30560 | 1291.45 | 0.88 | 0.482 | 0.003 | - |
| slope | -29533 | 264.51 | 0.71 | 0.860 | 0.116 | - |
| road | -31537 | 2268.30 | 1.13 | 2.610 | -5.970 | - |
| path | -29435 | 166.79 | 0.71 | 1.508 | -1.921 | - |
| popn | -29487 | 218.88 | 0.73 | 1.348 | -0.003 | - |
| logpopn | -31528 | 2259.07 | 1.11 | 1.570 | -1.112 | - |
| elevSq | -31046 | 485.55 | 0.97 | -0.017 | 0.007 | 0.000 |
| slopeSq | -29603 | 69.46 | 0.73 | 0.630 | 0.231 | -0.009 |
| roadSq | -31620 | 83.19 | 1.13 | 2.839 | -8.657 | 5.105 |
| pathSq | -29442 | 6.35 | 0.71 | 1.558 | -2.811 | 1.688 |
| popnSq | -29665 | 177.83 | 0.76 | 1.413 | -0.006 | 0.000 |
| logpopnSq | -31729 | 200.89 | 1.17 | 1.777 | -1.073 | -0.281 |

1. Bryophyta

|  | Dev | DeltaDev | phi | Intercept | X | X^2^ |
| --- | --- | --- | --- | --- | --- | --- |
| Null | -15888 | 0.00 | 0.89 | 1.111 | - | - |
| elev | -16986 | 1098.13 | 1.08 | 0.467 | 0.002 | - |
| slope | -16538 | 649.93 | 1.00 | 0.531 | 0.171 | - |
| road | -17288 | 1400.44 | 1.19 | 2.033 | -4.264 | - |
| path | -15963 | 75.77 | 0.91 | 1.281 | -1.275 | - |
| popn | -15924 | 36.64 | 0.91 | 1.132 | -0.001 | - |
| logpopn | -16944 | 1056.68 | 1.08 | 1.235 | -0.633 | - |
| elevSq | -16987 | 1.08 | 1.08 | 0.494 | 0.002 | 0.000 |
| slopeSq | -16553 | 15.87 | 1.00 | 0.415 | 0.233 | -0.005 |
| roadSq | -17493 | 204.88 | 1.22 | 2.361 | -8.196 | 7.372 |
| pathSq | -15991 | 27.13 | 0.92 | 1.376 | -2.978 | 3.922 |
| popnSq | -15959 | 35.14 | 0.91 | 1.152 | -0.002 | 0.000 |
| logpopnSq | -16985 | 40.86 | 1.09 | 1.153 | -0.648 | 0.110 |

1. Fungi

|  | Dev | DeltaDev | phi | Intercept | X | X^2^ |
| --- | --- | --- | --- | --- | --- | --- |
| Null | -3953 | 0.00 | 1.32 | 0.719 | - | - |
| elev | -6331 | 2378.72 | 2.01 | -0.186 | 0.003 | - |
| slope | -5290 | 1337.15 | 1.66 | -0.055 | 0.219 | - |
| road | -5571 | 1618.36 | 1.82 | 1.591 | -4.320 | - |
| path | -4092 | 139.03 | 1.35 | 0.932 | -1.677 | - |
| popn | -3982 | 29.19 | 1.32 | 0.734 | -0.001 | - |
| logpopn | -5761 | 1807.91 | 1.85 | 0.833 | -0.769 | - |
| elevSq | -6795 | 463.13 | 2.21 | 0.294 | -0.001 | 0.000 |
| slopeSq | -5314 | 24.37 | 1.68 | 0.107 | 0.129 | 0.008 |
| roadSq | -6822 | 1251.33 | 2.27 | 2.339 | -14.078 | 18.702 |
| pathSq | -4190 | 98.20 | 1.38 | 1.102 | -4.690 | 7.232 |
| popnSq | -4015 | 33.37 | 1.33 | 0.750 | -0.002 | 0.000 |
| logpopnSq | -6223 | 461.84 | 2.00 | 0.583 | -0.817 | 0.345 |

1. Lichen

|  | Dev | DeltaDev | phi | Intercept | X | X^2^ |
| --- | --- | --- | --- | --- | --- | --- |
| Null | -8974 | 0.00 | 0.97 | 0.848 | - | - |
| elev | -10391 | 1417.34 | 1.23 | 0.133 | 0.002 | - |
| slope | -9720 | 745.85 | 1.09 | 0.262 | 0.167 | - |
| road | -10399 | 1424.75 | 1.29 | 1.738 | -4.248 | - |
| path | -9097 | 123.21 | 0.99 | 1.057 | -1.619 | - |
| popn | -8987 | 12.70 | 0.97 | 0.860 | 0.000 | - |
| logpopn | -10165 | 1191.12 | 1.20 | 0.951 | -0.648 | - |
| elevSq | -10653 | 262.01 | 1.29 | 0.526 | -0.001 | 0.000 |
| slopeSq | -9752 | 32.02 | 1.10 | 0.452 | 0.069 | 0.009 |
| roadSq | -11027 | 628.01 | 1.42 | 2.264 | -11.156 | 13.072 |
| pathSq | -9152 | 55.08 | 1.01 | 1.191 | -4.025 | 5.773 |
| popnSq | -8996 | 9.13 | 0.97 | 0.877 | -0.001 | 0.000 |
| logpopnSq | -10499 | 334.05 | 1.27 | 0.735 | -0.697 | 0.312 |

1. Amphibia

|  | Dev | DeltaDev | phi | Intercept | X | X^2^ |
| --- | --- | --- | --- | --- | --- | --- |
| Null | -70721 | 0.00 | 0.77 | 2.040 | - | - |
| elev | -71023 | 301.80 | 0.81 | 1.700 | 0.001 | - |
| slope | -70815 | 93.70 | 0.78 | 1.822 | 0.063 | - |
| road | -71237 | 515.78 | 0.85 | 2.585 | -2.442 | - |
| path | -70735 | 13.84 | 0.77 | 2.104 | -0.520 | - |
| popn | -70918 | 197.33 | 0.83 | 2.144 | -0.003 | - |
| logpopn | -71235 | 513.88 | 0.85 | 2.158 | -0.462 | - |
| elevSq | -71155 | 132.45 | 0.83 | 1.458 | 0.003 | 0.000 |
| slopeSq | -70847 | 32.16 | 0.78 | 1.663 | 0.137 | -0.006 |
| roadSq | -71248 | 11.55 | 0.85 | 2.507 | -1.419 | -2.089 |
| pathSq | -70737 | 2.09 | 0.77 | 2.131 | -1.018 | 0.974 |
| popnSq | -71032 | 113.65 | 0.85 | 2.170 | -0.005 | 0.000 |
| logpopnSq | -71398 | 163.49 | 0.89 | 2.342 | -0.448 | -0.211 |

1. Aves

|  | Dev | DeltaDev | phi | Intercept | X | X^2^ |
| --- | --- | --- | --- | --- | --- | --- |
| Null | -1595 | 0.00 | 1.22 | -0.280 | - | - |
| elev | -3087 | 1491.34 | 1.70 | -1.147 | 0.003 | - |
| slope | -2232 | 636.85 | 1.40 | -0.938 | 0.188 | - |
| road | -3361 | 1765.85 | 1.81 | 0.714 | -4.909 | - |
| path | -1752 | 157.15 | 1.25 | -0.042 | -1.850 | - |
| popn | -1807 | 212.01 | 1.28 | -0.214 | -0.003 | - |
| logpopn | -3719 | 2124.20 | 1.93 | -0.123 | -0.965 | - |
| elevSq | -3177 | 90.80 | 1.72 | -1.359 | 0.004 | 0.000 |
| slopeSq | -2258 | 26.17 | 1.40 | -1.080 | 0.268 | -0.007 |
| roadSq | -3747 | 385.60 | 1.96 | 1.134 | -10.180 | 9.408 |
| pathSq | -1786 | 33.91 | 1.27 | 0.077 | -3.767 | 4.263 |
| popnSq | -1942 | 134.95 | 1.31 | -0.182 | -0.005 | 0.000 |
| logpopnSq | -3722 | 2.98 | 1.93 | -0.100 | -0.964 | -0.035 |

1. Land mammals

|  | Dev | DeltaDev | phi | Intercept | X | X^2^ |
| --- | --- | --- | --- | --- | --- | --- |
| Null | -41181 | 0.00 | 0.80 | 1.666 | - | - |
| elev | -41497 | 316.64 | 0.86 | 1.319 | 0.001 | - |
| slope | -41314 | 132.88 | 0.83 | 1.405 | 0.075 | - |
| road | -41912 | 731.18 | 0.93 | 2.319 | -2.939 | - |
| path | -41258 | 76.80 | 0.82 | 1.829 | -1.218 | - |
| popn | -41333 | 152.44 | 0.85 | 1.738 | -0.002 | - |
| logpopn | -41785 | 604.63 | 0.90 | 1.774 | -0.485 | - |
| elevSq | -41535 | 37.97 | 0.87 | 1.200 | 0.002 | 0.000 |
| slopeSq | -41326 | 12.03 | 0.83 | 1.305 | 0.126 | -0.004 |
| roadSq | -41914 | 2.04 | 0.93 | 2.350 | -3.401 | 0.888 |
| pathSq | -41259 | 1.90 | 0.82 | 1.864 | -1.802 | 1.019 |
| popnSq | -41436 | 102.70 | 0.87 | 1.779 | -0.004 | 0.000 |
| logpopnSq | -41816 | 30.16 | 0.92 | 1.856 | -0.476 | -0.091 |

1. Vascular plants

|  | Dev | DeltaDev | phi | Intercept | X | X^2^ |
| --- | --- | --- | --- | --- | --- | --- |
| Null | -2500 | 0.00 | 1.28 | 0.518 | - | - |
| elev | -3970 | 1470.32 | 1.72 | -0.251 | 0.002 | - |
| slope | -3212 | 712.28 | 1.48 | -0.102 | 0.178 | - |
| road | -3975 | 1475.65 | 1.77 | 1.412 | -4.276 | - |
| path | -2594 | 94.75 | 1.31 | 0.699 | -1.413 | - |
| popn | -2552 | 52.92 | 1.31 | 0.547 | -0.001 | - |
| logpopn | -3951 | 1451.52 | 1.73 | 0.652 | -0.747 | - |
| elevSq | -3990 | 20.28 | 1.72 | -0.151 | 0.002 | 0.000 |
| slopeSq | -3216 | 4.56 | 1.48 | -0.163 | 0.209 | -0.003 |
| roadSq | -4501 | 525.82 | 1.94 | 1.919 | -10.560 | 11.660 |
| pathSq | -2629 | 34.77 | 1.32 | 0.812 | -3.336 | 4.599 |
| popnSq | -2606 | 53.51 | 1.32 | 0.571 | -0.003 | 0.000 |
| logpopnSq | -4008 | 56.48 | 1.74 | 0.563 | -0.767 | 0.123 |

1. Poacea

|  | Dev | DeltaDev | phi | Intercept | X | X^2^ |
| --- | --- | --- | --- | --- | --- | --- |
| Null | -16767 | 0 | 0.8 | 1.035 | - | - |
| elev | -17448 | 681.06 | 0.91 | 0.509 | 0.002 | - |
| slope | -17256 | 488.94 | 0.87 | 0.500 | 0.161 | - |
| road | -17749 | 982.02 | 0.99 | 1.836 | -3.721 | - |
| path | -16813 | 45.74 | 0.81 | 1.169 | -1.014 | - |
| popn | -16857 | 89.44 | 0.82 | 1.079 | -0.002 | - |
| logpopn | -17513 | 745.92 | 0.93 | 1.164 | -0.575 | - |
| elevSq | -17480 | 32.25 | 0.92 | 0.374 | 0.003 | 0.000 |
| slopeSq | -17325 | 69.3 | 0.88 | 0.263 | 0.275 | -0.010 |
| roadSq | -17788 | 39.22 | 0.99 | 1.977 | -5.450 | 3.359 |
| pathSq | -16824 | 10.77 | 0.81 | 1.225 | -2.206 | 2.664 |
| popnSq | -16939 | 82.56 | 0.84 | 1.115 | -0.004 | 0.000 |
| logpopnSq | -17548 | 34.9 | 0.94 | 1.238 | -0.571 | -0.104 |

**O_0.5_ = 5**

1. Araneae

|  | Dev | DeltaDev | phi | Intercept | X | X^2^ |
| --- | --- | --- | --- | --- | --- | --- |
| Null | -80679 | 0 | 1.95 | 3.016 | - | - |
| elev | -80907 | 227.83 | 2.01 | 2.740 | 0.001 | - |
| slope | -80751 | 72.14 | 1.98 | 2.833 | 0.055 | - |
| road | -81109 | 429.42 | 2.13 | 3.487 | -2.119 | - |
| path | -80719 | 39.97 | 1.96 | 3.113 | -0.779 | - |
| popn | -80869 | 189.47 | 2.05 | 3.099 | -0.003 | - |
| logpopn | -81088 | 408.97 | 2.1 | 3.114 | -0.389 | - |
| elevSq | -81006 | 99.3 | 2.07 | 2.557 | 0.003 | 0.000 |
| slopeSq | -80772 | 20.43 | 1.97 | 2.709 | 0.111 | -0.004 |
| roadSq | -81126 | 17.78 | 2.16 | 3.399 | -1.076 | -1.940 |
| pathSq | -80720 | 1.19 | 1.96 | 3.135 | -1.152 | 0.953 |
| popnSq | -80993 | 124.23 | 2.1 | 3.141 | -0.004 | 0.000 |
| logpopnSq | -81240 | 151.76 | 2.22 | 3.297 | -0.366 | -0.189 |

1. Coleaoptera

|  | Dev | DeltaDev | phi | Intercept | X | X^2^ |
| --- | --- | --- | --- | --- | --- | --- |
| Null | -14093 | 0.00 | 1.98 | 1.670 | - | - |
| elev | -15716 | 1622.89 | 2.72 | 0.999 | 0.002 | - |
| slope | -14527 | 433.82 | 2.14 | 1.279 | 0.118 | - |
| road | -16146 | 2053.45 | 3.03 | 2.663 | -4.364 | - |
| path | -14284 | 190.81 | 2.06 | 1.901 | -1.755 | - |
| popn | -14219 | 126.34 | 2.06 | 1.723 | -0.002 | - |
| logpopn | -16351 | 2258.51 | 3.13 | 1.927 | -0.855 | - |
| elevSq | -15726 | 10.47 | 2.72 | 0.938 | 0.003 | 0.000 |
| slopeSq | -14530 | 2.97 | 2.15 | 1.327 | 0.093 | 0.002 |
| roadSq | -16639 | 493.23 | 3.34 | 3.174 | -10.010 | 9.912 |
| pathSq | -14338 | 54.44 | 2.08 | 2.024 | -3.861 | 5.016 |
| popnSq | -14326 | 107.21 | 2.12 | 1.754 | -0.004 | 0.000 |
| logpopnSq | -16377 | 25.37 | 3.16 | 1.875 | -0.885 | 0.079 |

1. Opilionidae

|  | Dev | DeltaDev | phi | Intercept | X | X^2^ |
| --- | --- | --- | --- | --- | --- | --- |
| Null | -107785 | 0.00 | 7.54 | 4.529 | - | - |
| elev | -107796 | 11.11 | 7.60 | 4.469 | 0.000 | - |
| slope | -107789 | 4.02 | 7.56 | 4.490 | 0.014 | - |
| road | -107807 | 22.41 | 7.57 | 4.614 | -0.457 | - |
| path | -107785 | 0.33 | 7.61 | 4.553 | -0.128 | - |
| popn | -107858 | 73.46 | 7.87 | 4.583 | -0.001 | - |
| logpopn | -107809 | 24.01 | 7.66 | 4.550 | -0.085 | - |
| elevSq | -107802 | 6.44 | 7.53 | 4.422 | 0.001 | 0.000 |
| slopeSq | -107790 | 1.28 | 7.52 | 4.432 | 0.030 | -0.001 |
| roadSq | -107809 | 1.82 | 7.68 | 4.600 | -0.109 | -0.621 |
| pathSq | -107784 | -1.24 | 7.51 | 4.547 | -0.174 | 0.128 |
| popnSq | -107866 | 7.77 | 7.90 | 4.595 | -0.001 | 0.000 |
| logpopnSq | -107827 | 18.65 | 7.66 | 4.603 | -0.079 | -0.065 |

1. Odonata

|  | Dev | DeltaDev | phi | Intercept | X | X^2^ |
| --- | --- | --- | --- | --- | --- | --- |
| Null | -51916 | 0.00 | 1.16 | 2.175 | - | - |
| elev | -52764 | 848.54 | 1.35 | 1.636 | 0.002 | - |
| slope | -52157 | 241.15 | 1.20 | 1.837 | 0.101 | - |
| road | -53322 | 1406.64 | 1.58 | 3.136 | -4.075 | - |
| path | -51991 | 75.50 | 1.16 | 2.325 | -1.154 | - |
| popn | -52170 | 254.63 | 1.26 | 2.290 | -0.004 | - |
| logpopn | -53156 | 1240.73 | 1.49 | 2.394 | -0.716 | - |
| elevSq | -52964 | 199.99 | 1.40 | 1.365 | 0.004 | 0.000 |
| slopeSq | -52194 | 37.44 | 1.21 | 1.691 | 0.183 | -0.007 |
| roadSq | -53322 | 0.07 | 1.58 | 3.126 | -3.808 | -0.537 |
| pathSq | -51994 | 3.28 | 1.18 | 2.371 | -1.665 | 1.256 |
| popnSq | -52342 | 171.73 | 1.32 | 2.344 | -0.006 | 0.000 |
| logpopnSq | -53323 | 166.47 | 1.57 | 2.576 | -0.681 | -0.215 |

1. Papilionoidea

|  | Dev | DeltaDev | phi | Intercept | X | X^2^ |
| --- | --- | --- | --- | --- | --- | --- |
| Null | -33903 | 0.00 | 0.96 | 1.673 | - | - |
| elev | -35260 | 1356.77 | 1.25 | 0.940 | 0.003 | - |
| slope | -34177 | 274.48 | 1.00 | 1.301 | 0.115 | - |
| road | -36361 | 2458.32 | 1.65 | 3.051 | -5.827 | - |
| path | -34077 | 174.46 | 0.99 | 1.927 | -1.892 | - |
| popn | -34183 | 280.03 | 1.06 | 1.803 | -0.004 | - |
| logpopn | -36331 | 2427.84 | 1.62 | 2.042 | -1.102 | - |
| elevSq | -35766 | 505.89 | 1.39 | 0.484 | 0.007 | 0.000 |
| slopeSq | -34249 | 71.18 | 1.02 | 1.089 | 0.228 | -0.009 |
| roadSq | -36431 | 69.52 | 1.66 | 3.276 | -8.155 | 4.356 |
| pathSq | -34083 | 5.33 | 0.99 | 1.963 | -2.584 | 1.656 |
| popnSq | -34393 | 210.01 | 1.11 | 1.860 | -0.007 | 0.000 |
| logpopnSq | -36566 | 235.13 | 1.71 | 2.249 | -1.042 | -0.287 |

1. Bryophyta

|  | Dev | DeltaDev | phi | Intercept | X | X^2^ |
| --- | --- | --- | --- | --- | --- | --- |
| Null | -20385 | 0.00 | 1.37 | 1.634 | - | - |
| elev | -21535 | 1150.57 | 1.66 | 1.042 | 0.002 | - |
| slope | -21065 | 679.96 | 1.53 | 1.097 | 0.161 | - |
| road | -21919 | 1533.96 | 1.86 | 2.546 | -4.065 | - |
| path | -20466 | 81.60 | 1.39 | 1.796 | -1.224 | - |
| popn | -20426 | 41.33 | 1.38 | 1.654 | -0.001 | - |
| logpopn | -21500 | 1115.03 | 1.66 | 1.772 | -0.599 | - |
| elevSq | -21537 | 1.91 | 1.66 | 1.077 | 0.002 | 0.000 |
| slopeSq | -21076 | 11.18 | 1.53 | 1.020 | 0.207 | -0.004 |
| roadSq | -22136 | 217.01 | 1.93 | 2.875 | -7.784 | 6.876 |
| pathSq | -20495 | 28.31 | 1.39 | 1.876 | -2.841 | 3.717 |
| popnSq | -20463 | 37.02 | 1.39 | 1.674 | -0.002 | 0.000 |
| logpopnSq | -21552 | 51.99 | 1.68 | 1.693 | -0.626 | 0.108 |

1. Fungi

|  | Dev | DeltaDev | phi | Intercept | X | X^2^ |
| --- | --- | --- | --- | --- | --- | --- |
| Null | -7737 | 0.00 | 2.02 | 1.312 | - | - |
| elev | -10200 | 2463.44 | 3.14 | 0.552 | 0.003 | - |
| slope | -9118 | 1381.42 | 2.58 | 0.640 | 0.199 | - |
| road | -9487 | 1750.04 | 2.84 | 2.134 | -3.807 | - |
| path | -7894 | 157.17 | 2.10 | 1.518 | -1.576 | - |
| popn | -7771 | 33.96 | 2.05 | 1.331 | -0.001 | - |
| logpopn | -9647 | 1910.03 | 2.88 | 1.461 | -0.694 | - |
| elevSq | -10728 | 527.56 | 3.54 | 1.010 | -0.001 | 0.000 |
| slopeSq | -9165 | 46.92 | 2.63 | 0.830 | 0.092 | 0.011 |
| roadSq | -10822 | 1335.81 | 3.64 | 2.880 | -12.949 | 17.113 |
| pathSq | -7998 | 104.57 | 2.14 | 1.687 | -4.509 | 6.820 |
| popnSq | -7812 | 41.61 | 2.08 | 1.347 | -0.002 | 0.000 |
| logpopnSq | -10174 | 527.43 | 3.21 | 1.233 | -0.784 | 0.342 |

1. Lichen

|  | Dev | DeltaDev | phi | Intercept | X | X^2^ |
| --- | --- | --- | --- | --- | --- | --- |
| Null | -12669 | 0.00 | 1.44 | 1.371 | - | - |
| elev | -14129 | 1460.42 | 1.83 | 0.738 | 0.002 | - |
| slope | -13433 | 764.67 | 1.62 | 0.848 | 0.153 | - |
| road | -14222 | 1552.91 | 1.94 | 2.222 | -3.937 | - |
| path | -12806 | 136.94 | 1.48 | 1.578 | -1.581 | - |
| popn | -12684 | 14.99 | 1.44 | 1.385 | 0.000 | - |
| logpopn | -13905 | 1236.57 | 1.78 | 1.486 | -0.590 | - |
| elevSq | -14429 | 299.72 | 1.95 | 1.119 | -0.001 | 0.000 |
| slopeSq | -13483 | 49.85 | 1.65 | 1.046 | 0.041 | 0.010 |
| roadSq | -14877 | 654.93 | 2.17 | 2.765 | -10.387 | 11.988 |
| pathSq | -12862 | 56.54 | 1.49 | 1.701 | -3.801 | 5.333 |
| popnSq | -12694 | 9.88 | 1.45 | 1.392 | -0.001 | 0.000 |
| logpopnSq | -14291 | 386.12 | 1.92 | 1.286 | -0.671 | 0.303 |

1. Amphibia

|  | Dev | DeltaDev | phi | Intercept | X | X^2^ |
| --- | --- | --- | --- | --- | --- | --- |
| Null | -77669 | 0.00 | 1.11 | 2.464 | - | - |
| elev | -77966 | 297.06 | 1.17 | 2.140 | 0.001 | - |
| slope | -77761 | 92.06 | 1.13 | 2.252 | 0.063 | - |
| road | -78177 | 508.24 | 1.22 | 2.983 | -2.361 | - |
| path | -77682 | 13.48 | 1.11 | 2.520 | -0.474 | - |
| popn | -77883 | 214.91 | 1.20 | 2.573 | -0.003 | - |
| logpopn | -78176 | 507.71 | 1.23 | 2.583 | -0.447 | - |
| elevSq | -78094 | 128.67 | 1.20 | 1.912 | 0.003 | 0.000 |
| slopeSq | -77792 | 31.72 | 1.13 | 2.110 | 0.134 | -0.006 |
| roadSq | -78191 | 14.24 | 1.25 | 2.909 | -1.300 | -1.950 |
| pathSq | -77684 | 2.15 | 1.11 | 2.558 | -0.894 | 1.048 |
| popnSq | -77989 | 105.93 | 1.23 | 2.602 | -0.005 | 0.000 |
| logpopnSq | -78339 | 162.47 | 1.28 | 2.756 | -0.421 | -0.198 |

1. Aves

|  | Dev | DeltaDev | phi | Intercept | X | X^2^ |
| --- | --- | --- | --- | --- | --- | --- |
| Null | -782 | 0.00 | 1.35 | 0.148 | - | - |
| elev | -2559 | 1776.73 | 1.99 | -0.746 | 0.003 | - |
| slope | -1490 | 707.82 | 1.57 | -0.502 | 0.189 | - |
| road | -3121 | 2338.21 | 2.27 | 1.295 | -5.528 | - |
| path | -979 | 196.70 | 1.41 | 0.414 | -2.025 | - |
| popn | -1098 | 315.88 | 1.46 | 0.239 | -0.004 | - |
| logpopn | -3531 | 2748.98 | 2.47 | 0.386 | -1.094 | - |
| elevSq | -2743 | 183.55 | 2.06 | -1.028 | 0.005 | 0.000 |
| slopeSq | -1534 | 44.34 | 1.58 | -0.697 | 0.294 | -0.009 |
| roadSq | -3483 | 362.19 | 2.43 | 1.697 | -10.376 | 8.904 |
| pathSq | -1014 | 35.12 | 1.42 | 0.521 | -3.964 | 4.206 |
| popnSq | -1284 | 185.89 | 1.51 | 0.286 | -0.006 | 0.000 |
| logpopnSq | -3581 | 49.33 | 2.51 | 0.470 | -1.080 | -0.121 |

1. Land mammals

|  | Dev | DeltaDev | phi | Intercept | X | X^2^ |
| --- | --- | --- | --- | --- | --- | --- |
| Null | -47245 | 0.00 | 1.26 | 2.167 | - | - |
| elev | -47569 | 323.50 | 1.33 | 1.858 | 0.001 | - |
| slope | -47381 | 135.42 | 1.28 | 1.927 | 0.073 | - |
| road | -48004 | 758.21 | 1.47 | 2.827 | -2.864 | - |
| path | -47324 | 78.58 | 1.27 | 2.330 | -1.193 | - |
| popn | -47426 | 180.86 | 1.34 | 2.261 | -0.003 | - |
| logpopn | -47868 | 622.95 | 1.41 | 2.295 | -0.469 | - |
| elevSq | -47607 | 38.32 | 1.35 | 1.729 | 0.002 | 0.000 |
| slopeSq | -47392 | 11.45 | 1.30 | 1.848 | 0.116 | -0.004 |
| roadSq | -48004 | 0.27 | 1.46 | 2.838 | -3.253 | 0.497 |
| pathSq | -47326 | 1.64 | 1.27 | 2.360 | -1.699 | 1.058 |
| popnSq | -47532 | 106.17 | 1.35 | 2.308 | -0.004 | 0.000 |
| logpopnSq | -47902 | 33.87 | 1.43 | 2.374 | -0.460 | -0.097 |

1. Vascular plants

|  | Dev | DeltaDev | phi | Intercept | X | X^2^ |
| --- | --- | --- | --- | --- | --- | --- |
| Null | -5410 | 0.00 | 1.87 | 1.074 | - | - |
| elev | -7020 | 1609.96 | 2.54 | 0.380 | 0.002 | - |
| slope | -6182 | 771.95 | 2.15 | 0.500 | 0.170 | - |
| road | -7153 | 1742.81 | 2.67 | 1.976 | -4.161 | - |
| path | -5522 | 111.54 | 1.90 | 1.255 | -1.403 | - |
| popn | -5480 | 70.28 | 1.89 | 1.106 | -0.001 | - |
| logpopn | -7070 | 1659.44 | 2.60 | 1.247 | -0.725 | - |
| elevSq | -7038 | 18.20 | 2.56 | 0.476 | 0.002 | 0.000 |
| slopeSq | -6185 | 2.96 | 2.16 | 0.450 | 0.198 | -0.002 |
| roadSq | -7710 | 556.60 | 2.98 | 2.479 | -10.003 | 10.679 |
| pathSq | -5559 | 37.20 | 1.91 | 1.374 | -3.212 | 4.254 |
| popnSq | -5548 | 67.40 | 1.92 | 1.130 | -0.003 | 0.000 |
| logpopnSq | -7121 | 51.60 | 2.63 | 1.170 | -0.755 | 0.110 |

1. Poacea

|  | Dev | DeltaDev | phi | Intercept | X | X^2^ |
| --- | --- | --- | --- | --- | --- | --- |
| Null | -20824 | 0.00 | 1.15 | 1.505 | - | - |
| elev | -21545 | 721.35 | 1.32 | 1.008 | 0.002 | - |
| slope | -21342 | 518.74 | 1.27 | 0.996 | 0.156 | - |
| road | -21912 | 1088.74 | 1.45 | 2.315 | -3.650 | - |
| path | -20873 | 49.17 | 1.17 | 1.630 | -0.957 | - |
| popn | -20936 | 111.99 | 1.19 | 1.559 | -0.002 | - |
| logpopn | -21640 | 816.44 | 1.36 | 1.649 | -0.570 | - |
| elevSq | -21584 | 39.01 | 1.33 | 0.876 | 0.003 | 0.000 |
| slopeSq | -21412 | 69.26 | 1.28 | 0.779 | 0.262 | -0.009 |
| roadSq | -21950 | 37.78 | 1.46 | 2.464 | -5.305 | 3.154 |
| pathSq | -20884 | 10.98 | 1.16 | 1.691 | -2.096 | 2.578 |
| popnSq | -21031 | 95.33 | 1.21 | 1.594 | -0.004 | 0.000 |
| logpopnSq | -21686 | 46.23 | 1.37 | 1.740 | -0.562 | -0.109 |

**O_0.5_ = 10**

1. Araneae

|  | Dev | DeltaDev | phi | Intercept | X | X^2^ |
| --- | --- | --- | --- | --- | --- | --- |
| Null | -86890 | 0.00 | 3.19 | 3.541 | - | - |
| elev | -87117 | 227.16 | 3.38 | 3.266 | 0.001 | - |
| slope | -86962 | 72.13 | 3.25 | 3.356 | 0.052 | - |
| road | -87319 | 428.24 | 3.51 | 4.010 | -2.062 | - |
| path | -86930 | 39.54 | 3.22 | 3.640 | -0.790 | - |
| popn | -87050 | 159.31 | 3.26 | 3.577 | -0.002 | - |
| logpopn | -87299 | 408.80 | 3.50 | 3.652 | -0.386 | - |
| elevSq | -87215 | 97.91 | 3.41 | 3.097 | 0.002 | 0.000 |
| slopeSq | -86983 | 20.19 | 3.27 | 3.254 | 0.107 | -0.004 |
| roadSq | -87335 | 16.50 | 3.52 | 3.908 | -1.007 | -1.842 |
| pathSq | -86931 | 1.23 | 3.22 | 3.660 | -1.141 | 0.952 |
| popnSq | -87196 | 146.75 | 3.48 | 3.664 | -0.004 | 0.000 |
| logpopnSq | -87449 | 150.18 | 3.69 | 3.811 | -0.356 | -0.177 |

1. Coleaoptera

|  | Dev | DeltaDev | phi | Intercept | X | X^2^ |
| --- | --- | --- | --- | --- | --- | --- |
| Null | -19154 | 0.00 | 3.18 | 2.212 | - | - |
| elev | -20808 | 1654.24 | 4.45 | 1.593 | 0.002 | - |
| slope | -19592 | 437.57 | 3.46 | 1.845 | 0.113 | - |
| road | -21240 | 2085.98 | 4.92 | 3.175 | -4.038 | - |
| path | -19349 | 195.26 | 3.31 | 2.446 | -1.655 | - |
| popn | -19271 | 116.95 | 3.26 | 2.251 | -0.002 | - |
| logpopn | -21464 | 2309.97 | 5.13 | 2.494 | -0.807 | - |
| elevSq | -20817 | 8.43 | 4.45 | 1.541 | 0.003 | 0.000 |
| slopeSq | -19595 | 3.61 | 3.44 | 1.902 | 0.085 | 0.003 |
| roadSq | -21768 | 527.63 | 5.57 | 3.700 | -9.598 | 9.570 |
| pathSq | -19403 | 54.03 | 3.35 | 2.552 | -3.713 | 4.769 |
| popnSq | -19392 | 121.28 | 3.35 | 2.286 | -0.003 | 0.000 |
| logpopnSq | -21502 | 38.38 | 5.23 | 2.444 | -0.858 | 0.083 |

1. Opilionidae

|  | Dev | DeltaDev | phi | Intercept | X | X^2^ |
| --- | --- | --- | --- | --- | --- | --- |
| Null | -114351 | 0.00 | 13.59 | 5.127 | - | - |
| elev | -114362 | 10.95 | 13.88 | 5.081 | 0.000 | - |
| slope | -114355 | 4.01 | 13.57 | 5.082 | 0.013 | - |
| road | -114373 | 22.13 | 13.85 | 5.206 | -0.438 | - |
| path | -114352 | 0.22 | 13.62 | 5.142 | -0.128 | - |
| popn | -114423 | 71.97 | 14.05 | 5.181 | -0.001 | - |
| logpopn | -114375 | 23.76 | 13.75 | 5.136 | -0.082 | - |
| elevSq | -114369 | 6.25 | 13.80 | 5.008 | 0.001 | 0.000 |
| slopeSq | -114356 | 0.97 | 13.67 | 5.065 | 0.026 | -0.001 |
| roadSq | -114375 | 1.50 | 13.89 | 5.195 | -0.079 | -0.637 |
| pathSq | -114351 | -0.88 | 13.76 | 5.146 | -0.269 | 0.167 |
| popnSq | -114432 | 8.64 | 14.06 | 5.189 | -0.001 | 0.000 |
| logpopnSq | -114394 | 18.67 | 13.92 | 5.198 | -0.084 | -0.066 |

1. Odonata

|  | Dev | DeltaDev | phi | Intercept | X | X^2^ |
| --- | --- | --- | --- | --- | --- | --- |
| Null | -57382 | 0.00 | 1.73 | 2.620 | - | - |
| elev | -58233 | 851.24 | 2.01 | 2.102 | 0.002 | - |
| slope | -57624 | 241.56 | 1.78 | 2.288 | 0.098 | - |
| road | -58809 | 1426.52 | 2.41 | 3.583 | -3.992 | - |
| path | -57458 | 75.35 | 1.74 | 2.755 | -1.101 | - |
| popn | -57648 | 266.20 | 1.88 | 2.726 | -0.003 | - |
| logpopn | -58635 | 1252.98 | 2.25 | 2.846 | -0.701 | - |
| elevSq | -58430 | 196.42 | 2.12 | 1.839 | 0.004 | 0.000 |
| slopeSq | -57660 | 36.08 | 1.81 | 2.146 | 0.173 | -0.006 |
| roadSq | -58811 | 2.62 | 2.42 | 3.544 | -3.440 | -0.902 |
| pathSq | -57461 | 3.00 | 1.73 | 2.792 | -1.616 | 1.500 |
| popnSq | -57816 | 168.08 | 1.94 | 2.775 | -0.006 | 0.000 |
| logpopnSq | -58798 | 163.28 | 2.36 | 3.013 | -0.653 | -0.199 |

1. Papilionoidea

|  | Dev | DeltaDev | phi | Intercept | X | X^2^ |
| --- | --- | --- | --- | --- | --- | --- |
| Null | -38556 | 0.00 | 1.32 | 2.062 | - | - |
| elev | -39929 | 1373.39 | 1.74 | 1.356 | 0.003 | - |
| slope | -38832 | 276.54 | 1.38 | 1.695 | 0.111 | - |
| road | -41055 | 2499.23 | 2.31 | 3.413 | -5.542 | - |
| path | -38731 | 175.29 | 1.36 | 2.295 | -1.806 | - |
| popn | -38874 | 317.84 | 1.48 | 2.198 | -0.004 | - |
| logpopn | -41032 | 2476.59 | 2.30 | 2.456 | -1.078 | - |
| elevSq | -40428 | 498.80 | 1.93 | 0.937 | 0.006 | 0.000 |
| slopeSq | -38903 | 71.11 | 1.41 | 1.491 | 0.217 | -0.009 |
| roadSq | -41124 | 68.48 | 2.34 | 3.607 | -7.809 | 3.933 |
| pathSq | -38737 | 5.45 | 1.36 | 2.341 | -2.498 | 1.620 |
| popnSq | -39097 | 223.12 | 1.55 | 2.266 | -0.007 | 0.000 |
| logpopnSq | -41254 | 221.75 | 2.41 | 2.647 | -1.002 | -0.263 |

1. Bryophyta

|  | Dev | DeltaDev | phi | Intercept | X | X^2^ |
| --- | --- | --- | --- | --- | --- | --- |
| Null | -25183 | 0.00 | 2.07 | 2.116 | - | - |
| elev | -26346 | 1162.47 | 2.55 | 1.577 | 0.002 | - |
| slope | -25871 | 687.51 | 2.33 | 1.610 | 0.154 | - |
| road | -26744 | 1560.16 | 2.84 | 2.996 | -3.806 | - |
| path | -25267 | 83.33 | 2.09 | 2.261 | -1.148 | - |
| popn | -25224 | 40.51 | 2.08 | 2.139 | -0.001 | - |
| logpopn | -26308 | 1124.78 | 2.54 | 2.265 | -0.565 | - |
| elevSq | -26349 | 3.32 | 2.55 | 1.614 | 0.002 | 0.000 |
| slopeSq | -25878 | 7.35 | 2.32 | 1.546 | 0.193 | -0.003 |
| roadSq | -26986 | 242.16 | 2.98 | 3.341 | -7.569 | 6.454 |
| pathSq | -25295 | 28.21 | 2.11 | 2.363 | -2.678 | 3.780 |
| popnSq | -25263 | 39.23 | 2.10 | 2.155 | -0.002 | 0.000 |
| logpopnSq | -26372 | 63.35 | 2.59 | 2.188 | -0.609 | 0.111 |

1. Fungi

|  | Dev | DeltaDev | phi | Intercept | X | X^2^ |
| --- | --- | --- | --- | --- | --- | --- |
| Null | -12204 | 0.00 | 3.13 | 1.845 | - | - |
| elev | -14658 | 2454.11 | 4.89 | 1.179 | 0.002 | - |
| slope | -13581 | 1377.06 | 3.99 | 1.232 | 0.185 | - |
| road | -13944 | 1740.21 | 4.36 | 2.606 | -3.381 | - |
| path | -12365 | 161.86 | 3.23 | 2.042 | -1.469 | - |
| popn | -12236 | 32.80 | 3.15 | 1.858 | -0.001 | - |
| logpopn | -14109 | 1905.40 | 4.47 | 2.012 | -0.632 | - |
| elevSq | -15225 | 567.76 | 5.55 | 1.609 | -0.001 | 0.000 |
| slopeSq | -13646 | 64.88 | 4.09 | 1.423 | 0.071 | 0.012 |
| roadSq | -15330 | 1386.48 | 5.77 | 3.376 | -12.088 | 15.975 |
| pathSq | -12472 | 106.84 | 3.28 | 2.209 | -4.269 | 6.430 |
| popnSq | -12282 | 46.08 | 3.19 | 1.879 | -0.002 | 0.000 |
| logpopnSq | -14685 | 576.42 | 5.05 | 1.804 | -0.755 | 0.331 |

1. Lichen

|  | Dev | DeltaDev | phi | Intercept | X | X^2^ |
| --- | --- | --- | --- | --- | --- | --- |
| Null | -17026 | 0.00 | 2.13 | 1.847 | - | - |
| elev | -18480 | 1453.57 | 2.72 | 1.287 | 0.002 | - |
| slope | -17787 | 760.74 | 2.41 | 1.372 | 0.143 | - |
| road | -18583 | 1556.48 | 2.88 | 2.652 | -3.574 | - |
| path | -17167 | 140.47 | 2.19 | 2.049 | -1.504 | - |
| popn | -17042 | 15.51 | 2.14 | 1.859 | 0.000 | - |
| logpopn | -18252 | 1225.73 | 2.64 | 1.973 | -0.547 | - |
| elevSq | -18804 | 324.54 | 2.92 | 1.633 | -0.001 | 0.000 |
| slopeSq | -17850 | 62.69 | 2.47 | 1.573 | 0.024 | 0.011 |
| roadSq | -19268 | 685.50 | 3.26 | 3.207 | -9.844 | 11.308 |
| pathSq | -17224 | 57.35 | 2.22 | 2.172 | -3.701 | 4.844 |
| popnSq | -17052 | 10.27 | 2.15 | 1.872 | -0.001 | 0.000 |
| logpopnSq | -18675 | 423.22 | 2.87 | 1.784 | -0.649 | 0.297 |

1. Amphibia

|  | Dev | DeltaDev | phi | Intercept | X | X^2^ |
| --- | --- | --- | --- | --- | --- | --- |
| Null | -83432 | 0.00 | 1.55 | 2.834 | - | - |
| elev | -83725 | 292.77 | 1.66 | 2.545 | 0.001 | - |
| slope | -83523 | 90.69 | 1.61 | 2.643 | 0.061 | - |
| road | -83933 | 500.22 | 1.74 | 3.372 | -2.296 | - |
| path | -83446 | 13.34 | 1.56 | 2.904 | -0.469 | - |
| popn | -83654 | 222.00 | 1.71 | 2.957 | -0.003 | - |
| logpopn | -83933 | 500.96 | 1.75 | 2.963 | -0.432 | - |
| elevSq | -83851 | 125.67 | 1.69 | 2.319 | 0.003 | 0.000 |
| slopeSq | -83554 | 30.92 | 1.60 | 2.525 | 0.131 | -0.005 |
| roadSq | -83948 | 14.83 | 1.75 | 3.264 | -1.242 | -1.983 |
| pathSq | -83447 | 1.45 | 1.58 | 2.932 | -0.895 | 0.923 |
| popnSq | -83753 | 98.85 | 1.73 | 2.977 | -0.004 | 0.000 |
| logpopnSq | -84092 | 158.33 | 1.82 | 3.122 | -0.403 | -0.192 |

1. Aves

|  | Dev | DeltaDev | phi | Intercept | X | X^2^ |
| --- | --- | --- | --- | --- | --- | --- |
| Null | -1717 | 0.00 | 1.51 | 0.516 | - | - |
| elev | -3547 | 1829.36 | 2.25 | -0.346 | 0.003 | - |
| slope | -2421 | 703.11 | 1.75 | -0.104 | 0.183 | - |
| road | -4309 | 2591.65 | 2.68 | 1.722 | -5.575 | - |
| path | -1929 | 211.38 | 1.58 | 0.783 | -2.050 | - |
| popn | -2127 | 409.55 | 1.70 | 0.626 | -0.004 | - |
| logpopn | -4754 | 3036.33 | 2.99 | 0.821 | -1.141 | - |
| elevSq | -3796 | 248.71 | 2.39 | -0.657 | 0.006 | 0.000 |
| slopeSq | -2474 | 53.32 | 1.77 | -0.304 | 0.290 | -0.009 |
| roadSq | -4596 | 286.55 | 2.83 | 2.074 | -9.751 | 7.674 |
| pathSq | -1960 | 31.33 | 1.59 | 0.890 | -3.854 | 4.006 |
| popnSq | -2356 | 228.53 | 1.78 | 0.680 | -0.007 | 0.000 |
| logpopnSq | -4877 | 123.39 | 3.09 | 0.950 | -1.102 | -0.186 |

1. Land mammals

|  | Dev | DeltaDev | phi | Intercept | X | X^2^ |
| --- | --- | --- | --- | --- | --- | --- |
| Null | -52748 | 0.00 | 1.91 | 2.644 | - | - |
| elev | -53073 | 325.21 | 2.03 | 2.322 | 0.001 | - |
| slope | -52884 | 135.83 | 1.96 | 2.412 | 0.072 | - |
| road | -53513 | 764.93 | 2.28 | 3.281 | -2.750 | - |
| path | -52827 | 78.83 | 1.94 | 2.799 | -1.139 | - |
| popn | -52938 | 190.07 | 2.05 | 2.719 | -0.002 | - |
| logpopn | -53376 | 628.08 | 2.18 | 2.773 | -0.459 | - |
| elevSq | -53112 | 38.52 | 2.05 | 2.214 | 0.002 | 0.000 |
| slopeSq | -52894 | 10.43 | 1.96 | 2.325 | 0.112 | -0.004 |
| roadSq | -53513 | 0.42 | 2.26 | 3.306 | -3.062 | 0.634 |
| pathSq | -52828 | 1.57 | 1.94 | 2.835 | -1.630 | 1.060 |
| popnSq | -53042 | 104.35 | 2.08 | 2.760 | -0.004 | 0.000 |
| logpopnSq | -53411 | 34.56 | 2.20 | 2.846 | -0.444 | -0.083 |

1. Vascular plants

|  | Dev | DeltaDev | phi | Intercept | X | X^2^ |
| --- | --- | --- | --- | --- | --- | --- |
| Null | -9311 | 0.00 | 2.72 | 1.573 | - | - |
| elev | -10945 | 1633.87 | 3.75 | 0.947 | 0.002 | - |
| slope | -10093 | 781.77 | 3.15 | 1.045 | 0.162 | - |
| road | -11109 | 1797.21 | 3.94 | 2.439 | -3.788 | - |
| path | -9428 | 116.88 | 2.77 | 1.745 | -1.335 | - |
| popn | -9383 | 72.14 | 2.76 | 1.604 | -0.001 | - |
| logpopn | -11025 | 1713.23 | 3.85 | 1.772 | -0.686 | - |
| elevSq | -10965 | 20.14 | 3.75 | 1.032 | 0.001 | 0.000 |
| slopeSq | -10094 | 0.81 | 3.16 | 1.027 | 0.177 | -0.001 |
| roadSq | -11699 | 590.47 | 4.46 | 2.953 | -9.327 | 9.872 |
| pathSq | -9465 | 37.23 | 2.80 | 1.849 | -3.071 | 4.039 |
| popnSq | -9458 | 74.51 | 2.80 | 1.629 | -0.003 | 0.000 |
| logpopnSq | -11083 | 58.47 | 3.92 | 1.702 | -0.724 | 0.109 |

1. Poacea

|  | Dev | DeltaDev | phi | Intercept | X | X^2^ |
| --- | --- | --- | --- | --- | --- | --- |
| Null | -25279 | 0.00 | 1.65 | 1.935 | - | - |
| elev | -26010 | 731.28 | 1.89 | 1.466 | 0.002 | - |
| slope | -25806 | 526.70 | 1.81 | 1.447 | 0.150 | - |
| road | -26394 | 1115.15 | 2.09 | 2.726 | -3.459 | - |
| path | -25329 | 49.80 | 1.66 | 2.061 | -0.928 | - |
| popn | -25398 | 118.68 | 1.69 | 1.985 | -0.002 | - |
| logpopn | -26120 | 840.47 | 1.96 | 2.093 | -0.557 | - |
| elevSq | -26051 | 40.59 | 1.90 | 1.332 | 0.003 | 0.000 |
| slopeSq | -25871 | 65.07 | 1.83 | 1.272 | 0.247 | -0.008 |
| roadSq | -26441 | 46.39 | 2.10 | 2.893 | -5.211 | 3.099 |
| pathSq | -25340 | 11.01 | 1.67 | 2.123 | -1.948 | 2.464 |
| popnSq | -25497 | 99.36 | 1.71 | 2.022 | -0.004 | 0.000 |
| logpopnSq | -26166 | 46.85 | 2.00 | 2.177 | -0.542 | -0.105 |

**O_0.5_ = 20**

1. Araneae

|  | Dev | DeltaDev | phi | Intercept | X | X^2^ |
| --- | --- | --- | --- | --- | --- | --- |
| Null | -93319 | 0.00 | 5.66 | 4.119 | - | - |
| elev | -93545 | 226.73 | 5.98 | 3.867 | 0.001 | - |
| slope | -93390 | 71.82 | 5.78 | 3.950 | 0.052 | - |
| road | -93746 | 427.17 | 6.28 | 4.601 | -2.040 | - |
| path | -93358 | 39.45 | 5.73 | 4.227 | -0.763 | - |
| popn | -93437 | 118.72 | 5.71 | 4.151 | -0.001 | - |
| logpopn | -93727 | 408.36 | 6.26 | 4.236 | -0.376 | - |
| elevSq | -93642 | 97.13 | 6.03 | 3.700 | 0.002 | 0.000 |
| slopeSq | -93410 | 19.79 | 5.79 | 3.848 | 0.106 | -0.004 |
| roadSq | -93762 | 16.43 | 6.29 | 4.515 | -1.017 | -1.838 |
| pathSq | -93360 | 1.79 | 5.72 | 4.236 | -1.165 | 0.740 |
| popnSq | -93619 | 182.12 | 6.27 | 4.261 | -0.004 | 0.000 |
| logpopnSq | -93876 | 148.61 | 6.56 | 4.397 | -0.351 | -0.174 |

1. Coleaoptera

|  | Dev | DeltaDev | phi | Intercept | X | X^2^ |
| --- | --- | --- | --- | --- | --- | --- |
| Null | -24941 | 0.00 | 5.51 | 2.809 | - | - |
| elev | -26607 | 1665.96 | 7.78 | 2.224 | 0.002 | - |
| slope | -25379 | 437.72 | 5.97 | 2.461 | 0.108 | - |
| road | -27023 | 2082.44 | 8.67 | 3.747 | -3.771 | - |
| path | -25138 | 196.72 | 5.76 | 3.039 | -1.619 | - |
| popn | -25032 | 90.71 | 5.58 | 2.836 | -0.001 | - |
| logpopn | -27265 | 2324.38 | 9.16 | 3.112 | -0.778 | - |
| elevSq | -26614 | 6.99 | 7.81 | 2.182 | 0.003 | 0.000 |
| slopeSq | -25383 | 4.83 | 6.03 | 2.515 | 0.082 | 0.003 |
| roadSq | -27582 | 558.94 | 9.81 | 4.289 | -9.302 | 9.333 |
| pathSq | -25192 | 53.88 | 5.78 | 3.139 | -3.570 | 4.592 |
| popnSq | -25179 | 147.84 | 5.86 | 2.888 | -0.003 | 0.000 |
| logpopnSq | -27318 | 52.24 | 9.28 | 3.055 | -0.833 | 0.092 |

1. Opilionidae

|  | Dev | DeltaDev | phi | Intercept | X | X^2^ |
| --- | --- | --- | --- | --- | --- | --- |
| Null | -120975 | 0 | 25.28 | 5.750 | - | - |
| elev | -120986 | 10.85 | 26.06 | 5.716 | 0.000 | - |
| slope | -120979 | 3.99 | 25.58 | 5.721 | 0.013 | - |
| road | -120997 | 22.06 | 25.54 | 5.868 | -0.441 | - |
| path | -120975 | 0.48 | 25.81 | 5.777 | -0.112 | - |
| popn | -121046 | 71.08 | 26.74 | 5.811 | -0.001 | - |
| logpopn | -120998 | 23.78 | 25.73 | 5.780 | -0.082 | - |
| elevSq | -120992 | 6.44 | 25.69 | 5.639 | 0.001 | 0.000 |
| slopeSq | -120980 | 1.36 | 25.44 | 5.681 | 0.027 | -0.001 |
| roadSq | -120998 | 1.64 | 25.60 | 5.820 | -0.010 | -0.710 |
| pathSq | -120974 | -1.40 | 25.97 | 5.793 | -0.257 | 0.226 |
| popnSq | -121055 | 9.18 | 26.74 | 5.825 | -0.001 | 0.000 |
| logpopnSq | -121016 | 17.86 | 25.83 | 5.827 | -0.078 | -0.066 |

1. Odonata

|  | Dev | DeltaDev | phi | Intercept | X | X^2^ |
| --- | --- | --- | --- | --- | --- | --- |
| Null | -63352 | 0.00 | 2.77 | 3.125 | - | - |
| elev | -64202 | 850.03 | 3.29 | 2.628 | 0.002 | - |
| slope | -63593 | 240.81 | 2.91 | 2.808 | 0.097 | - |
| road | -64785 | 1433.48 | 3.95 | 4.091 | -3.885 | - |
| path | -63427 | 74.78 | 2.79 | 3.275 | -1.078 | - |
| popn | -63599 | 247.38 | 2.95 | 3.216 | -0.003 | - |
| logpopn | -64608 | 1255.73 | 3.68 | 3.377 | -0.689 | - |
| elevSq | -64394 | 192.25 | 3.39 | 2.389 | 0.004 | 0.000 |
| slopeSq | -63628 | 35.65 | 2.95 | 2.681 | 0.168 | -0.006 |
| roadSq | -64791 | 5.27 | 3.97 | 4.045 | -3.295 | -1.141 |
| pathSq | -63430 | 3.52 | 2.79 | 3.308 | -1.598 | 1.274 |
| popnSq | -63770 | 170.45 | 3.13 | 3.276 | -0.005 | 0.000 |
| logpopnSq | -64761 | 153.22 | 3.86 | 3.529 | -0.633 | -0.179 |

1. Papilionoidea

|  | Dev | DeltaDev | phi | Intercept | X | X^2^ |
| --- | --- | --- | --- | --- | --- | --- |
| Null | -43945 | 0.00 | 1.94 | 2.503 | - | - |
| elev | -45317 | 1371.88 | 2.60 | 1.838 | 0.003 | - |
| slope | -44220 | 275.57 | 2.06 | 2.149 | 0.109 | - |
| road | -46425 | 2480.43 | 3.49 | 3.820 | -5.238 | - |
| path | -44119 | 173.94 | 2.01 | 2.737 | -1.723 | - |
| popn | -44275 | 330.10 | 2.18 | 2.636 | -0.004 | - |
| logpopn | -46420 | 2475.81 | 3.49 | 2.930 | -1.037 | - |
| elevSq | -45799 | 482.37 | 2.92 | 1.463 | 0.006 | 0.000 |
| slopeSq | -44290 | 69.64 | 2.09 | 1.965 | 0.209 | -0.008 |
| roadSq | -46499 | 73.83 | 3.55 | 4.035 | -7.426 | 3.929 |
| pathSq | -44124 | 5.14 | 2.01 | 2.776 | -2.376 | 1.690 |
| popnSq | -44493 | 218.42 | 2.27 | 2.697 | -0.007 | 0.000 |
| logpopnSq | -46598 | 177.80 | 3.59 | 3.070 | -0.941 | -0.219 |

1. Bryophyta

|  | Dev | DeltaDev | phi | Intercept | X | X^2^ |
| --- | --- | --- | --- | --- | --- | --- |
| Null | -30790 | 0.00 | 3.42 | 2.666 | - | - |
| elev | -31953 | 1163.28 | 4.24 | 2.157 | 0.002 | - |
| slope | -31478 | 688.33 | 3.85 | 2.185 | 0.150 | - |
| road | -32344 | 1554.43 | 4.71 | 3.522 | -3.548 | - |
| path | -30873 | 83.68 | 3.46 | 2.809 | -1.110 | - |
| popn | -30828 | 38.53 | 3.43 | 2.677 | -0.001 | - |
| logpopn | -31911 | 1121.60 | 4.23 | 2.821 | -0.535 | - |
| elevSq | -31958 | 5.01 | 4.22 | 2.208 | 0.001 | 0.000 |
| slopeSq | -31482 | 4.45 | 3.84 | 2.129 | 0.182 | -0.003 |
| roadSq | -32613 | 268.55 | 5.03 | 3.890 | -7.298 | 6.379 |
| pathSq | -30901 | 28.03 | 3.50 | 2.890 | -2.549 | 3.702 |
| popnSq | -30868 | 40.26 | 3.44 | 2.702 | -0.002 | 0.000 |
| logpopnSq | -31985 | 74.06 | 4.32 | 2.758 | -0.589 | 0.118 |

1. Fungi

|  | Dev | DeltaDev | phi | Intercept | X | X^2^ |
| --- | --- | --- | --- | --- | --- | --- |
| Null | -17610 | 0.00 | 5.26 | 2.433 | - | - |
| elev | -20039 | 2429.15 | 8.32 | 1.828 | 0.002 | - |
| slope | -18973 | 1363.60 | 6.76 | 1.872 | 0.176 | - |
| road | -19316 | 1705.76 | 7.37 | 3.149 | -3.067 | - |
| path | -17773 | 162.82 | 5.42 | 2.619 | -1.408 | - |
| popn | -17641 | 31.44 | 5.29 | 2.446 | 0.000 | - |
| logpopn | -19491 | 1881.46 | 7.58 | 2.612 | -0.590 | - |
| elevSq | -20633 | 594.24 | 9.54 | 2.255 | -0.002 | 0.000 |
| slopeSq | -19053 | 80.05 | 6.92 | 2.084 | 0.053 | 0.013 |
| roadSq | -20735 | 1419.47 | 9.89 | 3.937 | -11.729 | 15.501 |
| pathSq | -17879 | 106.77 | 5.57 | 2.784 | -4.085 | 6.120 |
| popnSq | -17690 | 48.52 | 5.36 | 2.468 | -0.002 | 0.000 |
| logpopnSq | -20103 | 611.47 | 8.66 | 2.420 | -0.726 | 0.325 |

1. Lichen

|  | Dev | DeltaDev | phi | Intercept | X | X^2^ |
| --- | --- | --- | --- | --- | --- | --- |
| Null | -22335 | 0.00 | 3.42 | 2.390 | - | - |
| elev | -23770 | 1435.39 | 4.41 | 1.877 | 0.002 | - |
| slope | -23086 | 751.11 | 3.90 | 1.950 | 0.136 | - |
| road | -23861 | 1526.28 | 4.65 | 3.144 | -3.279 | - |
| path | -22476 | 141.14 | 3.53 | 2.582 | -1.403 | - |
| popn | -22350 | 15.46 | 3.43 | 2.400 | 0.000 | - |
| logpopn | -23538 | 1203.28 | 4.26 | 2.528 | -0.508 | - |
| elevSq | -24113 | 343.13 | 4.73 | 2.227 | -0.001 | 0.000 |
| slopeSq | -23160 | 73.58 | 4.00 | 2.168 | 0.012 | 0.012 |
| roadSq | -24576 | 714.39 | 5.33 | 3.721 | -9.486 | 10.817 |
| pathSq | -22533 | 56.57 | 3.55 | 2.695 | -3.398 | 4.560 |
| popnSq | -22361 | 10.45 | 3.44 | 2.409 | -0.001 | 0.000 |
| logpopnSq | -23988 | 450.24 | 4.73 | 2.349 | -0.630 | 0.289 |

1. Amphibia

|  | Dev | DeltaDev | phi | Intercept | X | X^2^ |
| --- | --- | --- | --- | --- | --- | --- |
| Null | -89519 | 0.00 | 2.35 | 3.285 | - | - |
| elev | -89807 | 288.09 | 2.48 | 2.974 | 0.001 | - |
| slope | -89609 | 89.47 | 2.40 | 3.078 | 0.060 | - |
| road | -90011 | 491.38 | 2.63 | 3.801 | -2.220 | - |
| path | -89532 | 12.97 | 2.37 | 3.344 | -0.460 | - |
| popn | -89738 | 219.19 | 2.55 | 3.387 | -0.002 | - |
| logpopn | -90013 | 493.49 | 2.64 | 3.418 | -0.422 | - |
| elevSq | -89929 | 121.90 | 2.58 | 2.785 | 0.003 | 0.000 |
| slopeSq | -89639 | 29.79 | 2.42 | 2.953 | 0.126 | -0.005 |
| roadSq | -90026 | 14.91 | 2.67 | 3.732 | -1.161 | -1.914 |
| pathSq | -89534 | 1.77 | 2.37 | 3.372 | -1.012 | 1.094 |
| popnSq | -89834 | 95.48 | 2.64 | 3.429 | -0.004 | 0.000 |
| logpopnSq | -90165 | 152.04 | 2.74 | 3.578 | -0.393 | -0.181 |

1. Aves

|  | Dev | DeltaDev | phi | Intercept | X | X^2^ |
| --- | --- | --- | --- | --- | --- | --- |
| Null | -3943 | 0.00 | 1.78 | 0.906 | - | - |
| elev | -5703 | 1759.95 | 2.63 | 0.112 | 0.003 | - |
| slope | -4606 | 663.45 | 2.03 | 0.346 | 0.171 | - |
| road | -6575 | 2632.37 | 3.20 | 2.116 | -5.332 | - |
| path | -4154 | 211.22 | 1.87 | 1.173 | -1.944 | - |
| popn | -4453 | 510.14 | 2.08 | 1.047 | -0.005 | - |
| logpopn | -7056 | 3113.08 | 3.69 | 1.276 | -1.132 | - |
| elevSq | -5986 | 283.31 | 2.79 | -0.201 | 0.006 | 0.000 |
| slopeSq | -4660 | 54.19 | 2.07 | 0.146 | 0.277 | -0.009 |
| roadSq | -6783 | 207.78 | 3.32 | 2.416 | -8.952 | 6.443 |
| pathSq | -4181 | 26.68 | 1.88 | 1.252 | -3.555 | 3.451 |
| popnSq | -4721 | 268.53 | 2.19 | 1.106 | -0.008 | 0.000 |
| logpopnSq | -7263 | 207.24 | 3.84 | 1.430 | -1.079 | -0.223 |

1. Land mammals

|  | Dev | DeltaDev | phi | Intercept | X | X^2^ |
| --- | --- | --- | --- | --- | --- | --- |
| Null | -58762 | 0.00 | 3.200 | 3.186 | - | - |
| elev | -59087 | 324.96 | 3.420 | 2.879 | 0.001 | - |
| slope | -58898 | 135.60 | 3.270 | 2.946 | 0.070 | - |
| road | -59529 | 766.10 | 3.760 | 3.808 | -2.671 | - |
| path | -58841 | 78.65 | 3.230 | 3.324 | -1.107 | - |
| popn | -58941 | 178.58 | 3.320 | 3.248 | -0.002 | - |
| logpopn | -59392 | 629.11 | 3.610 | 3.312 | -0.450 | - |
| elevSq | -59125 | 37.94 | 3.410 | 2.764 | 0.002 | 0 |
| slopeSq | -58909 | 11.21 | 3.260 | 2.871 | 0.113 | -0.00351 |
| roadSq | -59529 | 0.23 | 3.750 | 3.824 | -2.957 | 0.44615 |
| pathSq | -58843 | 1.49 | 3.240 | 3.351 | -1.598 | 1.00465 |
| popnSq | -59053 | 111.98 | 3.460 | 3.290 | -0.004 | 0 |
| logpopnSq | -59426 | 34.53 | 3.670 | 3.387 | -0.435 | -0.08441 |

1. Vascular plants

|  | Dev | DeltaDev | phi | Intercept | X | X^2^ |
| --- | --- | --- | --- | --- | --- | --- |
| Null | -14299 | 0.00 | 4.36 | 2.135 | - | - |
| elev | -15925 | 1625.63 | 6.03 | 1.564 | 0.002 | - |
| slope | -15077 | 777.90 | 5.07 | 1.654 | 0.152 | - |
| road | -16080 | 1780.33 | 6.29 | 2.951 | -3.445 | - |
| path | -14418 | 118.36 | 4.43 | 2.305 | -1.266 | - |
| popn | -14362 | 62.62 | 4.38 | 2.155 | -0.001 | - |
| logpopn | -16016 | 1716.36 | 6.21 | 2.342 | -0.643 | - |
| elevSq | -15947 | 22.38 | 6.08 | 1.643 | 0.001 | 0.000 |
| slopeSq | -15077 | -0.55 | 5.06 | 1.644 | 0.163 | -0.001 |
| roadSq | -16707 | 627.40 | 7.24 | 3.485 | -8.922 | 9.304 |
| pathSq | -14454 | 36.61 | 4.48 | 2.415 | -2.978 | 4.043 |
| popnSq | -14447 | 84.85 | 4.47 | 2.193 | -0.002 | 0.000 |
| logpopnSq | -16085 | 69.13 | 6.34 | 2.278 | -0.694 | 0.105 |

1. Poacea

|  | Dev | DeltaDev | phi | Intercept | X | X^2^ |
| --- | --- | --- | --- | --- | --- | --- |
| Null | -30599 | 0.00 | 2.55 | 2.435 | - | - |
| elev | -31330 | 730.94 | 2.93 | 1.985 | 0.002 | - |
| slope | -31126 | 527.42 | 2.81 | 1.966 | 0.145 | - |
| road | -31710 | 1111.32 | 3.25 | 3.199 | -3.263 | - |
| path | -30649 | 49.85 | 2.57 | 2.549 | -0.879 | - |
| popn | -30707 | 108.03 | 2.59 | 2.469 | -0.002 | - |
| logpopn | -31446 | 847.45 | 3.07 | 2.603 | -0.539 | - |
| elevSq | -31370 | 39.95 | 2.98 | 1.860 | 0.003 | 0.000 |
| slopeSq | -31187 | 60.95 | 2.84 | 1.790 | 0.240 | -0.008 |
| roadSq | -31769 | 58.66 | 3.32 | 3.374 | -5.069 | 3.124 |
| pathSq | -30660 | 11.16 | 2.58 | 2.606 | -1.911 | 2.202 |
| popnSq | -30814 | 107.16 | 2.69 | 2.515 | -0.003 | 0.000 |
| logpopnSq | -31488 | 42.18 | 3.08 | 2.673 | -0.516 | -0.090 |

**O_0.5_ = 50**

1. Araneae

|  | Dev | DeltaDev | phi | Intercept | X | X^2^ |
| --- | --- | --- | --- | --- | --- | --- |
| Null | -101998 | 0.00 | 13.05 | 4.971 | - | - |
| elev | -102225 | 226.37 | 13.52 | 4.717 | 0.001 | - |
| slope | -102070 | 71.91 | 13.23 | 4.806 | 0.052 | - |
| road | -102425 | 426.29 | 14.33 | 5.430 | -1.996 | - |
| path | -102038 | 39.36 | 13.00 | 5.068 | -0.772 | - |
| popn | -102109 | 110.72 | 13.46 | 4.994 | -0.001 | - |
| logpopn | -102406 | 408.03 | 14.24 | 5.074 | -0.373 | - |
| elevSq | -102321 | 96.87 | 13.87 | 4.523 | 0.002 | 0.000 |
| slopeSq | -102089 | 19.35 | 13.23 | 4.713 | 0.106 | -0.004 |
| roadSq | -102440 | 15.23 | 14.61 | 5.359 | -1.121 | -1.756 |
| pathSq | -102039 | 1.40 | 13.16 | 5.095 | -1.169 | 1.013 |
| popnSq | -102295 | 186.28 | 14.59 | 5.113 | -0.004 | 0.000 |
| logpopnSq | -102554 | 147.91 | 15.10 | 5.241 | -0.345 | -0.175 |

1. Coleaoptera

|  | Dev | DeltaDev | phi | Intercept | X | X^2^ |
| --- | --- | --- | --- | --- | --- | --- |
| Null | -33208 | 0.00 | 12.41 | 3.663 | - | - |
| elev | -34878 | 1670.21 | 17.83 | 3.107 | 0.002 | - |
| slope | -33645 | 436.91 | 13.53 | 3.324 | 0.105 | - |
| road | -35277 | 2068.81 | 19.87 | 4.571 | -3.576 | - |
| path | -33405 | 197.20 | 13.01 | 3.875 | -1.546 | - |
| popn | -33291 | 82.91 | 12.61 | 3.677 | -0.001 | - |
| logpopn | -35534 | 2325.74 | 20.98 | 3.978 | -0.747 | - |
| elevSq | -34884 | 5.80 | 17.74 | 3.063 | 0.003 | 0.000 |
| slopeSq | -33651 | 5.85 | 13.48 | 3.388 | 0.070 | 0.003 |
| roadSq | -35861 | 583.74 | 22.69 | 5.140 | -9.042 | 9.070 |
| pathSq | -33459 | 53.44 | 13.06 | 3.999 | -3.466 | 4.412 |
| popnSq | -33446 | 155.04 | 13.21 | 3.738 | -0.003 | 0.000 |
| logpopnSq | -35598 | 64.26 | 21.48 | 3.920 | -0.821 | 0.097 |

1. Opilionidae

|  | Dev | DeltaDev | phi | Intercept | X | X^2^ |
| --- | --- | --- | --- | --- | --- | --- |
| Null | -129779 | 0.00 | 60.65 | 6.631 | - | - |
| elev | -129789 | 10.47 | 60.56 | 6.566 | 0.000 | - |
| slope | -129783 | 4.03 | 61.11 | 6.588 | 0.012 | - |
| road | -129801 | 21.96 | 61.41 | 6.735 | -0.429 | - |
| path | -129779 | 0.35 | 60.78 | 6.646 | -0.130 | - |
| popn | -129849 | 70.61 | 63.05 | 6.683 | -0.001 | - |
| logpopn | -129802 | 23.56 | 60.32 | 6.657 | -0.086 | - |
| elevSq | -129795 | 6.27 | 61.06 | 6.538 | 0.001 | 0.000 |
| slopeSq | -129784 | 1.35 | 60.69 | 6.561 | 0.029 | -0.001 |
| roadSq | -129802 | 1.77 | 61.22 | 6.696 | -0.122 | -0.598 |
| pathSq | -129778 | -0.77 | 60.99 | 6.654 | -0.247 | 0.177 |
| popnSq | -129859 | 9.25 | 63.91 | 6.693 | -0.001 | 0.000 |
| logpopnSq | -129821 | 18.43 | 62.45 | 6.702 | -0.075 | -0.064 |

1. Odonata

|  | Dev | DeltaDev | phi | Intercept | X | X^2^ |
| --- | --- | --- | --- | --- | --- | --- |
| Null | -71709 | 0.00 | 5.77 | 3.890 | - | - |
| elev | -72556 | 847.13 | 6.92 | 3.423 | 0.002 | - |
| slope | -71949 | 240.01 | 6.09 | 3.591 | 0.094 | - |
| road | -73143 | 1434.44 | 8.39 | 4.880 | -3.817 | - |
| path | -71783 | 74.30 | 5.88 | 4.036 | -1.038 | - |
| popn | -71872 | 163.20 | 5.81 | 3.910 | -0.002 | - |
| logpopn | -72963 | 1254.03 | 7.84 | 4.152 | -0.672 | - |
| elevSq | -72744 | 188.05 | 7.37 | 3.189 | 0.004 | 0.000 |
| slopeSq | -71983 | 34.88 | 6.10 | 3.462 | 0.161 | -0.006 |
| roadSq | -73151 | 7.72 | 8.48 | 4.815 | -3.078 | -1.246 |
| pathSq | -71787 | 3.68 | 5.87 | 4.076 | -1.655 | 1.273 |
| popnSq | -72111 | 239.38 | 6.57 | 4.048 | -0.005 | 0.000 |
| logpopnSq | -73104 | 141.32 | 8.25 | 4.289 | -0.612 | -0.160 |

1. Papilionoidea

|  | Dev | DeltaDev | phi | Intercept | X | X^2^ |
| --- | --- | --- | --- | --- | --- | --- |
| Null | -51825 | 0.00 | 3.68 | 3.187 | - | - |
| elev | -53184 | 1359.07 | 5.00 | 2.570 | 0.002 | - |
| slope | -52098 | 272.78 | 3.92 | 2.859 | 0.105 | - |
| road | -54251 | 2426.30 | 6.74 | 4.491 | -4.868 | - |
| path | -51996 | 171.03 | 3.80 | 3.417 | -1.637 | - |
| popn | -52103 | 277.87 | 3.92 | 3.276 | -0.003 | - |
| logpopn | -54268 | 2442.87 | 6.73 | 3.645 | -0.988 | - |
| elevSq | -53645 | 461.25 | 5.55 | 2.229 | 0.006 | 0.000 |
| slopeSq | -52165 | 67.44 | 3.94 | 2.684 | 0.205 | -0.008 |
| roadSq | -54334 | 82.93 | 6.83 | 4.705 | -7.126 | 3.565 |
| pathSq | -52001 | 4.89 | 3.81 | 3.454 | -2.260 | 1.603 |
| popnSq | -52333 | 230.08 | 4.29 | 3.370 | -0.006 | 0.000 |
| logpopnSq | -54381 | 112.80 | 6.70 | 3.730 | -0.887 | -0.149 |

1. Bryophyta

|  | Dev | DeltaDev | phi | Intercept | X | X^2^ |
| --- | --- | --- | --- | --- | --- | --- |
| Null | -38923 | 0.00 | 7.31 | 3.472 | - | - |
| elev | -40083 | 1159.98 | 9.20 | 2.997 | 0.002 | - |
| slope | -39610 | 686.77 | 8.34 | 3.009 | 0.144 | - |
| road | -40461 | 1538.05 | 10.24 | 4.298 | -3.339 | - |
| path | -39007 | 83.66 | 7.47 | 3.617 | -1.059 | - |
| popn | -38960 | 36.82 | 7.36 | 3.479 | -0.001 | - |
| logpopn | -40038 | 1114.42 | 9.22 | 3.641 | -0.513 | - |
| elevSq | -40089 | 5.58 | 9.28 | 3.040 | 0.001 | 0.000 |
| slopeSq | -39613 | 3.02 | 8.32 | 2.980 | 0.171 | -0.002 |
| roadSq | -40754 | 292.22 | 11.06 | 4.700 | -7.201 | 6.261 |
| pathSq | -39035 | 28.14 | 7.50 | 3.696 | -2.513 | 3.209 |
| popnSq | -39001 | 41.39 | 7.43 | 3.511 | -0.002 | 0.000 |
| logpopnSq | -40121 | 82.93 | 9.37 | 3.569 | -0.573 | 0.117 |

1. Fungi

|  | Dev | DeltaDev | phi | Intercept | X | X^2^ |
| --- | --- | --- | --- | --- | --- | --- |
| Null | -25607 | 0.00 | 11.59 | 3.273 | - | - |
| elev | -28009 | 2402.28 | 18.47 | 2.718 | 0.002 | - |
| slope | -26956 | 1349.11 | 14.93 | 2.757 | 0.169 | - |
| road | -27276 | 1669.18 | 16.35 | 3.958 | -2.845 | - |
| path | -25769 | 162.70 | 11.98 | 3.453 | -1.351 | - |
| popn | -25637 | 30.50 | 11.72 | 3.285 | 0.000 | - |
| logpopn | -27461 | 1854.61 | 16.77 | 3.469 | -0.559 | - |
| elevSq | -28621 | 612.41 | 21.15 | 3.127 | -0.002 | 0.000 |
| slopeSq | -27047 | 90.97 | 15.36 | 2.958 | 0.045 | 0.013 |
| roadSq | -28716 | 1440.27 | 22.10 | 4.756 | -11.210 | 14.902 |
| pathSq | -25876 | 106.80 | 12.23 | 3.606 | -3.901 | 5.877 |
| popnSq | -25686 | 49.01 | 11.78 | 3.314 | -0.002 | 0.000 |
| logpopnSq | -28098 | 637.12 | 19.27 | 3.280 | -0.714 | 0.316 |

1. Lichen

|  | Dev | DeltaDev | phi | Intercept | X | X^2^ |
| --- | --- | --- | --- | --- | --- | --- |
| Null | -30243 | 0.00 | 7.22 | 3.186 | - | - |
| elev | -31658 | 1414.50 | 9.42 | 2.720 | 0.002 | - |
| slope | -30984 | 740.29 | 8.24 | 2.786 | 0.129 | - |
| road | -31731 | 1487.25 | 9.84 | 3.917 | -3.010 | - |
| path | -30384 | 140.53 | 7.44 | 3.379 | -1.327 | - |
| popn | -30259 | 15.22 | 7.27 | 3.199 | 0.000 | - |
| logpopn | -31422 | 1178.58 | 9.06 | 3.342 | -0.475 | - |
| elevSq | -32014 | 356.41 | 10.13 | 3.067 | -0.001 | 0.000 |
| slopeSq | -31066 | 82.24 | 8.51 | 2.996 | 0.007 | 0.012 |
| roadSq | -32469 | 737.97 | 11.47 | 4.511 | -9.032 | 10.183 |
| pathSq | -30440 | 56.17 | 7.49 | 3.496 | -3.333 | 4.529 |
| popnSq | -30269 | 10.41 | 7.21 | 3.214 | -0.001 | 0.000 |
| logpopnSq | -31891 | 469.60 | 9.98 | 3.178 | -0.608 | 0.286 |

1. Amphibia

|  | Dev | DeltaDev | phi | Intercept | X | X^2^ |
| --- | --- | --- | --- | --- | --- | --- |
| Null | -97896 | 0.00 | 4.50 | 3.959 | - | - |
| elev | -98179 | 283.01 | 4.74 | 3.678 | 0.001 | - |
| slope | -97983 | 87.82 | 4.56 | 3.766 | 0.058 | - |
| road | -98377 | 481.15 | 5.04 | 4.468 | -2.162 | - |
| path | -97908 | 12.80 | 4.56 | 4.018 | -0.451 | - |
| popn | -98088 | 192.00 | 4.72 | 4.032 | -0.002 | - |
| logpopn | -98380 | 484.20 | 5.03 | 4.085 | -0.414 | - |
| elevSq | -98298 | 119.12 | 4.93 | 3.464 | 0.003 | 0.000 |
| slopeSq | -98013 | 29.14 | 4.63 | 3.641 | 0.126 | -0.005 |
| roadSq | -98391 | 14.56 | 5.08 | 4.401 | -1.118 | -1.876 |
| pathSq | -97910 | 1.77 | 4.49 | 4.039 | -0.884 | 1.183 |
| popnSq | -98198 | 110.66 | 4.99 | 4.096 | -0.003 | 0.000 |
| logpopnSq | -98526 | 146.06 | 5.30 | 4.253 | -0.378 | -0.172 |

1. Aves

|  | Dev | DeltaDev | phi | Intercept | X | X^2^ |
| --- | --- | --- | --- | --- | --- | --- |
| Null | -8616 | 0.00 | 2.45 | 1.474 | - | - |
| elev | -10194 | 1578.10 | 3.49 | 0.778 | 0.003 | - |
| slope | -9208 | 591.79 | 2.76 | 0.973 | 0.154 | - |
| road | -11068 | 2451.76 | 4.23 | 2.605 | -4.775 | - |
| path | -8812 | 195.90 | 2.55 | 1.714 | -1.781 | - |
| popn | -9248 | 631.72 | 2.99 | 1.651 | -0.006 | - |
| logpopn | -11583 | 2966.64 | 5.05 | 1.898 | -1.085 | - |
| elevSq | -10474 | 280.25 | 3.70 | 0.498 | 0.005 | 0.000 |
| slopeSq | -9256 | 47.86 | 2.78 | 0.809 | 0.240 | -0.008 |
| roadSq | -11205 | 137.26 | 4.38 | 2.862 | -7.459 | 4.628 |
| pathSq | -8833 | 21.53 | 2.55 | 1.786 | -3.098 | 3.020 |
| popnSq | -9547 | 299.11 | 3.19 | 1.718 | -0.009 | 0.000 |
| logpopnSq | -11863 | 279.91 | 5.32 | 2.070 | -1.001 | -0.244 |

1. Land mammals

|  | Dev | DeltaDev | phi | Intercept | X | X^2^ |
| --- | --- | --- | --- | --- | --- | --- |
| Null | -67165 | 0.00 | 6.88 | 3.966 | - | - |
| elev | -67490 | 324.52 | 7.32 | 3.674 | 0.001 | - |
| slope | -67301 | 135.48 | 7.04 | 3.746 | 0.069 | - |
| road | -67930 | 764.93 | 8.27 | 4.604 | -2.615 | - |
| path | -67243 | 78.28 | 6.96 | 4.121 | -1.064 | - |
| popn | -67302 | 136.63 | 7.05 | 4.011 | -0.001 | - |
| logpopn | -67794 | 628.94 | 7.86 | 4.117 | -0.441 | - |
| elevSq | -67527 | 37.46 | 7.44 | 3.582 | 0.002 | 0.000 |
| slopeSq | -67311 | 10.47 | 7.08 | 3.676 | 0.105 | -0.003 |
| roadSq | -67930 | -0.24 | 8.16 | 4.631 | -2.842 | 0.423 |
| pathSq | -67245 | 1.94 | 7.01 | 4.151 | -1.534 | 1.008 |
| popnSq | -67449 | 147.33 | 7.41 | 4.089 | -0.003 | 0.000 |
| logpopnSq | -67827 | 32.74 | 8.01 | 4.189 | -0.423 | -0.078 |

1. Vascular plants

|  | Dev | DeltaDev | phi | Intercept | X | X^2^ |
| --- | --- | --- | --- | --- | --- | --- |
| Null | -21962 | 0.00 | 9.09 | 2.948 | - | - |
| elev | -23568 | 1605.75 | 12.75 | 2.424 | 0.002 | - |
| slope | -22731 | 768.80 | 10.66 | 2.494 | 0.146 | - |
| road | -23700 | 1737.56 | 13.18 | 3.712 | -3.137 | - |
| path | -22080 | 118.03 | 9.32 | 3.111 | -1.217 | - |
| popn | -22019 | 56.61 | 9.16 | 2.971 | -0.001 | - |
| logpopn | -23662 | 1699.70 | 13.11 | 3.168 | -0.607 | - |
| elevSq | -23593 | 25.11 | 12.86 | 2.505 | 0.001 | 0.000 |
| slopeSq | -22730 | -0.61 | 10.55 | 2.479 | 0.153 | 0.000 |
| roadSq | -24360 | 660.47 | 15.47 | 4.284 | -8.516 | 8.849 |
| pathSq | -22116 | 36.09 | 9.37 | 3.211 | -2.772 | 3.571 |
| popnSq | -22108 | 88.81 | 9.38 | 3.007 | -0.002 | 0.000 |
| logpopnSq | -23741 | 79.39 | 13.45 | 3.110 | -0.668 | 0.107 |

1. Poacea

|  | Dev | DeltaDev | phi | Intercept | X | X^2^ |
| --- | --- | --- | --- | --- | --- | --- |
| Null | -38477 | 0.00 | 5.11 | 3.182 | - | - |
| elev | -39203 | 725.56 | 5.95 | 2.755 | 0.002 | - |
| slope | -39002 | 524.44 | 5.66 | 2.742 | 0.141 | - |
| road | -39570 | 1092.89 | 6.53 | 3.911 | -3.031 | - |
| path | -38527 | 49.24 | 5.16 | 3.290 | -0.854 | - |
| popn | -38559 | 81.09 | 5.15 | 3.201 | -0.001 | - |
| logpopn | -39323 | 845.58 | 6.18 | 3.354 | -0.521 | - |
| elevSq | -39241 | 38.38 | 5.98 | 2.641 | 0.003 | 0.000 |
| slopeSq | -39059 | 56.83 | 5.76 | 2.583 | 0.224 | -0.008 |
| roadSq | -39643 | 72.36 | 6.72 | 4.112 | -4.946 | 3.166 |
| pathSq | -38538 | 10.99 | 5.18 | 3.351 | -1.836 | 2.300 |
| popnSq | -38686 | 127.20 | 5.36 | 3.258 | -0.003 | 0.000 |
| logpopnSq | -39359 | 36.16 | 6.24 | 3.417 | -0.496 | -0.080 |

**S1 File Figure A**

**Figure A**. **Logged number of observation maps and ignorance score maps for all 13 reference taxonomic groups.** **Logged number of** observations of each taxonomic group over Sweden for the period 2000-14 and ignorance maps produced with the half-ignorance algorithm (O_0.5_ = 1). Grid resolution is 10 x 10 km. The black contour shows a 10 km buffer around Sweden’s land surface. The inset shows the location of Sweden in Europe.


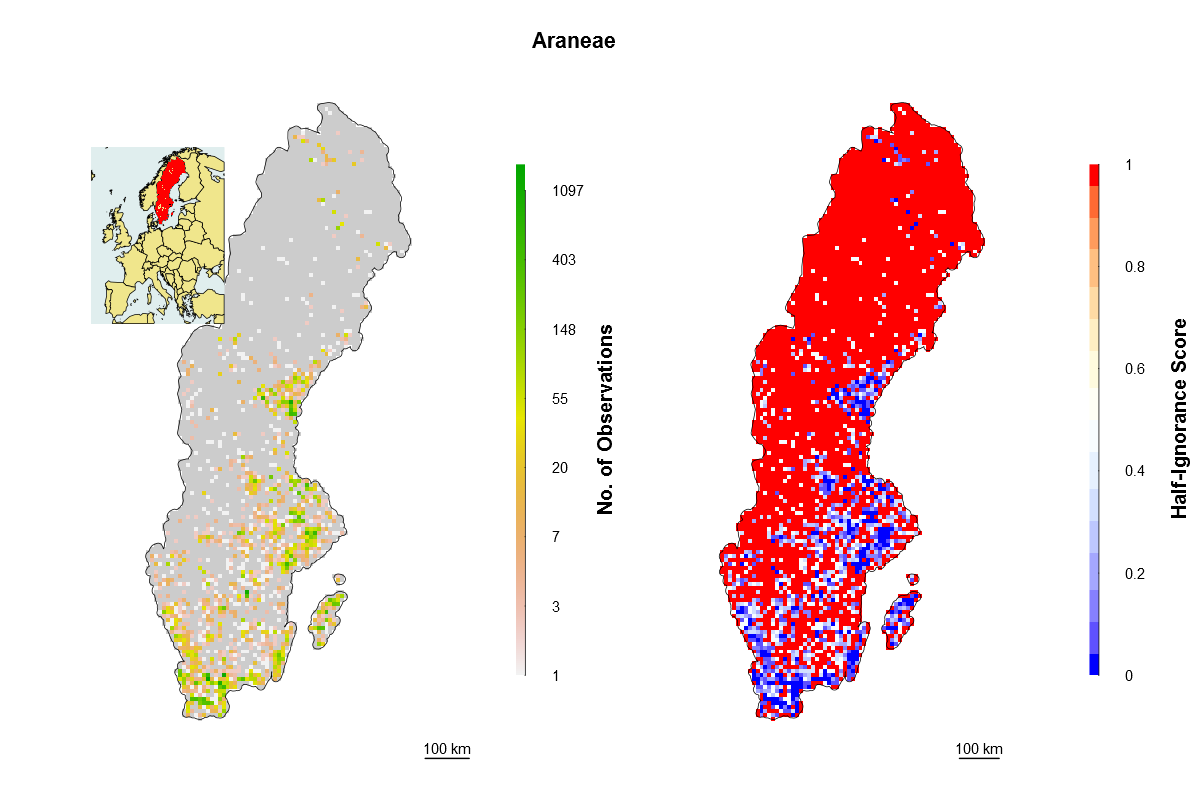


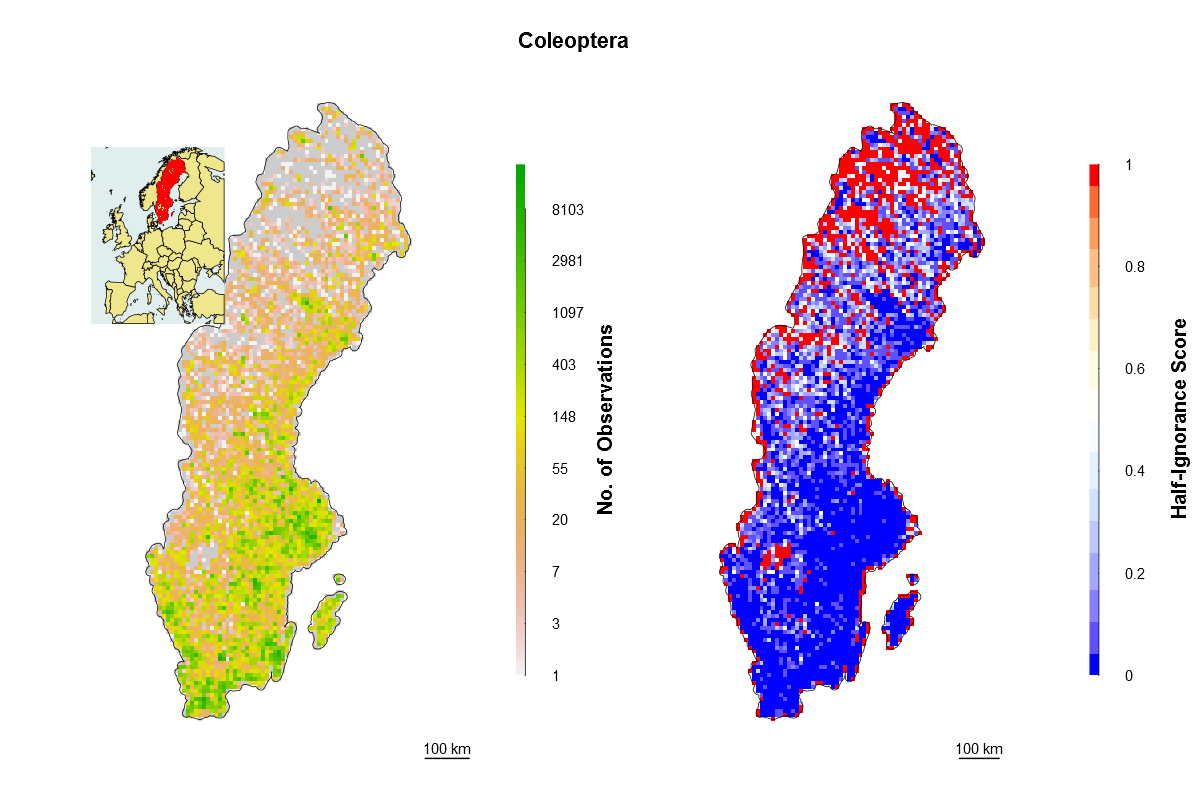


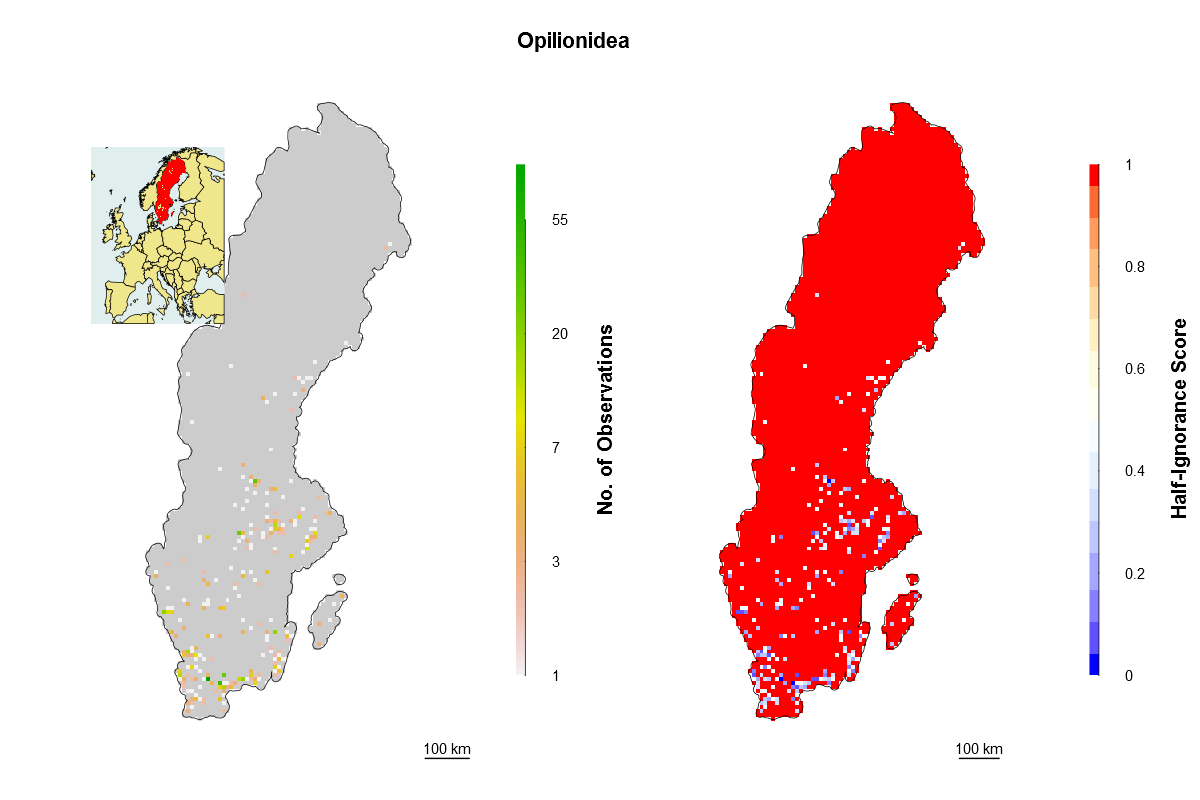


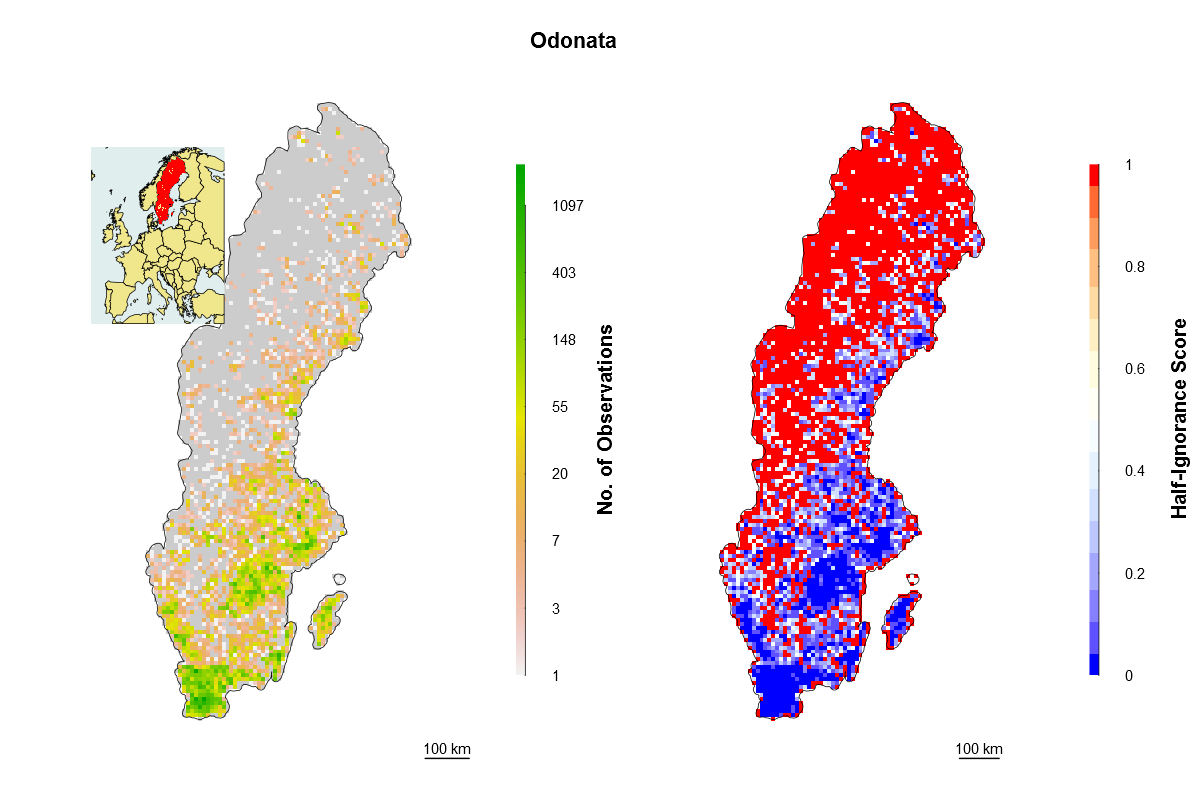


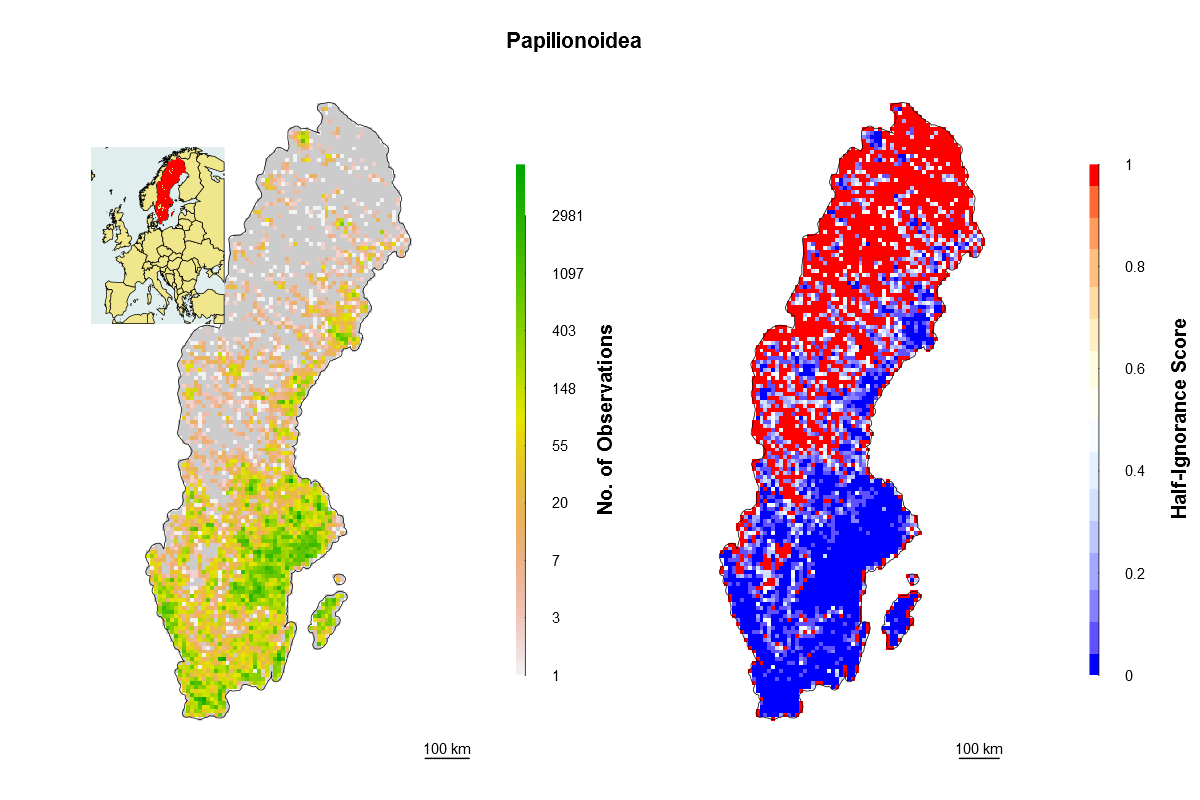


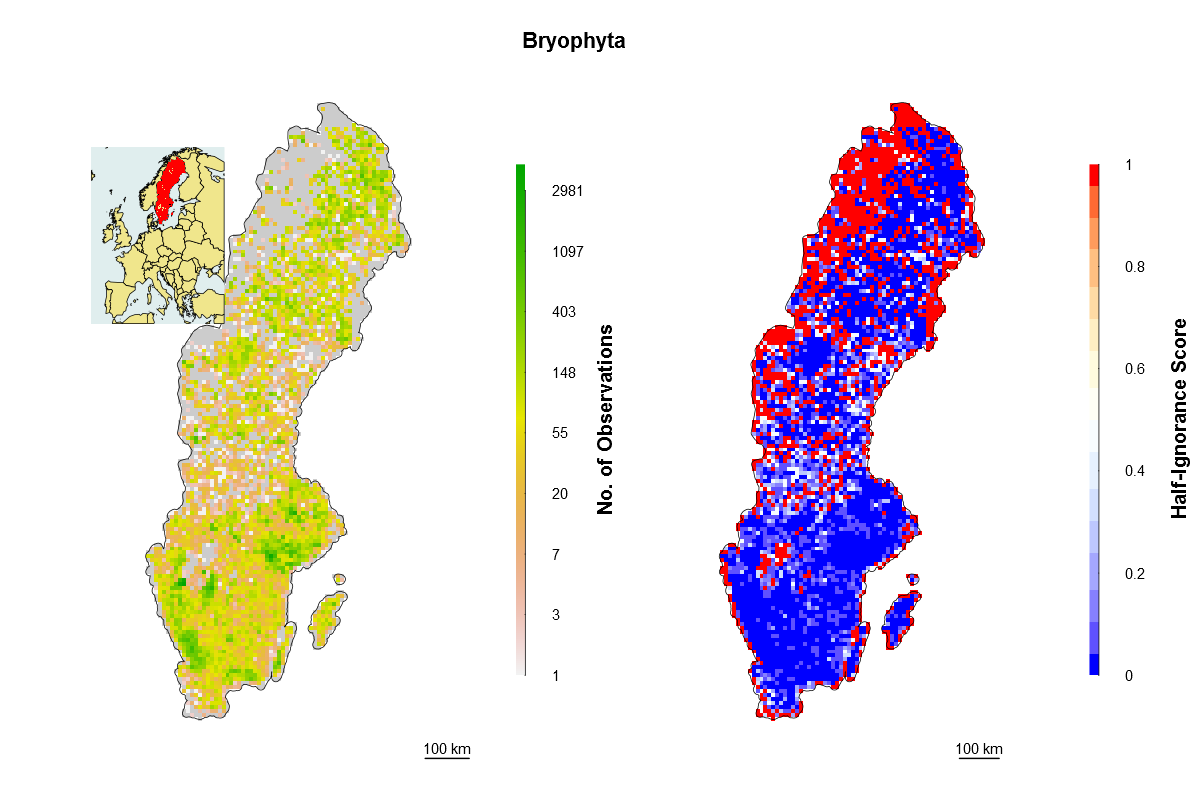


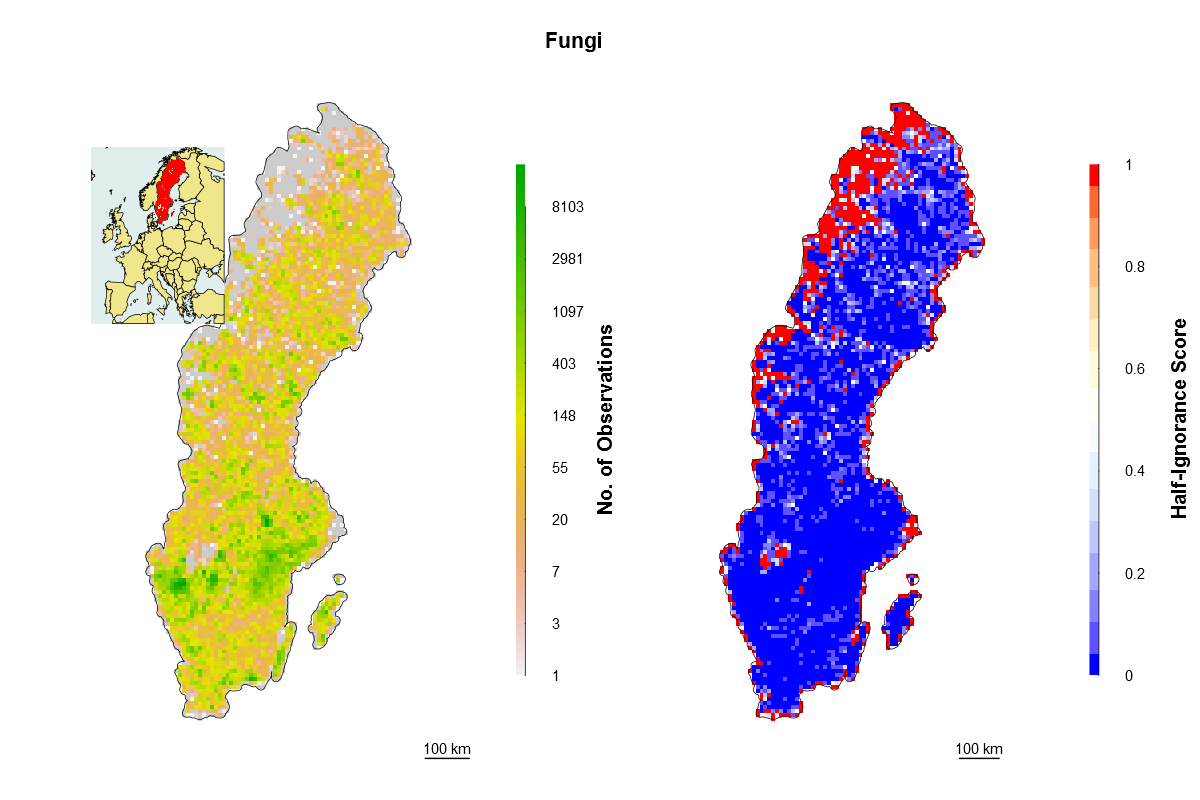


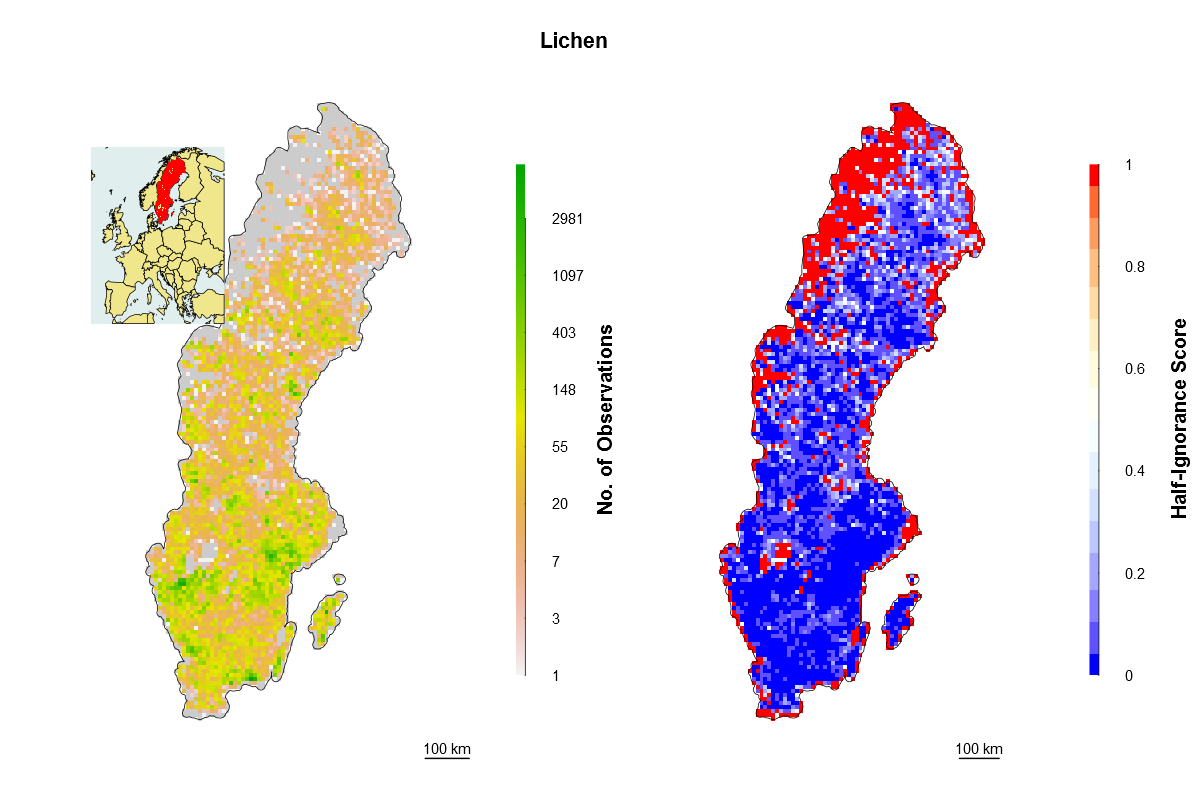


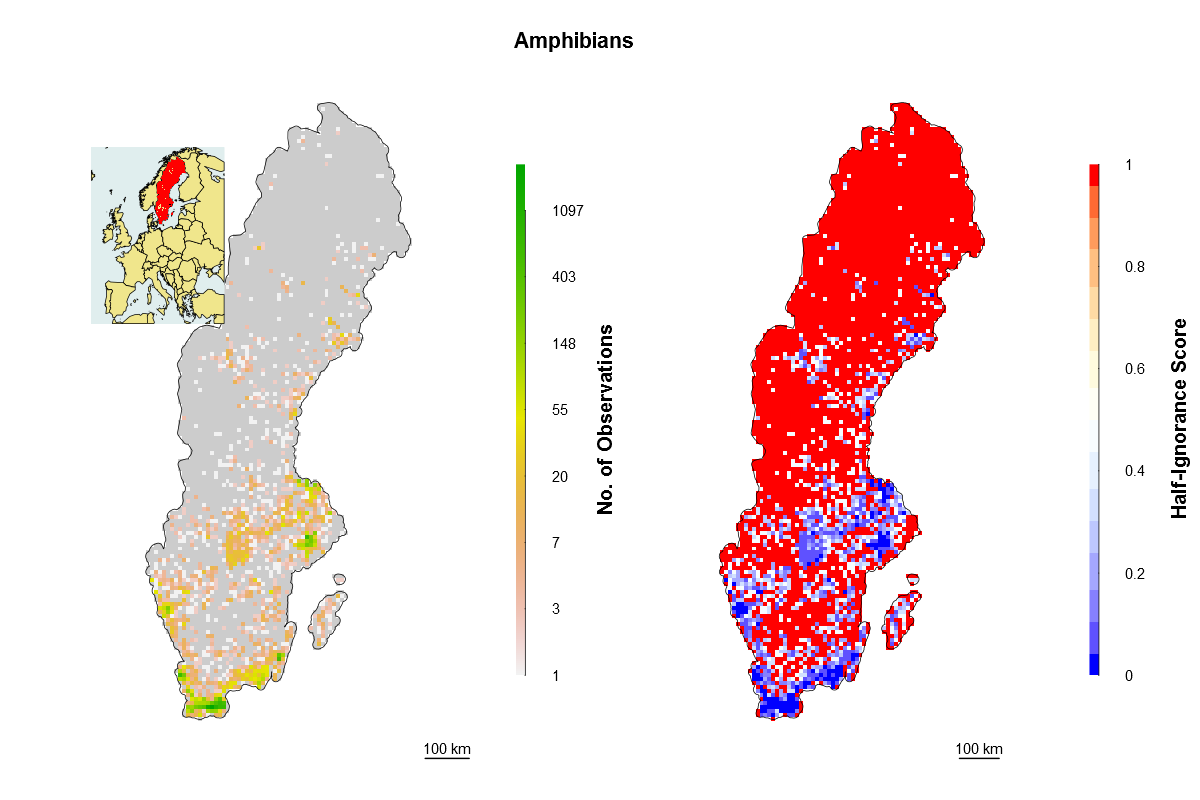


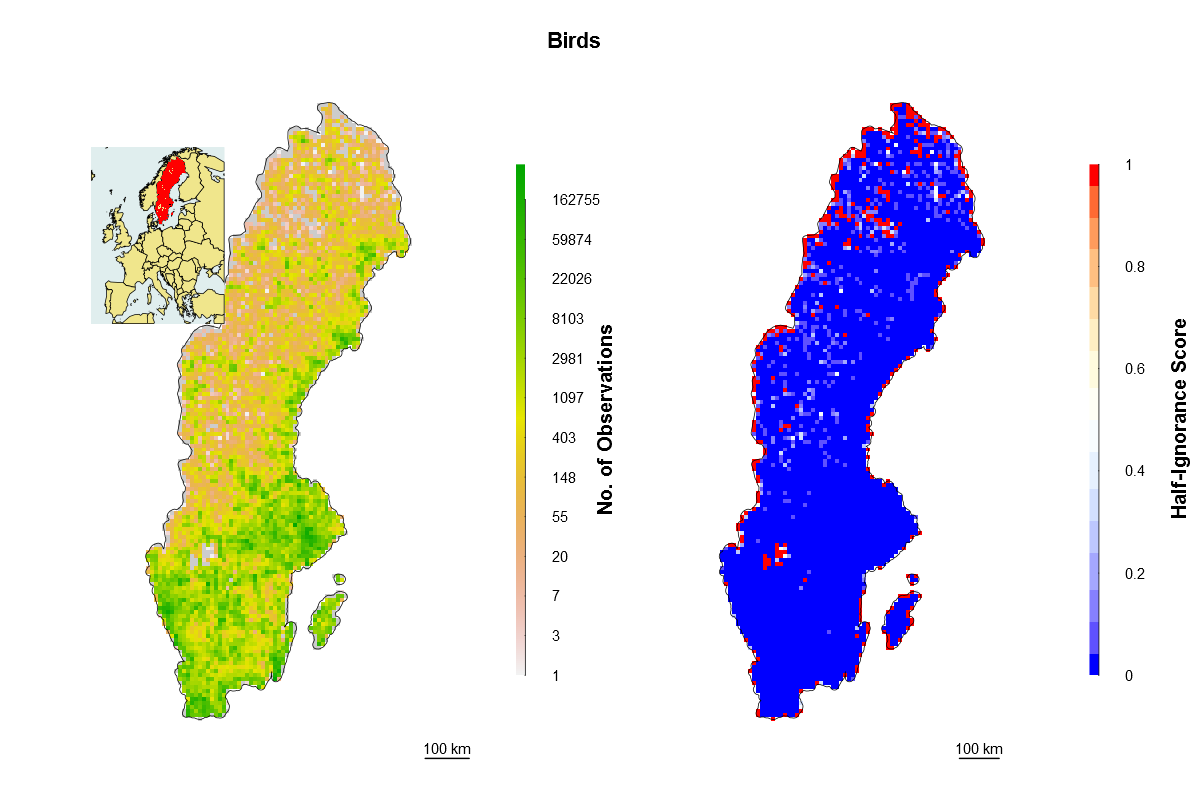


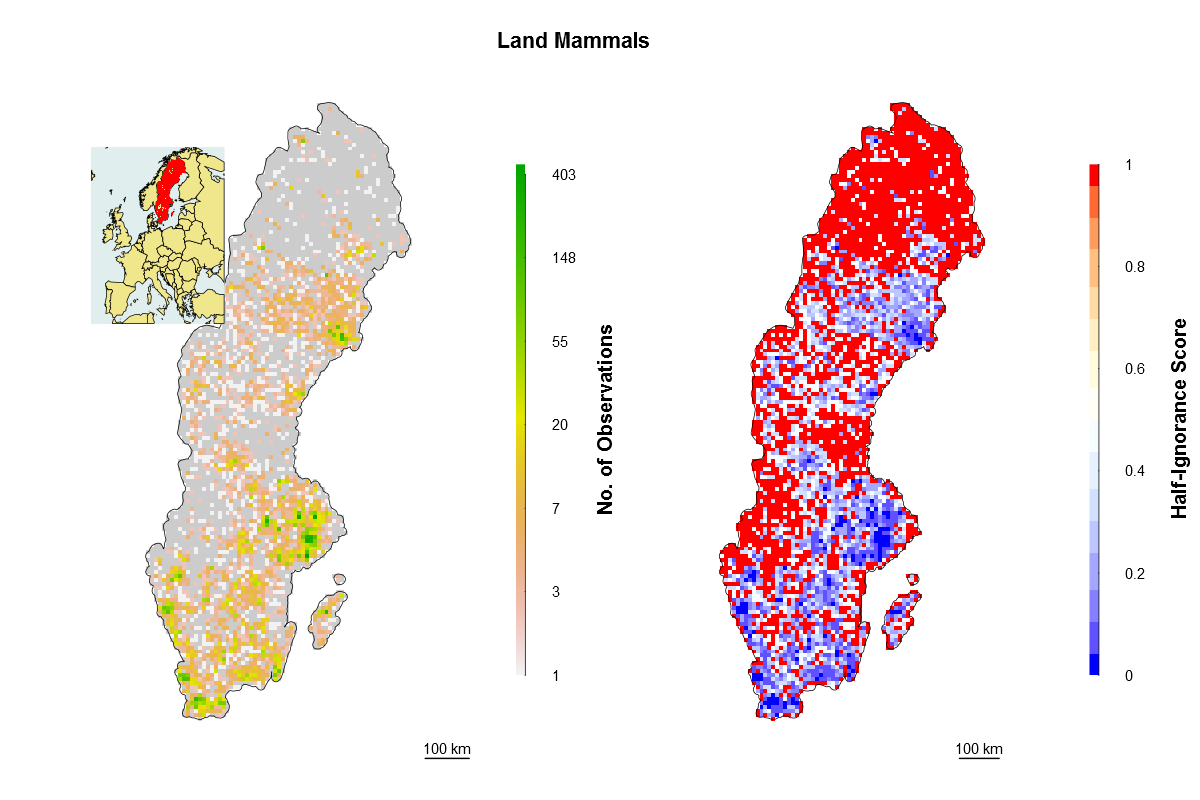


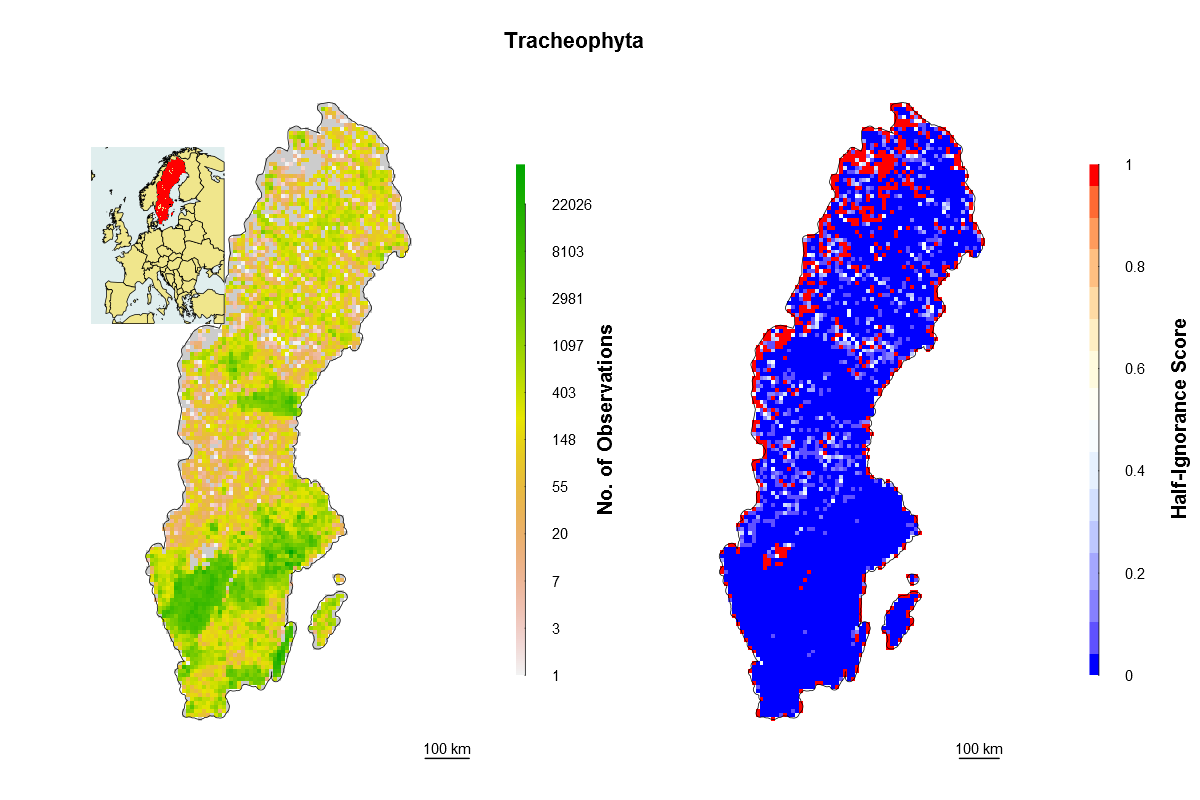


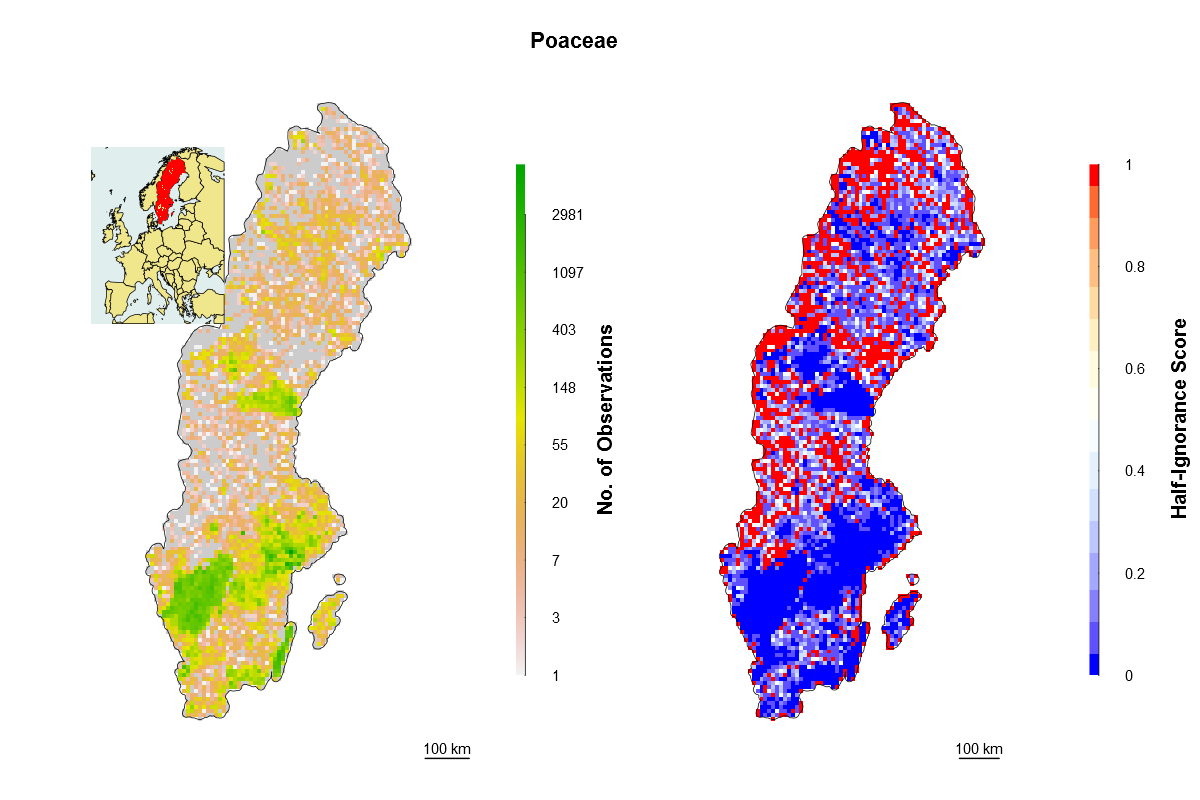


**S1 File Figure B**

**Figure B. The relationship between ignorance score and environmental variables.** The relationship between ignorance score and six environmental variables: elevation, slope, road density, path density, population density and log population density, for each taxonomic group studied at each level of O_0.5_ studied. Solid line indicates quadratic relationship, while dashed line indicates that only a linear relationship improved the model fit compared to the null model (only best models are drawn).

(a) O_0.5_ = 2


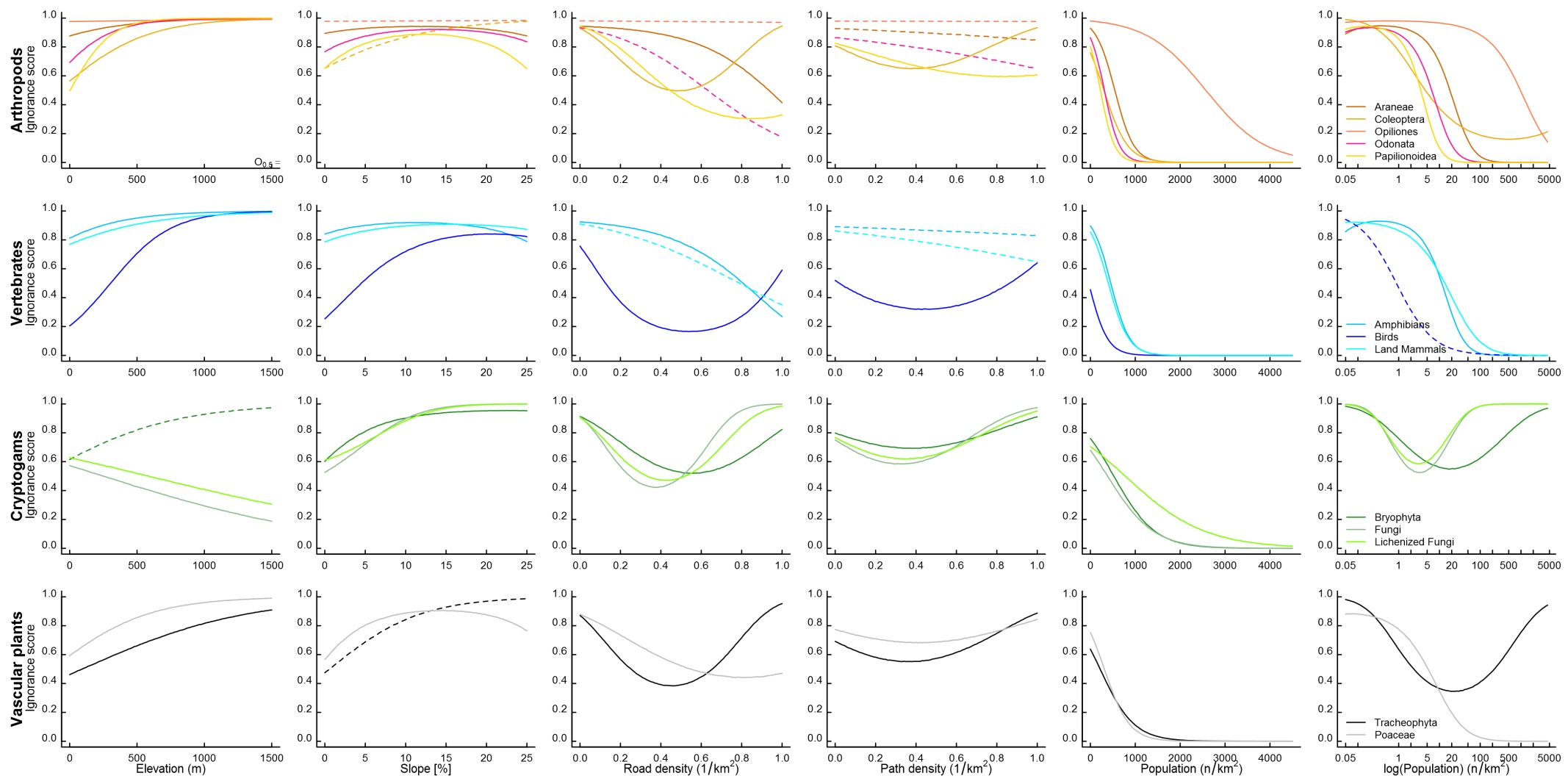


(b) O_0.5_ = 5


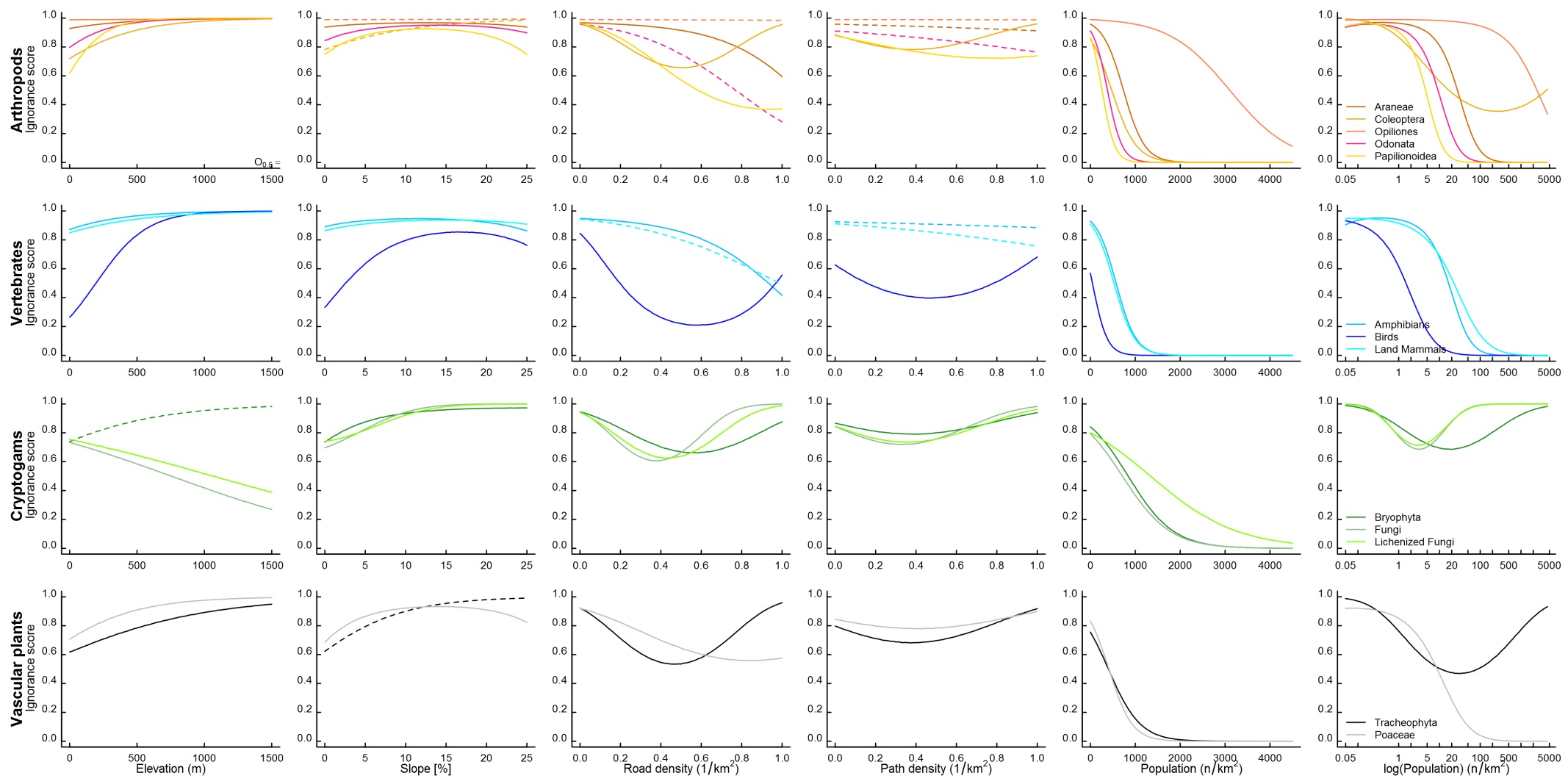


(c) O_0.5_ = 10


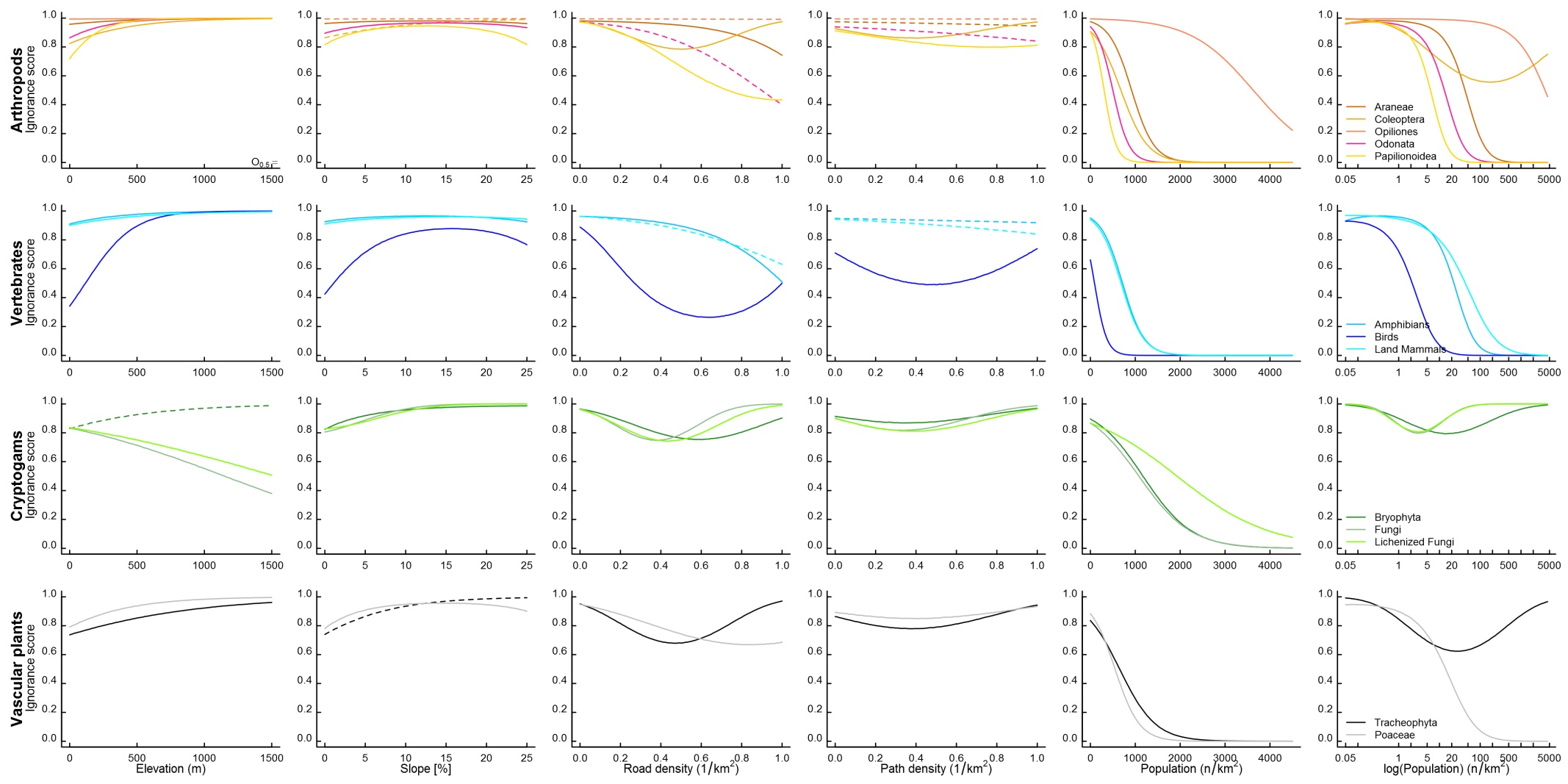


(d) O_0.5_ = 20


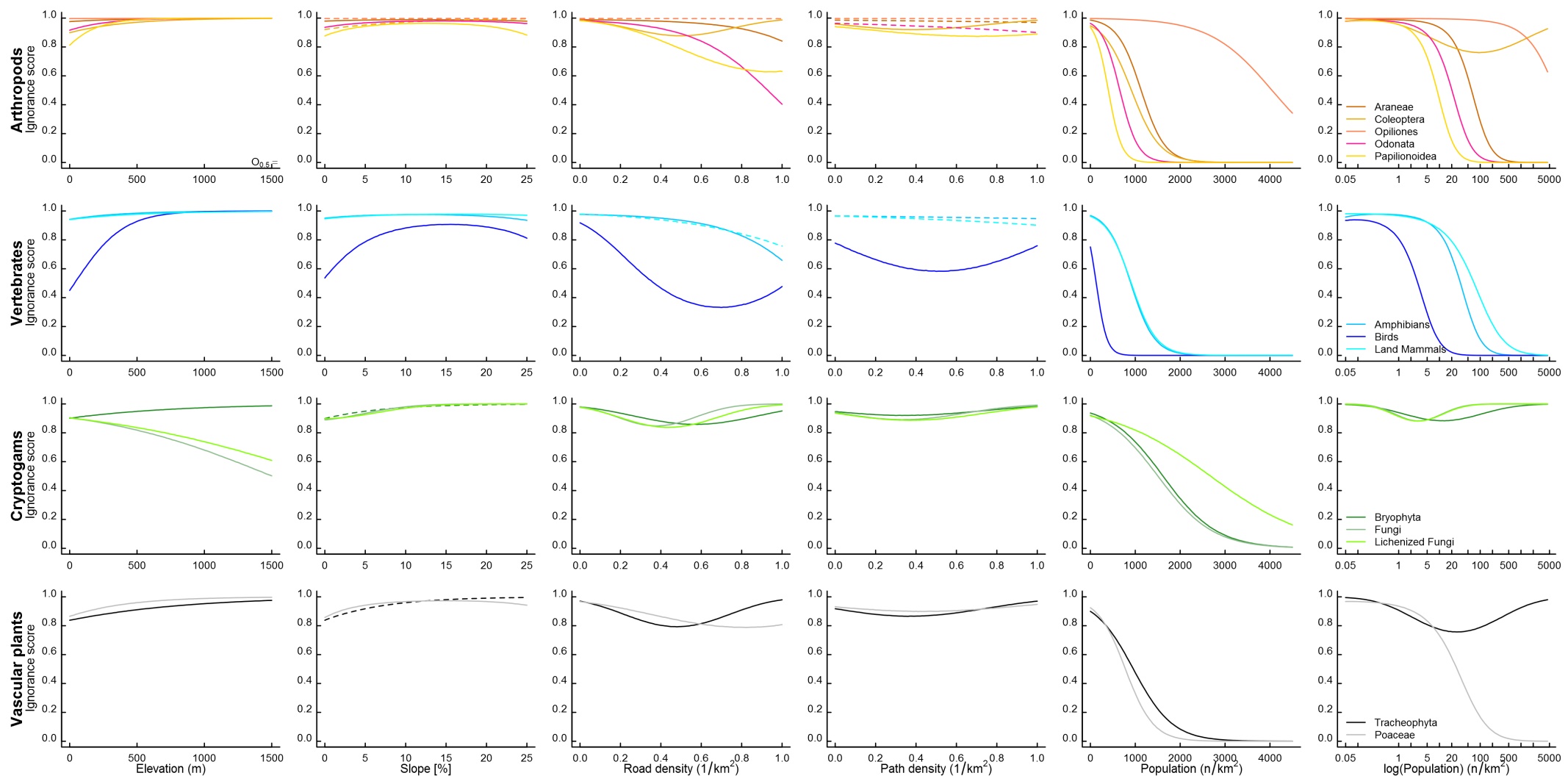


(e) O_0.5_ = 50


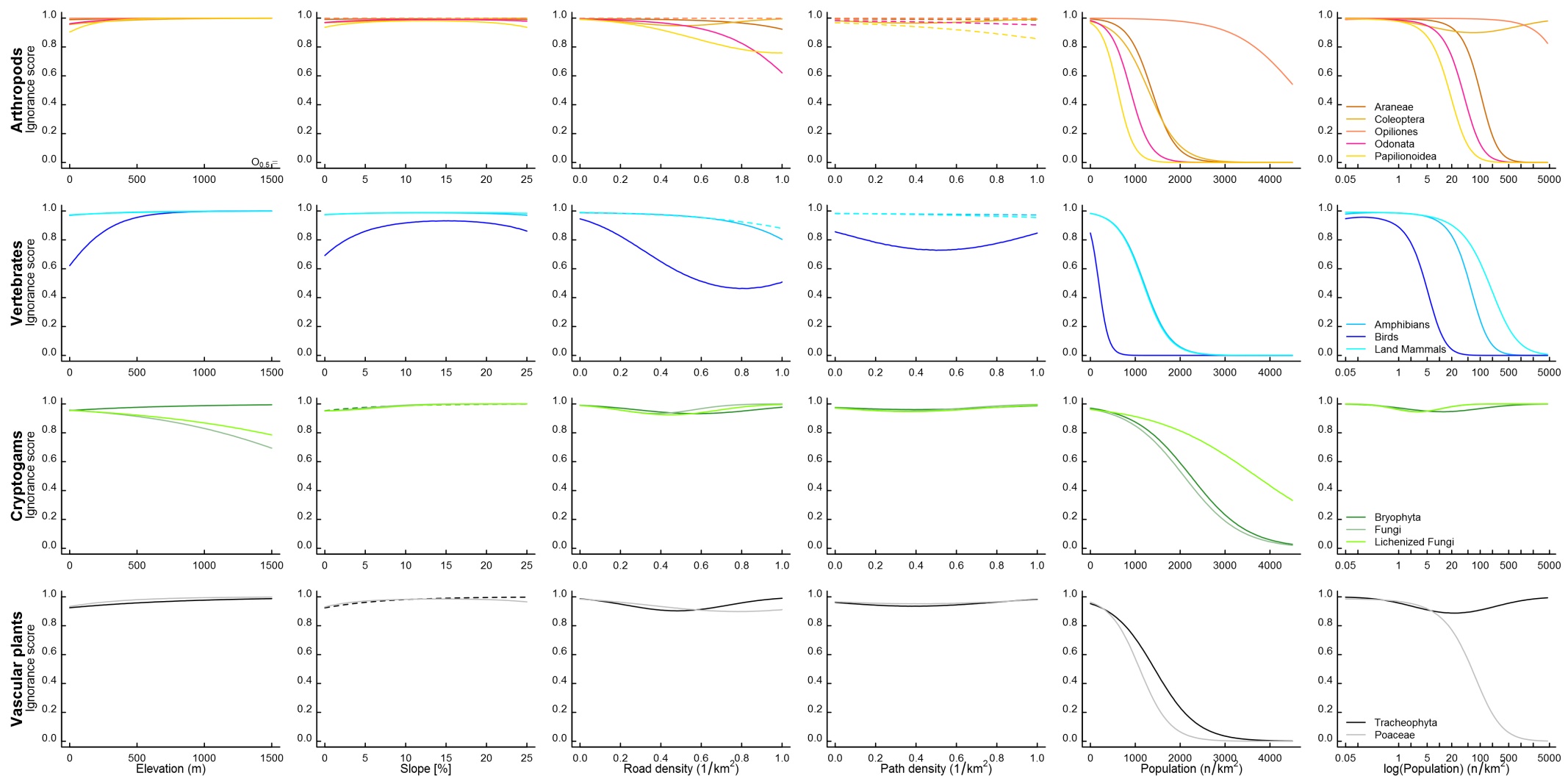

Supplement: S1 File — This file contains Table A (Percent deviance explained (R2) for all 13 reference taxonomic groups), Table B (Detailed model results for all 13 reference taxonomic groups), Fig A (Logged number of observation maps and ignorance score maps for all 13 reference taxonomic groups) and Fig B (The relationship between ignorance score and environmental variables). (DOCX) [file pone.0147796.s001.docx]
